# Synthesis of Antiviral Drug Tecovirimat and Its Key Maleimide Intermediates Using Organocatalytic Mumm Rearrangement at Ambient Conditions

**DOI:** 10.3390/ijms27010061

**Published:** 2025-12-20

**Authors:** Przemysław W. Szafrański, Wojciech Trybała, Adam Mazur, Katarzyna Pańczyk-Straszak, Alicja Kacprzak, Vittorio Canale, Paweł Zajdel

**Affiliations:** 1 Chair of Bioorganic Chemistry, Faculty of Pharmacy, Jagiellonian University Medical College, 9 Medyczna Street, 30-688 Kraków, Poland; wojciech.trybala@doctoral.uj.edu.pl (W.T.); adam.mazur1323@interia.pl (A.M.); katarzyna.panczyk@uj.edu.pl (K.P.-S.); alicja.kacprzak@student.uj.edu.pl (A.K.); vittorio.canale@uj.edu.pl (V.C.); pawel.zajdel@uj.edu.pl (P.Z.); 2 Doctoral School of Medical and Health Sciences, Jagiellonian University Medical College, 16 Łazarza Street, 31-530 Krakow, Poland

**Keywords:** tecovirimat, maleimide, Mumm rearrangement, organocatalysis

## Abstract

Tecovirimat is an antiviral agent approved for the treatment of orthopoxvirus infections including smallpox, cowpox and monkeypox. A key challenge in its synthesis lies in the generation of maleimide intermediates, which traditionally requires high-temperature thermal rearrangement and often results in low-to-moderate yields. Classical methods rely on heating in toluene above 70 °C, limiting scalability and efficiency. Herein, we present a mild and efficient organocatalytic approach to the synthesis of tecovirimat intermediates, using a room-temperature Mumm rearrangement of isomaleimide precursors. The reaction is catalyzed by 10 mol% imidazole and *N*-hydroxysuccinimide. As a representative example for one of the tecovirimat synthesis methods, intermediate *N*-(2,5-dioxo-2,5-dihydro-1*H*-pyrrol-1-yl)-4-(trifluoromethyl)benzamide was synthesized from *p*-trifluoromethylbenzohydrazide at a 71% yield over two steps. Additionally, *N*-(2,5-dioxopyrrol-1-yl)(*tert*-butoxy)formamide was obtained from Boc-hydrazide at a 37% yield. The methodology was sufficiently extended to other benzohydrazide-derived isomaleimides. To support the mechanistic rationale, preliminary PM7 semiempirical computational studies were performed, highlighting the electronic features facilitating the transformation. This work offers a practical and scalable route to tecovirimat intermediates, overcoming key synthetic bottlenecks and enhancing the efficiency of antiviral drug production.

## 1. Introduction

**Figure 1:**
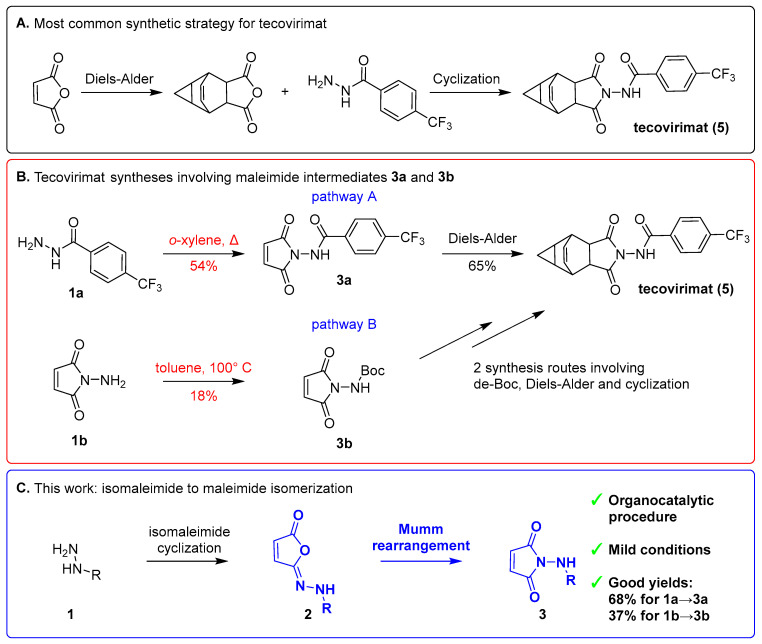
(**A**) Most common pathway of tecovirimat synthesis, involving Diels–Alder cycloaddition and thermal cyclization of the imide system [8]. (**B**) Tecovirimat synthesis pathways involving maleimide intermediates **3a** and **3b**. (**C**) Mumm rearrangement (in blue) of hydrazide (**1**)-derived isomaleimides (**2**) to maleimides (**3**) as a general strategy explored in our study.

Smallpox, a deadly viral disease, was declared extinct decades ago [1,2]. However, samples of the smallpox pathogen may still exist, posing the potential threat of a bioterrorist attack [1]. Moreover, strains of related viral diseases—cowpox and mpox (formerly known as monkeypox)—have become increasingly infectious and life-threatening [3,4,5]. These conditions paved the way for the development of tecovirimat (brand name TPOXX, Figure 1A), an antiviral agent targeting the orthopoxvirus family. Tecovirimat inhibits the VP37 protein, which is critical for the formation of mature virions that are capable of exiting the host cell [6,7].

The most common method for synthesis of tecovirimat employs maleic anhydride as the starting substrate and requires two ring-forming reactions: Diels–Alder cycloaddition and benzohydrazide–imide cyclization (Figure 1A). Reported alternative routes [8] are largely variations of these two steps. Two general pathways (Figure 1B) involve obtaining two maleimide intermediates, *N*-(2,5-dioxo-2,5-dihydro-1*H*-pyrrol-1-yl)-4-(trifluoromethyl)benzamide (**3a**) and *N*-(2,5-dioxo-2,5-dihydro-1*H*-pyrrol-1-yl)(*tert*-butoxy)formamide (**3b**). In pathway A (Figure 1B, top), intermediate **3a** is synthesized directly and subsequently undergoes a Diels–Alder cycloaddition. Pathway B (Figure 1B, bottom) starts from maleimide **3b** cyclization, followed by a sequence of Diels–Alder cycloaddition, Boc-deprotection and acylation reactions, executed in varying orders to yield tecovirimat.

The classical methods of maleimide synthesis used to make intermediates **3a** and **3b** (Figure 1B) typically involve thermal cyclization in toluene, providing desired products in poor-to-moderate yields [8]. This cyclization reaction proceeds via a two-step mechanism: initial formation of isomaleimide as the kinetic product, followed by a thermal rearrangement yielding the thermodynamically stable maleimide (Figure 1C). This isomaleimide-to-maleimide rearrangement is known as the Mumm rearrangement [9,10].

Herein, we demonstrate that maleimide derivatives **3a** and **3b** (Figure 1B) can be easily obtained in a two-step process involving isomaleimide formation followed by a room-temperature Mumm rearrangement (Figure 1C). For isomaleimide synthesis, we used methanesulfonyl chloride (MsCl), modifying a protocol that was recently developed by Croatt et al. [11] to avoid a large excess of sulfonyl chloride and eliminate the need for chromatographic purification. Following the initially observed spontaneous isomerization of aniline-based isomaleimides under these conditions [11] and inspired by reports utilizing excess *N*-hydroxysuccinimide (NHS) alongside a dehydrating agent in a maleimide synthesis protocol, we hypothesized that NHS combined with a mild base could serve as an organocatalytic system to promote the isomaleimide-to-maleimide rearrangement (Figure 1C). Accordingly, we performed room-temperature organocatalytic Mumm rearrangements using NHS and imidazole, successfully obtaining tecovirimat intermediates **3a** and **3b,** as well as several model maleimides **3c–e** and **3h**. Extending this concept, we also synthesized isosuccinimide intermediate **4** and converted it to tecovirimat. To further elucidate the structural requirements for room-temperature Mumm rearrangement, we performed preliminary computational studies for the synthesized isomaleimides by using semiempirical quantum chemistry (PM7 method).

## 2. Results and Discussion

### 2.1. Study Design

Our research strategy for the organocatalytic synthesis of maleimide intermediates **3a** and **3b** and tecovirimat via the Mumm rearrangement under ambient conditions was implemented through five sequential stages. First, we developed optimal reaction conditions for the synthesis of isomaleimides **2a** and **2b**. In the second step, we proceeded to develop reaction conditions for the low-temperature Mumm rearrangement of these model substrates to maleimides **3a** and **3b**. Next, we tested if the developed reaction conditions could be applied to tecovirimat synthesis through the Mumm rearrangement of isosuccinimide **4**. Fourth, the scope of the methodology was examined to determine its generality across a broader substrate range. Finally, we performed computational studies to correlate the electronic structure of the synthesized isomaleimides with their propensity to undergo isomerization to maleimide.

### 2.2. Optimization of Isomaleimide **2a** and **2b** Synthesis

The isomaleimide synthesis has been known for decades, but their instability has limited their exploration, resulting in fewer reported compounds compared to maleimides. The early protocols used alkyl chloroformates or dicyclohexyl carbodiimide as the dehydrating agents, under a low temperature [12].

**Figure 2:**
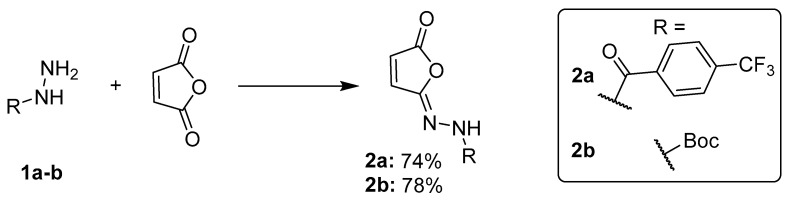
Synthesis of isomaleimides **2a** and **2b.** Optimized reaction conditions: for compound **2a**—1.5 eq. K_2_CO_3_ + 1 eq. TEA as base; for compound **2b**—2 eq. K_2_CO_3_. Both reactions used 1.05 eq. MsCl for isomaleimide cyclization and were performed in anhydrous CH_2_Cl_2_ at 0 °C—RT, overnight.

More recent studies have introduced alternative reagents, including dehydrating agents like propylphosphonic anhydride (T3P) [13] or MsCl [11]. We found that the MsCl protocol, described by Croatt et al. [11], was a good starting point for optimization towards mild and efficient synthesis of tecovirimat intermediates without heating. The goal of this research stage was to obtain high purity isomaleimides **2a** and **2b** from respective hydrazides (Figure 2) as substrates for the Mumm rearrangement, without the need for chromatographic purification.

Using isomaleimide **2a** as a model compound, we evaluated various reaction conditions on a 100–200 mg scale (Table 1). Initial experiments revealed that excess triethylamine (TEA) in combination with MsCl facilitated rapid isomaleimide ring closure. However, excess TEA promoted side reactions which negatively impacted the product purity, while excess MsCl required removal by column chromatography. To address this, we minimized the amount of MsCl to the 1.05 equivalent and optimized the “basic medium” by reducing the amount of TEA and introducing potassium carbonate as a co-base. To reduce the risk of thermal isomaleimide-to-maleimide isomerization during workup, we changed ethyl acetate to lower-boiling dichloromethane, which can be easily evaporated without heating. This yielded cleaner ring closure; however, it limited the solubility of the intermediate fumarates in CH_2_Cl_2_. The optimal ratio of the bases used, in terms of reaction yield, product purity and intermediate solubility, was 1.5 eq. K_2_CO_3_/1 eq. TEA (Table 1, in bold).

Based on this optimization, we developed a standard procedure to be used in further research. The hydrazide was dissolved in anhydrous dichloromethane and reacted with maleic anhydride in the presence of K_2_CO_3_ and TEA (1.5/1 eq.) at 0 °C, followed by the addition of MsCl under similar conditions. The reactions then progressed overnight and extraction workup (NaHCO_3_, water) followed. Adjustments to the base were made depending on the solubility of intermediates (compound **2b**). Preparative scale reactions were performed for both tecovirimat intermediates (compounds **2a** and **2b**). Compound **2a** was obtained by using K_2_CO_3_/TEA (1.5 eq./1 eq.) as a base with a maximum yield of 79.2% (0.44 g).

For compound **2b**, we made several successful gram-scale attempts, using only K_2_CO_3_ (2 eq.) as a base, because the **1b**-derived intermediate amido acid potassium salt has better solubility in organic media than those of arylhydrazides like **1a**, which required a TEA addition. The compound was obtained at a maximum yield of 74% (2.1965 g of **2b** out of 1.8533 g of hydrazide **1b**) without the need for further purification (UPLC-MS 98% purity). During scaling-up optimization, we also found that under the developed conditions, isomerization of isomaleimide to maleimide is possible. In a reaction on a 3 g scale (starting hydrazide), we obtained a crude mixture, containing both isomaleimide (55%, HPLC) and maleimide (33%, HPLC). For tecovirimat synthesis, this may be an advantageous phenomenon. If it is necessary to obtain pure isomaleimide, the possibility of potential isomerization should be taken into account, which requires avoiding heating and heat generation at all stages, including reduced pressure evaporation.

### 2.3. Development of Low-Temperature Mumm Rearrangement Conditions

Despite the longstanding knowledge of the Mumm rearrangement, surprisingly few documented examples of the isomaleimide-to-maleimide isomerization can be found. Papers which explicitly describe a process in which an isomaleimide is isolated, characterized and subsequently converted into maleimide are scarce: for example, thermal isomerization of *N*-benzyl isomaleimide with a 0.4 equivalent of HOBT used as catalyst [14]. There is also one entry in the patent literature in which a disubstituted isomaleimide is converted to maleimide through heating in acetic acid at 120 °C. Apart from that, one paper can be found in which aza-isosuccinimides are converted to the corresponding aza-succinimides in a two-step decarboxylation–carboxylation process, which is possible due to the nitrogen atom in the β-position from the carbonyl group [15]. In other cases, the isomaleimide formation step is treated implicitly, or like in tecovirimat-related patents, isomaleimide is reported as a byproduct [8].

**Figure 3:**
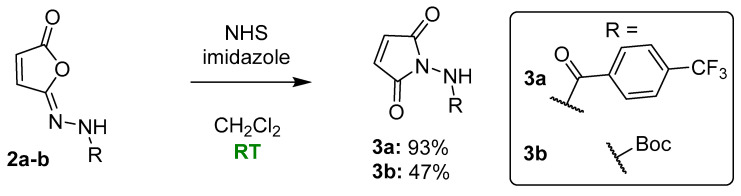
Organocatalytic Mumm rearrangement of isomaleimides **2a** and **2b** to tecovirimat intermediates **3a** and **3b**. For **3a**, isomerization required 10 mol% of NHS and imidazole, while for **3b**, the isomerization required 30 mol% of both catalysts.

To bridge this gap in the knowledge and fulfill the objective of synthesizing tecovirimat intermediates **3a** and **3b** from respective isomaleimides, we optimized the reaction conditions for the organocatalytic Mumm rearrangement (Figure 3).

Upon noticing the isomerization propensity of isomaleimides **2a** and **2b,** even under mild synthesis conditions, and considering hints from the literature, including the use of 0.4 eq. HOBT at an elevated temperature [14] or the use of excess *N*-hydroxysuccinimide (NHS) in direct maleimide synthesis protocols [16], we decided to use NHS and a mild organic base to facilitate the isomerization. Our studies on isomaleimide **2a** synthesis indicated that adding TEA might result in impurity generation; therefore, we sought a milder base. We tested *N*-methylmorpholine (NMM) and imidazole alone, as well as their combinations with NHS.

For screening, we used isomaleimide **2a** in analytical-scale (5 mg) experiments: we dissolved **2a** in anhydrous dichloromethane (1 mL) and added solutions of the respective catalysts (5 mol% each) alone or in combination and conducted reactions for 24 h at room temperature. After that, we evaporated dichloromethane and redissolved the residue in CDCl_3_ for ^1^H NMR measurement, which we used to assess conversion (Table 2).

The optimal progress of isomerization (99%) was achieved using NHS and imidazole in equimolar proportions (Table 2, in bold). NHS alone gave slightly lower conversion (92%), while its combinations with TEA or NMM gave poorer results (80%). Imidazole alone allowed us to reach only 50% isomerization and NMM did not promote isomerization at all. After selecting the NHS–imidazole system, we performed several preparative reactions on a 100–200 mg scale and found that the catalysts can be removed by mild basic extraction to yield a clean product (93–95% HPLC purity). We found that isomerization efficacy and final product purity depended greatly on substrate quality: impurities were from isomaleimide synthesis.

The optimization stage resulted in the development of a standard procedure used in reaction scaling for both tecovirimat intermediates (compounds **3a** and **3b**). The isomaleimides were dissolved in CH_2_Cl_2_; NHS and imidazole were then added (10 mol%) and the reaction was carried out at room temperature (HPLC monitoring). In case of slow or no isomerization, the amounts of catalysts were increased by up to 30 mol% (also by adding catalysts during the reaction).

Compound **3a** was obtained using a 10 mol% catalyst, loading within 4 h for 93% yield (207 mg of product; HPLC 99.5% t_R_ = 1.61 min). Purification of the product by column chromatography (AcOEt/Petroleum ether, 2:1) was also possible if needed.

Compound **3b** required 30 mol% catalysts, loading and carrying out the reaction overnight. It obtained a 47% yield (248 mg of purple crude product; HPLC 93.6% t_R_ = 1.30 min). After purification of the product by column chromatography (AcOEt/Petroleum ether, 1:1), a pure product was obtained (UPLC-MS 97% purity), but the yield decreased to 20%. Based on ^1^H NMR comparison (crude **3b** vs. the purified one), we found that the crude product could be used for further synthesis of tecovirimat. Column chromatography, on the other hand, can be easily used if a higher purity of the product is desired.

### 2.4. Synthesis of Tecovirimat from Maleimide **3a**

**Figure 4:**
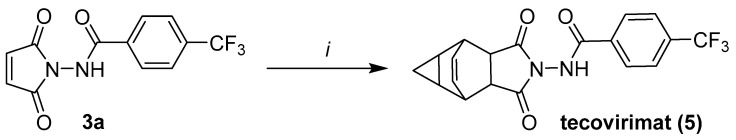
Synthesis of tecovirimat from maleimide **3a**, according to reaction conditions described in the patent literature [17]. Reaction conditions: *i—*cycloheptatriene, anhydrous toluene, nitrogen atmosphere, 25 h and 110 °C.

To demonstrate the usefulness of the obtained maleimide intermediates, we synthesized tecovirimat **5** from maleimide **3a** (Figure 4), obtained through an organocatalytic Mumm rearrangement. The final Diels–Alder cycloaddition was performed, according to a procedure that was analogous to the one described in the patent literature [17], involving heating maleimide **3a** with cycloheptatriene to 110 °C in anhydrous toluene under a nitrogen atmosphere for 25 h. The reaction proceeded to completion within the planned time, and the product required chromatographic purification, as it was described in the patent literature, which we completed by using automated flash chromatography with gradient elution. We obtained tecovirimat at a 56% yield (lower than the 65% yield described in the patent literature [17]); however, the cumulative yield of the process (pathway A, Figure 1B) was higher (38% vs. 35% in the patent literature), due to a better yield of **3a** synthesis through the Mumm rearrangement.

### 2.5. Synthesis of Tecovirimat Through Isosuccinimide Rearrangement

**Figure 5:**
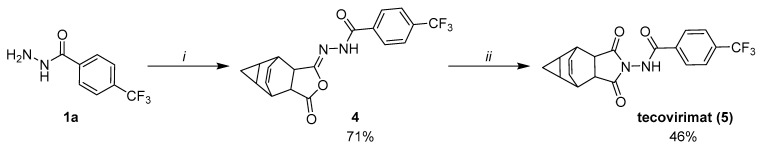
Synthesis of tecovirimat through room-temperature isomerization of isosuccinimide **4**. Reaction conditions: *i—*1.5 eq. K_2_CO_3_, 1 eq. TEA, 4-oxatetracyclo [5.3.2.02,6.08,10]dodec-11-ene-3,5-dione, 2 h, 0–5 °C, CH_2_Cl_2_ then MsCl, overnight, 0 °C–RT; *ii—*10 mol% NHS, 10 mol% imidazole, CH_2_Cl_2_, overnight, RT.

Extending the scope of the methodology developed herein, we wanted to test whether it is possible to obtain tecovirimat through a room temperature Mumm rearrangement of isosuccinimide **4** (Figure 5). We synthesized compound **4** from hydrazide **1a** by using the K_2_CO_3_-TEA combination, like compound **2a**. The yield was good (71%), but the product purity determined by UPLC-MS was limited (65%). We reacted **4** with catalytic amounts of NHS and imidazole (10 mol%), which gave tecovirimat at a 46% yield after chromatographic purification. The overall yield of this two-step process was 33%, which was considerably lower than the most recent tecovirimat syntheses published [18,19]. The reduced yield of the last step and the whole process could result from the limited purity of isosuccinimide **4** and the formation of further impurities upon isomerization: most notably, the uncyclized amido acid, which can also be found as a tecovirimat synthesis impurity (impurity 1c, [20]). The uncyclized amido acid can result from incomplete cyclization of **4** or its decomposition upon isomerization. It was also formed in large amounts when chromatographic purification of **4** was attempted. An important source of yield reduction was also the chromatographic purification of tecovirimat.

For the first time, we demonstrated that a low-temperature synthesis of tecovirimat is possible through a novel isosuccinimide intermediate **4**. Moreover, these experiments also proved that a room temperature Mumm rearrangement is possible for benzohydrazide-derived isosuccinimides. However, the limited yield, chromatographic purification and large amount of CH_2_Cl_2_ would need to be improved to develop larger-scale processes.

### 2.6. Spectrum of Application of the Developed Synthetic Methodology

**Figure 6:**
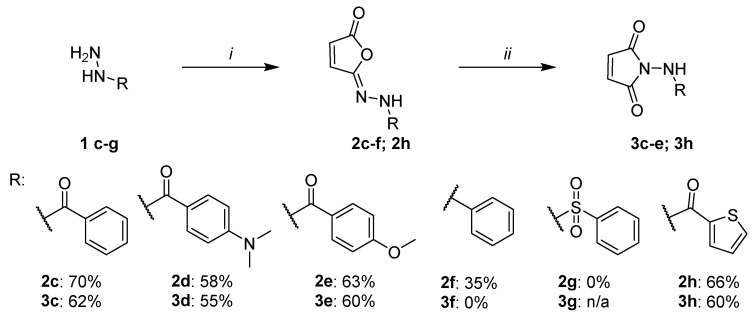
Scope of the developed synthetic methodology. Reaction conditions: *i—*maleic anhydride (1 eq.), K_2_CO_3_/TEA (1.5 eq./1 eq.) for **2c**–**f**; K_2_CO_3_ (2 eq.), for **2g**, K_2_CO_3_/TEA (1 eq./1.5 eq.) or imidazole (3.5 eq.), MsCl (1 eq.), CH_2_Cl_2_ 0 °C to RT, overnight; *ii—*NHS, imidazole (10 mol% for **3c**–**e**, **3g**–**h**, 30 mol% for **3f**), CH_2_Cl_2_, RT, overnight.

Next, the developed methodology was applied to the synthesis of structurally diverse isomaleimide and maleimide derivatives (Figure 6). We have already demonstrated its applicability for aromatic derivatives bearing a deactivating –CF_3_ substituent in the *para* position (**2a** and **3a**) and selected aliphatic compounds (**2b** and **3b**). The current investigation focuses primarily on aromatic systems, due to their wide applicability in medicinal chemistry and improved performance, particularly in the isomerization step from isomaleimide to maleimide. Figure 6 illustrates the extended scope of the study.

At first, the influence of the presence and type of substituent in the aromatic ring was examined (compounds **2c**–**2e** and **3c**–**3e**). Due to their potential applications in medicinal chemistry (e.g., synthesis of amines and sulfonamides), phenylhydrazine (compounds **2f** and **3f**) and benzenesulfohydrazide (compounds **2g** and **3g**) derivatives were included. We also tested one heterocyclic system, the tiophenecarbonyl hydrazide (compounds **2h** and **3h**). While the tiophenecarbonyl hydrazide **1h** is a well-known medicinal chemistry building block [21,22], its isomaleimide (**2h**) and maleimide derivatives (**3h**) have not been described before.

To obtain the desired compounds, the optimized synthetic procedures for compounds **2a** and **3a** were initially utilized, as shown in Figure 6. Due to the lack of reaction progress under standard conditions for compound **3f**, the amount of catalyst was increased to 30 mol%. For similar reasons, an attempt to synthesize compound **2g** was carried out by using various basic conditions: K_2_CO_3_ (2 eq.), K_2_CO_3_/TEA (1 eq./1 eq.) or imidazole (3.5 eq.).

The obtained results (Figure 6) confirm the applicability of the developed methodology for the synthesis of isomaleimides in the case of variously substituted benzohydrazide derivatives **2c**–**2e**, as well as the phenylhydrazine derivative **2f** and the tiophenecarbonyl hydrazide-based compound **2h**. In contrast, the desired product could not be obtained for the benzenesulfonohydrazide derivative **2g** under the applied reaction conditions, due to the formation of numerous unidentified by-products (HPLC monitoring). Consequently, further attempts to synthesize this class of compounds were discontinued.

The yields obtained for benzohydrazide derivatives **2a** and **2c**–**2e** ranged from 58 to 79%. A slight influence of the type of substituent in the aromatic ring on the reaction yield was observed, with the benefit of the deactivating substituents: -CF_3_ (**2a**, 79%) > -H (**2c**, 70%) > -OCH_3_ (**2e**, 63%) > -N(CH_3_)_2_ (**2d**, 58%). Isomaleimide derivatives were treated as intermediates in the synthesis of maleimides. For this reason, in the case of obtaining a crude product with a purity above 85%, no further purification of the product was carried out. Benzohydrazide derivatives **2a** and **2c**–**2d** had a purity of >95% (UPLC-MS), while derivative **2e** had a purity of only 85.7% (UPLC-MS). This fact should be taken into account in the assessment of the substituent effect and the yields of compounds **2d** and **2e** should be considered comparable. The isomaleimide derivative of phenylhydrazine (**2f**) required purification on a chromatographic column and was finally obtained at a 35% yield. The possibility of using the developed methodology for the synthesis of amine derivatives, not only amide ones, is noteworthy.

The obtained isomaleimides **2c**–**e** and **2h** were isomerized to maleimide derivatives **3c**–**e** and **3h**. The final products were obtained for all benzohydrazide derivatives and the tiophenecarbonylhydrazide derivative. The developed methodology turned out to be ineffective in the case of the amine derivative **3f** (no reaction progress, starting from isomaleimide **2f** after 24 h and the addition of 30 mol% catalysts).

All achieved compounds were purified by column chromatography. The final yields for the aromatic derivatives **3c**–**3e** and **3h** ranged from 55 to 62% (Figure 6). Likewise, for isomaleimide derivatives, the influence of the type of substituent on the aromatic ring on the yield was analyzed. A significantly higher yield was observed for the deactivating substituent –CF_3_ (**3a**, 93%, Figure 3). In other cases, the performance was comparable: -H (**3c**, 62%) > -N(CH_3_)_2_ (**3d**, 55%) > -OCH_3_ (**3e**, 60%). Moreover, the significant impact of a properly performed workup procedure on the final yields was noticed. Due to the relatively low lipophilicity of maleimide derivatives, the use of a large dilution of the organic phase before washing with an aqueous layer (optimally five times the reaction volume) allowed us to increase the final yield by approximately 40%. This technique was ineffective in the synthesis of isomaleimides.

### 2.7. Structural Basis of Low-Temperature Mumm Rearrangement: Preliminary Computational Studies

To explore the structural basis of the room-temperature Mumm rearrangement, we inspected the electronic structures of isomaleimides **2a**–**f, 2h** and isosuccinimide **4**, using semiempirical quantum chemistry. To achieve that, for each compound we performed conformational searches with a genetic algorithm combined with molecular mechanics (MMFF94 forcefield) and subsequent geometry optimizations, using the semiempirical PM7 method [23]. For each lowest-ΔH_f_ conformation, we performed population analysis to determine charge distribution and visualized selected molecular orbitals: in particular, the LUMO orbitals, whose energy can determine the susceptibility to nucleophilic attack by *N*-hydroxysuccinimide.

**Figure 7:**
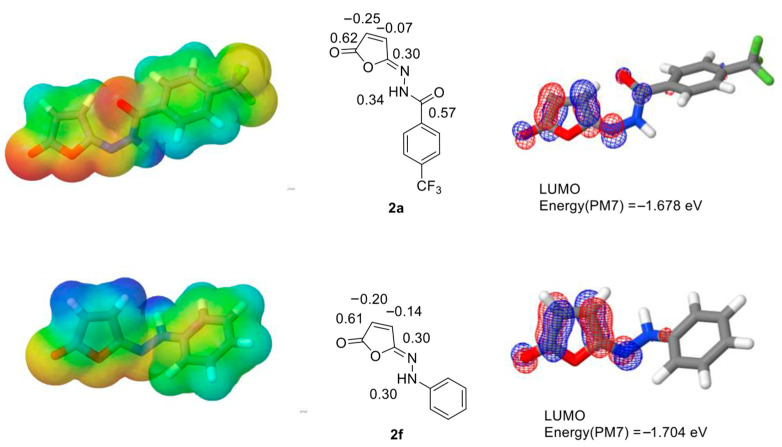
PM7 semiempirical quantum chemistry computation results for isomaleimides **2a** (isomerizing) and **2f** (non-isomerizing). **Left**: Lowest-ΔHf conformations with electrostatic potential surfaces; **center**: structures with selected net charges marked (carbonyl groups, double bond and hydrazine hydrogen); **right**: mesh representation of the LUMO orbital.

Performing the computations, we wanted to compare the benzohydrazide derivatives **2a**–**e**, for which the isomerization proceeded efficiently, with phenylhydrazine-based isomaleimide **2f**, which did not rearrange to maleimide under the proposed reaction conditions. We found that the investigated isomaleimides did not differ significantly in LUMO orbital energy or charge distribution. All had a relatively low-energy LUMO orbital (see Table 3 for energies) located around the electron-deficient C-C double bond, extending towards the hydrazine moiety, with some contribution at the amide carbonyl group (Figure 7). In the non-isomerizing hydrazine derivative **2f**, the hydrazine N-H atom had sp^3^-like geometry (Figure 7), whereas in hydrazide derivatives **2a**–**e**, **2h** and **4**, it had flat sp^2^-like structure. In all these cases, the positive charges (0.34–0.37) of the N-H proton indicated its acidity, which was noticeably lower for the non-isomerizing compound **2f** (0.30 net charge). The LUMO energies were low (from −1.8 to −1.5 eV) and did not correlate with the isomerization tendency.

A small, but notable, difference that correlated well with the isomerization tendency of the benzohydrazide and phenylhydrazine derivatives **2a**–**f** was the charge distribution in the C-C double bond: in compounds that isomerized efficiently, the carbon atom closer to the C=N double bond had a significantly lower negative charge than the other one: −0.07 vs. −0.25 in compound **2a**, similarly for **2b**–**e**. In the non-isomerizing phenylhydrazine derivative **2f**, this difference was less significant (−0.14 vs. −0.20), and the carbon next to the C=N double bond had a higher negative charge (−0.14) compared to the isomerizing compounds. In the compound **2h**, with an electron-rich tiophene ring attached to the hydrazide, the charge distribution in the double bond (−0.12 and −0.21) was a little closer to the non-isomerizing phenylhydrazine derivative **2f**.

This can indicate that the electronic structure of the electron-deficient C=C double bond can play a role in isomaleimide isomerization propensity: however, the acidity of the hydrazide proton seemed to have a stronger correlation with isomerization, as in all the isomerizing compounds, the positive charge was higher than in the non-isomerizing phenylhydrazine derivative.

The isosuccinimide **4**, devoid of this C=C double bond, shared some electronic features like hydrazide proton charge (0.36) and higher positive charge of the carbonyl group compared to the imine group (0.6 vs. 0.4). The orbital structure of **4** is significantly different, with higher LUMO energy and a different distribution. Unoccupied orbitals covering the isoimide moiety located higher (second and third unoccupied orbital) and have much higher energy than compounds **2a**–**f** (see Figures S22–S26 in the Supplementary Materials).

### 2.8. Study Limits

Despite the promising outcomes of this work, including maleimides **3a**–**e, 3h** and tecovirimat (**5**), certain limitations should be acknowledged in order to contextualize the scope and reliability of the present findings. For phenylhydrazine **2f**, the rearrangement produced a complex product mixture. For benzenesulfonamido hydrazide **2g**, we failed to synthesize isomaleimide, so the final maleimide, **3g**, was not obtained as well. For hydrazine-based isomaleimide **2f**, based on preliminary computational studies and experimental results, we could hypothesize that the lack of isomerization can be attributed to the lower acidity of the N-H proton and changes in double bond electronic structure. For the sulfonyl hydrazide **1g**, the problem in obtaining isomaleimide could be related to the significantly higher acidity of the N-H proton, which is generally observed in sulfonamide derivatives.

An important limitation of the described methodology at the present stage is in the scale and solvent. For the present, initial study, we chose CH_2_Cl_2_ as a convenient solvent for laboratory use, particularly for easy evaporation at room temperature, which is important for isoimide synthesis. As CH_2_Cl_2_ should not be used on a manufacturing scale due to the environmental impact and hazardous concerns, further solvent studies will be required to find optimal large-scale solvents or to find other ways of overcoming the solvent problem.

## 3. Materials and Methods

### 3.1. General Methodology

Reagents and solvents. All starting materials and solvents (purchased from Merck (Rahway, NJ, USA), Tokyo Chemical Industry (Tokyo, Japan), Fluorochem (Hadfield, UK) or Chempur (Karlsruhe, Germany)) were of reagent grade and used without further purification. Anhydrous CH_2_Cl_2_ was prepared by distillation and drying over 4A molecular sieves overnight. Anhydrous toluene for the Diels–Alder reaction was obtained by distillation from calcium hydride under an inert atmosphere and stored over activated 4 Å molecular sieves overnight prior to use. Column chromatography was performed using silica gel Merck 60 (70–230 mesh ASTM).

### 3.2. Nuclear Magnetic Resonance (NMR) Spectroscopy

The ^1^H NMR (500 MHz) and ^13^C NMR (126 MHz) spectra were taken in CDCl_3_ or DMSO-*d*_6_ and recorded on a 500 MHz JEOL JNM-ECZR500 RS1 spectrometer (JEOL Ltd., Akishima, Tokyo, Japan) at the Chair of Organic Chemistry, Faculty of Pharmacy, Jagiellonian University Medical College. The results are presented in the following format: chemical shift δ (ppm), multiplicity, *J* values in Hertz (Hz), number of protons/carbons and protons’/carbons’ position. Multiplicities are shown as the following abbreviations: s (singlet), bs (broad singlet), d (doublet), dd (doublet of doublets), dt (doublet of triplets), t (triplet) and m (multiplet).

### 3.3. Automated Flash Chromatography

Automated flash chromatography separations were performed using a CombiFlash NextGen 300+ flash chromatography system (Teledyne ISCO, Lincoln, NE, USA) with UV-VIS detection at two wavelengths: λ1 = 254 nm, λ2 = 280 nm. For tecovirimat (5), the separations were performed by using a hexane/ethyl acetate eluent system in gradient mode (9:1 to 4:6 over 39 min), on RediSep Gold 24g (Teledyne ISCO, Lincoln, NE, USA; 25 mL/min flowrate) or RediSep Gold 4g (13 mL/min flow rate) silica gel cartridges. For maleimide 3h, the separation was performed using a CH_2_Cl_2_/methanol eluent system in gradient mode (0*–*10% *v*/*v* methanol over 13 min), on RediSepGold 4g (13 mL/min flowrate).

### 3.4. High-Performance Liquid Chromatography (HPLC)

HPLC analyses were performed on an Arc HPLC Core System (Waters Corporation, Milford, MA, USA) equipped with a UV/Vis Waters 2998 PDA spectrophotometric detector (200*–*800 nm range, 1.2 nm resolution). Chromatographic separations were carried out using a 4.6 × 50 mm and 1.7 μm particle size Chromolith SpeedROD RP 18 column. The column was maintained at 40 °C and eluted with 3 mL/min water: acetonitrile mixture, with 0.1% formic acid as the acidic modifier, under gradient conditions (5–100% acetonitrile over 3 min). Chromatograms were extracted as MaxPlot traces (the detector value at each time point equals the maximum absorbance across the scanned 200–800 nm wavelength range), using Waters Empower 3.7.0 software (Waters Corp., Milford, MA, USA).

### 3.5. Ultra-Performance Liquid Chromatography–Mass Spectrometry (UPLC-MS)

The UPLC-MS/MS system (Waters Corporation, Milford, MA, USA) consisted of a Waters Acquity Premier coupled with a Waters Xevo TQ-S Cronos mass spectrometer (electrospray ionization mode ESI). UPLC-MS spectra were obtained as a service at the Center for the Development of Therapies for Civilization and Age-Related Diseases, Jagiellonian University Medical College. Chromatographic separations were carried out using the Acquity UPLC BEH (bridged ethylene hybrid) C18 column—2.1 × 100 mm and 1.7 µm particle size—equipped with Acquity UPLC BEH C18 VanGuard pre-column—2.1 × 5 mm and 1.7 µm particle size. The column was maintained at 40 °C and eluted under gradient conditions using 95% to 0% of eluent A over 10 min, at a flow rate of 0.3 mL/min. Eluent A: water/formic acid (0.1%, *v*/*v*); eluent B: acetonitrile/formic acid (0.1%, *v*/*v*). Chromatograms were recorded using Waters eλ PDA detector. Spectra were analyzed in a 200–500 nm range with 1.2 nm resolution and sampling rate 20 points/s. MS detection settings of Waters Xevo TQ-S Cronos mass spectrometer were as follows: source temperature 150 °C, desolvation temperature 350 °C, desolvation gas flow rate 600 L h^−1^, cone gas flow 100 L h^−1^, capillary potential 3.00 kV and cone potential 30 V. Nitrogen was used for both nebulizing and drying gas. The data were obtained in a scan mode, ranging from 50 to 1000 *m*/*z* in time 0.5 s intervals. Data acquisition software was MassLynx V 4.2 (Waters). Data interpretation software was Spectrus Processor 1.3 (ACDLabs 2019). Peak purity was evaluated from total absorbance chromatograms (TACs), recoded at a 200–500 nm range.

### 3.6. Synthetic Procedures

The synthesis of substrates: hydrazides and the polycyclic anhydride.

Hydrazides are usually synthesized through hydrazinolysis of respective esters [18] or reacting acyl chlorides with excess hydrazine. The hydrazinolysis method is efficient and high yielding but requires a long reaction time or elevated temperature. In the acyl chloride case, the high reactivity of the electrophile facilitates the formation of unwanted bis-acyl hydrazides. Therefore, to synthesize hydrazides **1a**–**e**, we decided to use a recently demonstrated protocol employing the carbonyldiimidazole (CDI) activation of the respective carboxylic acids [24,25,26]. Phenylhydrazine **1f** was converted to a free base form from commercially available hydrochloride. Sulfonyl hydrazide **1g** was synthesized by reacting benzenesulfonyl chloride with excess hydrazine hydrate, according to a procedure from the literature [27]. The experimental procedures, together with physicochemical data for **1a**–**g,** are presented in the Supporting Information (Section S1). Maleic anhydride is available commercially and the polycyclic anhydride for tecovirimat synthesis, (4-oxatetracyclo[5.3.2.0^2,6^.0^8,10^]dodec-11-ene-3,5-dione, in Figure 1A) was synthesized by using a method from the literature [28].

#### 3.6.1. General Procedure for Synthesis of Hydrazides **1a**–**e** and **1g**

Carboxylic acid was dissolved or suspended in anhydrous dichloromethane at room temperature and carbonyldiimidazole was added (1.2 eq.). The resulting mixture was stirred for one hour at room temperature and then added dropwise over 1h to hydrazine hydrate (10 eq.) solution in dichloromethane. After the addition was completed, the stirring continued overnight. After that time, the reaction mixture was diluted with dichloromethane (5×), washed twice with 5% NaHCO_3_ and once with water, dried over anhydrous Na_2_SO_4_ and evaporated under reduced pressure to yield the hydrazide, which did not require further purification.

#### 3.6.2. General Procedure for Synthesis of Isomaleimides **2a**–**2h** and Isosuccinimide **4**

Hydrazides **1a**–**1h** were dissolved in CH_2_Cl_2_. Then, K_2_CO_3_ (2 eq.) for **2b** or K_2_CO_3_/TEA (1.5 eq./1 eq.) for **2a**, **2c**–**2h** and **4** were added. Maleic anhydride (1 eq., for **2a**–**2h**) or 4a,5,5a,6-tetrahydro-1H-4,6-ethenocyclopropa[f]isobenzofuran-1,3(4H)-dione (1 eq., for **4**) was dissolved in CH_2_Cl_2_ and added dropwise over one hour at 0–5 °C. Stirring continued for one additional hour and then MsCl in CH_2_Cl_2_ (1 eq.) was added dropwise over another hour while still maintaining the temperature at 0–5 °C. The reaction mixture was left on a magnetic stirrer overnight, allowing it to gradually warm to room temperature. After this time, the reaction mixture was diluted with CH_2_Cl_2_ to twice its volume and washed with 5% NaHCO_3(aq)_ and distilled water. The organic layer was dried over Na_2_SO_4_ and evaporated under a reduced pressure with no heating (max bath temp. 25–30 °C) to yield the product that was used further (**2a**–**2e, 2h**) or purified on a chromatographic column, eluting system CH_2_Cl_2_/MeOH, 9:0.1 (**2f**). In the case of compound **2g**, workup was not performed due to the lack of desired reaction progress (numerous peaks in HPLC monitoring after stirring overnight).

#### 3.6.3. General Procedure for Synthesis of Maleimides **3a**–**3e**, **3h** and Tecovirimat (**5**)

The isomaleimides **2a**–**2f, 2h** were dissolved in anhydrous CH_2_Cl_2_. *N*-hydroxysuccinimide and imidazole were then added (10 mol% for **2a**, **2c**–**2e, 2h** and **5** or 30 mol% for **2b** and **2f**) and the reaction was carried out for 5–24 h at room temperature, monitored by HPLC. After completion of isomerization, the reaction mixture was diluted with CH_2_Cl_2_ five times and washed with 5% NaHCO_3(aq)_ and then distilled water. The organic layer was dried over Na_2_SO_4_ and evaporated until dry to obtain the crude products (**2a**–**2e**, **2h**, **5**). The final products were purified by means of column chromatography (chromatographic parameters for each compound given in p.3.7).

#### 3.6.4. Synthesis of Tecovirimat (**5**) from Maleimide **3a**

Maleimide **3a** (0.103 g), freshly prepared anhydrous toluene (5 mL) and cycloheptatriene (48 µL) were placed in a pressure-rated reaction tube and sealed with a fresh septum. The atmosphere in the tube was replaced with nitrogen using a Schlenk line and the tube was placed in a sand bath heated to 110 °C for 25 h. After that time, conversion of **3a** was complete (monitored by HPLC); the volatiles were evaporated and the residue was purified through automated flash chromatography, using the hexane/ethyl acetate eluent in gradient mode (from 9:1 to 4:6 *v*/*v*, over 39 min, at 13 mL/min flow rate).

### 3.7. Spectral and Analytical Data

Spectral and analytical data for hydrazides **1a**–**1h** are presented in Supporting Information (Section S1).

#### 3.7.1. N′-(5-oxofuran-2(5H)-ylidene)-4-(trifluoromethyl)benzohydrazide (**2a**)

White solid; 216 mg (74% yield); C_12_H_7_F_3_N_2_O_3_; MW 284.19; ^1^H NMR (500 MHz, DMSO-*d*_6_) δ (ppm) 6.92 (d, *J =* 5.30 Hz), 7.83 (d, *J* = 8.23 Hz, 2H, Ar-H), 7.89–7.96 (m, 1H, =CH-C=O), 7.97–8.06 (m, 2H, Ar-H), 11.91 (br. s., 1H, NH); ^13^C NMR (126 MHz, DMSO-*d*_6_) δ (ppm) 124.39 (q, *J* = 271.70 Hz), 125.65,126.60, 129.90, 131.47–132.75 (m), 137.06, 142.84, 147.39, 164.21, 166.70; ^19^F NMR (471 MHz, DMSO-*d*_6_) δ (ppm) −61.4 (br. s, 3F); HPLC 99.6% (MaxPlot, 200–800 nm), t_R_ = 1.89 min; UPLC-MS t_R_ = 6.08 min, Monoisotopic Mass 284.04, [M-H]^−^ =283.1, 94.48% (TAC, 200–500 nm).

#### 3.7.2. Tert-butyl 2-(5-oxofuran-2(5H)-ylidene)hydrazine-1-carboxylate (**2b**)

Beige solid; 2196 mg (74% yield); C_9_H_12_N_2_O_4_; MW 212.21; ^1^H NMR (500 MHz, DMSO-*d*_6_) δ (ppm) 1.40 (s, 9H, -(CH_3_)_3_), 6.75 (d, *J* = 5.6 Hz, 1H, CH-C=N), 7.79 (d, *J* = 5.6 Hz, 1H, CH-C=O), 10.78–10.86 (m, 1H, NH); ^13^C NMR (126 MHz, DMSO-*d*_6_) δ ppm 28.4 (s, 1C, (CH_3_)_3_), 81.0 (s, 1C, -O-C-(CH_3_)_3_), 125.0 (s, 1C, =CH-C=O), 142.5 (s, 1C, =CH-C=N), 144.0 (s, 1C, C=N), 152.8 (s, 1C, -NH-C=O), 167.0 (s, 1C, C=O); HPLC 93.86% (MaxPlot, 200–800 nm), t_R_ = 1.37 min; UPLC-MS t_R_ = 5.12 min, Monoisotopic Mass 212.08, [M+H]^+^ not present, [C_4_H_5_N_2_O_2_]^+^ = 113.0 (N-aminomaleimide cation), 98.5% (TAC, 200–500 nm). CAS 1565737-03-2 [29].

#### 3.7.3. N′-(5-oxofuran-2(5H)-ylidene)benzohydrazide (**2c**)

White solid; 303 mg (70% yield); C_11_H_8_N_2_O_3_; MW 216.20; ^1^H NMR (500 MHz, DMSO-*d*_6_) δ (ppm) 6.89 (d, *J* = 5.6 Hz, 1H, =CH-C=N), 7.43–7.49 (m, 2H, Ar-H(2,6)), 7.55 (d, *J* = 7.5 Hz, 1H, Ar-H(4)), 7.83 (d, *J* = 7.5 Hz, 2H, Ar-H(4,5)), 7.90 (d, *J* = 5.4 Hz, 1H, =CH-C=O), 11.67 (s, 1H, NH); ^13^C NMR (126 MHz, DMSO-*d*_6_) δ (ppm) 126.3 (s, 1C, =CH-C=O), 128.0–128.5 (m, 1C, Ar-C(4)), 128.8 (s, 2C, Ar-C(2,6)), 129.0, (br. s., 2C, Ar-C(3,5)), 132.4–132.8 (m, 1C, C(Ar)-C=O), 133.1–133.3 (m, 1C, =CH-C=N), 134.1–134.8 (m, 1C, C=N), 142.9 (s, 1C, -NH-C=O), 166.8 (s, 1C, -O-C=O); HPLC 92.57% (MaxPlot, 200–800 nm), t_R_ = 1.20 min; UPLC-MS t_R_ = 4.48 min, Monoisotopic Mass 216.05, [M+H]^+^ = 217.1, 97.08% (TAC, 200–500 nm). CAS 1803569-78-9 [30].

#### 3.7.4. 4-(Dimethylamino)-N′-(5-oxofuran-2(5H)-ylidene)benzohydrazide (**2d**)

Yellow solid; 505 mg (58% yield); C_13_H_13_N_3_O_3_; MW 259.27; ^1^H NMR (500 MHz, DMSO-*d*_6_) δ (ppm) 2.95–2.98 (m, 6H, CH_3_(x2)), 6.70–6.74 (m, 2H, Ar-H(3,5)), 7.17–7.19 (m, 2H, >CH-CO-), 7.72–7.76 (m, 2H, Ar-H(1,6)), 10.54–10.58 (m, 1H, NH); ^13^C NMR (126 MHz, DMSO-*d*_6_) δ (ppm) 40.0 (s, 2C, (-CH_3_)_2_), 111.1 (s, 2C, Ar-C(3,5)), 119.0 (s, 1C, Ar-C1), 125.5 (s, 1C, >CH-), 130.6 (s, 2C, Ar-C(2,6)), 142.9 (s, 1C, >CH-), 144.9–146.0 (m, 1C, C=N), 153.3 (s, 1C, Ar-C4), 163.9–165.2 (m, 1C, CO-NH), 167.0 (s, 1C, C=O); HPLC 98.91% (MaxPlot, 200–800 nm), t_R_ = 1.39 min; UPLC-MS t_R_ = 5.25 min, Monoisotopic Mass 259.10, [M+H]^+^ = 260.0, 96.65% (TAC, 200–500 nm).

#### 3.7.5. 4-Methoxy-N′-(5-oxofuran-2(5H)-ylidene)benzohydrazide (**2e**)

White solid; 510 mg (63% yield); C_12_H_10_N_2_O_4_; MW 246.22; ^1^H NMR (500 MHz; CDCl_3_) δ (ppm) 3.86 (s; 3H; -OCH_3_); 6.53 (d; *J* = 5.6 Hz; 1H; =CH-C=N); 6.94–6.99 (m; 2H; Ar-H(3,5)), 7.55 (d; *J* = 5.4 Hz; 1H; =CH-C=O); 7.84 (d; *J* = 8.8 Hz; 2H; Ar-H(2,6)), 9.38–9.51 (m; 1H; NH); ^13^C NMR (126 MHz; CDCl_3_) δ (ppm) 54.4–56.6 (m; 1C; OCH_3_); 114.3 (s; 2C; Ar-C(3,5)), 123.8–124.2 (s; 1C; Ar-C(1)), 125.2 (s; 2C; Ar-C(2,6)), 129.4 (s; 1C; =CH-C=O); 129.8 (br. s.; 1C; =CH-C=N); 129.9 (br. s.; 1C; C=N); 141.3 (s; 1C; -NH-C=O); 163.4 (s; 1C; Ar-C(4)), 164.7 (s; 1C; -O-C=O); HPLC 91.24% (MaxPlot, 200–800 nm), t_R_ = 1.29 min; UPLC-MS t_R_ = 4.81 min; Monoisotopic Mass 246.06; [M+H]^+^ = 247.1; 85.70% (TAC, 200–500 nm). CAS 1803569-80-3 [30].

#### 3.7.6. 5-(2-Phenylhydrazineylidene)furan-2(5H)-one (**2f**)

Yellow solid; 610 mg (35% yield; purification by column chromatography, eluting system CH_2_CL_2_/MeOH, 9:0.1); C_10_H_8_N_2_O_2_; MW 188.19; ^1^H NMR (500 MHz, CDCl_3_) δ (ppm) 6.25–6.33 (m, 1 H, >CH-), 6.94–7.01 (m, 1 H, Ar-H4), 7.10–7.17 (m, 2 H, Ar-H2,6), 7.27–7.34 (m, 2 H, Ar-H3,5), 7.36–7.41 (m, 1 H, >CH-), 8.17–8.32 (m, 1 H, NH); ^13^C NMR (126 MHz, CDCl_3_) δ (ppm) 113.4 (s, 2C, Ar-C(2,6)), 120.1 (s, 1C, Ar-C(4)), 122.3 (s, 1C, CH-C=N), 129.6 (s, 2C, Ar-C(3,5)), 139.8 (s, 1C, CH-C=N), 141.4 (s, 1C, Ar-C(1)), 142.5 (s, 1C, C=N), 166.4 (s, 1C, C=O); HPLC 99.64% (MaxPlot, 200–800 nm), t_R_ = 1.77 min; UPLC-MS t_R_ = 6.76 min, Monoisotopic Mass 188.06, [M+H]^+^ = 189.2, 98.73% (TAC, 200–500 nm). CAS 52726-95-1 [19].

#### 3.7.7. N′-[5-oxo-2,5-dihydrofuran-2-ylidene]thiophene-2-carbohydrazide (**2h**)

Yellow solid; 365 mg (63%); ^1^H NMR (500 MHz, CHLOROFORM-*d*) d ppm 6.54 (d, *J* = 5.59 Hz, 1 H) 7.12–7.16 (m, 1 H) 7.52 (br. s., 1 H) 7.61–7.66 (m, 1 H) 7.68–8.13 (m, 1 H) 9.39 (br. s, 1 H); HPLC 98.83% (MaxPlot, 200–800 nm), t_R_ = 1.16 min; UPLC-MS t_R_= 4.43 min; Monoisotopic Mass 246.06; [M+H]^+^ = 223.0; 98.02% (TAC, 200–500 nm).

#### 3.7.8. N-(2,5-dioxo-2,5-dihydro-1H-pyrrol-1-yl)-4-(trifluoromethyl)benzamide (**3a**)

White solid; 207 mg (93% yield; purification by extraction); C_12_H_7_F_3_N_2_O_3_; MW 284.19; ^1^H NMR (500 MHz, DMSO-*d*_6_) δ (ppm) 7.22–7.26 (m, 2H, 2x = CH-C=O), 7.92 (d, *J* = 8.3 Hz, 2H, Ar-H), 8.08 (d, *J* = 8.2 Hz, 2H, Ar-H), 11.32 (s, 1H, NH); ^13^C NMR (126 MHz, DMSO-*d*_6_) δ (ppm) 124.3 (q, *J* = 272.0 Hz, 1C, CF_3_), 126.4 (q, *J* = 3.6 Hz, 2C, Ar-C(3,5)), 129.2 (s, 2C, Ar-C(2,6)), 133.0 (q, *J* = 32.1 Hz, 1C, Ar-C4), 134.5 (s, 2C, 2x > CH-), 134.9 (s, 1C, Ar-C1), 164.9 (s, 1C, Ar-C=O), 168.6 (s, 2C, 2x N-C=O); ^19^F NMR (471 MHz, DMSO-*d*_6_) δ (ppm) −61.48 (br. s., 3F); HPLC 100% (MaxPlot, 200–800 nm), t_R_ = 1.77 min; UPLC-MS, t_R_ = 6.08 min, Monoisotopic Mass 284.04, [M-H]^−^ = 283.0, 95.38% (TAC, 200–500 nm). CAS 1565736-80-2 [29,31].

#### 3.7.9. Tert-butyl (2,5-dioxo-2,5-dihydro-1H-pyrrol-1-yl)carbamate (**3b**)

White solid; 111 mg (20% yield; purification by column chromatography, eluting system AcOEt/Petroleum ether, 1:1; yield before purification = 48%, 248 mg); C_9_H_12_N_2_O_4_; MW 212.21; ^1^H NMR (500 MHz, DMSO-*d*_6_) δ (ppm) 1.35–1.40 (m, 9H, CH_3_), 7.11 (s, 2H, >CH-), 9.57 (s, 1H, NH), [before purification by column chromatography: ^1^H NMR (500 MHz, DMSO-*d*_6_) δ (ppm) 1.38 (s, 9H, CH_3_) 7.11 (s, 2H, >CH-) 9.57 (s, 1H, NH)]; ^13^C NMR (126 MHz, DMSO-*d*_6_) δ (ppm) 28.4 (s, 3C, CH_3_), 81.5 (s, 1C, -O-C-(CH_3_)_3_), 134.2 (s, 2C, >CH-), 154.4 (s, 2C, -CO-N), 168.9 (s, 1C, -CO-O-); HPLC 93.63% (MaxPlot, 200–800 nm), t_R_ = 1.30 min; UPLC-MS t_R_ = 4.87 min, Monoisotopic Mass 212.08, [M+H]^+^ not present, [C_4_H_5_N_2_O_2_]^+^ = 113.1, N-aminomaleimide cation 96.80% (TAC, 200–500 nm). CAS 1565736-82-4 [29].

#### 3.7.10. N-(2,5-dioxo-2,5-dihydro-1H-pyrrol-1-yl)benzamide (**3c**)

White solid; 142 mg (62% yield; purification by column chromatography, eluting system AcOEt/Petroleum ether, 2:1); C_11_H_8_N_2_O_3_; MW 216.20; ^1^H NMR (500 MHz, DMSO-*d*_6_) δ (ppm) 7.22 (s, 2H, 2x = CH-C=O), 7.50–7.55 (m, 2H, Ar-H(3,5)), 7.60–7.64 (m, 1H, Ar-H(4)), 7.88 (dd, *J* = 8.3, 1.04 Hz, 2H, Ar-H(2,6)), 11.04 (s, 1H, NH); ^13^C NMR (126 MHz, DMSO-*d*_6_) δ (ppm) 128.2 (s, 2C, Ar-C(2,6)), 129.3 (s, 2C, Ar-C(3,5)), 131.2 (s, 1C, Ar-C(1)), 133.3 (s, 1C, Ar-C(4)), 134.5 (s, 2C, 2x =CH-C=O), 165.9 (s, 1C, Ar-C=O), 168.9 (s, 2C, 2x N-C=O); HPLC 96.47% (MaxPlot, 200–800 nm), t_R_ = 1.14 min; UPLC-MS t_R_ = 4.20 min, Monoisotopic Mass 216.05, [M+H]^+^ = 217.1, 95.22% (TAC, 200–500 nm). CAS 1843230-38-5 [31].

#### 3.7.11. 4-(Dimethylamino)-N-(2,5-dioxo-2,5-dihydro-1H-pyrrol-1-yl)benzamide (**3d**)

Yellow solid; 230 mg (51% yield; purification by column chromatography, eluting system AcOEt/Petroleum ether, gradient 2:1–4:1); C_13_H_13_N_3_O_3_; MW 259.27; ^1^H NMR (500 MHz, DMSO-*d*_6_) δ (ppm) 2.95–2.98 (m, 6H, CH_3_(x2)), 6.70–6.74 (m, 2H, Ar-H(3,5)), 7.17–7.19 (m, 2H, >CH-CO-), 7.72–7.76 (m, 2H, Ar-H(1,6)), 10.54–10.58 (m, 1H, NH); ^13^C NMR (126 MHz, DMSO-*d*_6_) δ (ppm) 40.0 (s, 2C, (-CH_3_)_2_), 111.4 (s, 2C, Ar-C(3,5)), 117.2 (s, 1C, Ar-C1), 129.8 (s, 2C, Ar-C(2,6)), 134.4 (s, 2C, -CHx2), 153.5 (s, 1C, Ar-C6), 165.6 (s, 2C, CO-N-NH), 169.3 (s, 1C, Ar-CO); HPLC 99.39% (MaxPlot, 200–800 nm), t_R_ = 1.40 min; UPLC-MS t_R_ = 4.92 min, Monoisotopic Mass 259.10, [M+H]^+^ = 260.2, 97.84% (TAC, 200–500 nm).

#### 3.7.12. N-(2,5-dioxo-2,5-dihydro-1H-pyrrol-1-yl)-4-methoxybenzamide (**3e**)

White solid; 165 mg (60% yield; purification by column chromatography, eluting system AcOEt/Petroleum ether, 3:2); C_12_H_10_N_2_O_4_; MW 246.22; ^1^H NMR (500 MHz, DMSO-*d*_6_) δ (ppm) 3.81 (s, 3H, CH_3_), 7.01–7.08 (m, 2H, Ar-H), 7.20 (s, 2H, >CH-), 7.83–7.90 (m, 2H, Ar-H), 10.86 (s, 1H, NH); ^13^C NMR (126 MHz, DMSO-*d*_6_) δ (ppm) 56.0 (s, 1C, -OCH_3_), 114.6 (s, 2C, Ar-C3,5), 123.3 (s, 1C, Ar-C1), 130.3 (s, 2C, Ar-C2,6), 134.4 (s, 2C, >CH-), 163.2 (s, 2C, -N-C=O), 165.3 (s, 1C, Ar-C4), 169.0 (s, 1C, Ar-C=O); HPLC 94.59% (MaxPlot, 200–800 nm), t_R_ = 1.22 min; UPLC-MS t_R_ = 4.51 min, Monoisotopic Mass 246.06, [M+H]^+^ = 247.1, 95.46% (TAC, 200–500 nm).

#### 3.7.13. N-(2,5-dioxo-2,5-dihydro-1H-pyrrol-1-yl)thiophene-2-carboxamide (**3h**)

White solid; 127 mg (60% yield; purification by automated flash chromatography, eluting system: CH_2_Cl_2_/Methanol in gradient mode, 0–10% *v*/*v* Methanol over 13 min); C_9_H_6_N_2_O_3_S; MW 222.22; ^1^H NMR (500 MHz, DMSO-*d*_6_) δ ppm 7.21 (s, 2 H) 7.22 (dd, *J* = 5.01, 3.80 Hz, 1 H) 7.90 (dd, *J* = 3.76, 1.11 Hz, 1 H) 7.91–7.94 (m, 1 H) 11.07 (s, 1 H); ^13^C NMR (126 MHz, DMSO-*d*_6_) δ ppm 129.04 131.00 133.68 134.44 135.52 160.81 168.81; HPLC 94.89%, t_R_ = 1.05 min; UPLC-MS t_R_ = 3.86 min, Monoisotopic mass 222.01; [M-1]^−^ = 221.02, 86.97% (TAC, 200–500 nm).

#### 3.7.14. (Z)-N’-(3-oxo-3,3a,4,4a,5,5a,6,6a-octahydro-1H-4,6-ethenocyclopropa[f]isobenzofuran-1-ylidene)-4-(trifluoromethyl)benzohydrazide (4)

Green solid; 277 mg (71% yield); C_19_H_13_F_3_N_2_O_3_; MW 376.34; ^1^H NMR (500 MHz, CDCl_3_) δ (ppm) mixture of rotamers: 0.22–0.26, 0.28–0.38 (m, m, 2H), 0.80–0.84, 1.07–1.15 (m, m, 2H), 3.13, 3.22–3.24 (br.m, m, 2H), 3.41–3.47 (m, 2H), 5.82, 5.87 (br. m, dd, *J* = 4.8, 3.3 Hz, 2H), 7.64 (d, *J* = 8.2 Hz, 2H), 7.87 (d, *J* = 8.0 Hz, 2H), 8.45 (br. s., 1H); ^13^C NMR (126 MHz, CDCl_3_) δ (ppm) mixture of rotamers (signals corresponding to the less abundant rotamer are given in parentheses): 1.1, 4.5, 5.2, 9.5, 9.7, (29.4), 29.8, 33.5, 33.6, 43.9, (44.3), 45.9, 123.5 (q, *J* = 273 Hz), 125.8, 125.8, 127.9, (128.1), 128.2, (128.4), 128.5, 133.9, 163.8, 172.5, 175.0; UPLC-MS t_R_ = 6.86 min, Monoisotopic Mass 276.10, [M+H]^+^ = 377.2, 65.28% (TAC, 200–500 nm).

#### 3.7.15. (Z)-N′-(3-oxo-3,3a,4,4a,5,5a,6,6a-octahydro-1H-4,6-ethenocyclopropa[f]isobenzofuran-1-ylidene)-4-(trifluoromethyl)benzohydrazide (Tecovirimat, **5**)

White solid; 101 mg/76 mg (46% yield, starting from 4; 56% yield starting from 3a; purified through automated flash chromatography, described in p. 4.3); C_19_H_13_F_3_N_2_O_3_; MW 376.34; ^1^H NMR (500 MHz, DMSO-*d*_6_) δ (ppm) −0.02–0.08 (m, 1H, -CHH-), 0.18–0.27 (m, 1H, -CHH-), 1.10–1.15 (m, 2H, >CH-CH<), 3.21–3.29 (m, 4H, 2x > CH-CH=, 2x > CH-CO), 5.69–5.80 (m, 2H, -CH=CH-), 7.86–7.93 (m, 2H, 2x Ar-H), 7.99–8.08 (m, 2H, 2x Ar-H), 11.32–11.38 (1H, NH); ^13^C NMR (126 MHz, DMSO-*d*_6_) δ (ppm) 9.7 (>CH-CH<), 14.6 (>CH-CH_2_-CH<), 33.6 (2x > CH-C=), 43.5 (2x > CH-C=O), 123.2 (-CF_3_), 125.4 (Ar-C(3,5)), 126.3 (Ar-C(2,6)), 127.8 (Ar-C4), 128.1 (Ar-C1), 129.2 (-CH=CH-), 164.1 (-NH-C=O), 175.3 (2x C=O); ^19^F NMR (471 MHz, DMSO-*d*_6_) δ (ppm) −61.6 (s, 3F); UPLC-MS t_R_ = 7.10 min, Monoisotopic Mass 276.10, [M+H]^+^ = 377.1, 95.23% (TAC, 200–500 nm). CAS 869572-92-9.

### 3.8. Computational Procedure

For the computational studies, we used a two-step Molecular Mechanics + SemiEmpirical (MM+SE) protocol: a shortened version of a three-step conformation generation procedure that we used earlier for the NMR chemical shift prediction studies [32,33,34]. First, we generated conformation sets by using a genetic algorithm and MMFF94 forcefield, as implemented in the Balloon program [35]. We used 1000 starting conformations and 10,000,000 GA generations and set the target conformation number to 10. The algorithm produced 1–9 conformations for isomaleimides **2a**–**f, 2h** and 43 conformations for isosuccinimide **4**. These conformations were optimized by using the PM7 semiempirical quantum chemistry method [23] (MOPAC2016 [36]) and the COSMO solvent model. We used a dielectric constant for dichloromethane (ε = 8.93) and a 2.5 Angstrom probe radius. For the lowest-ΔH_f_ conformations (Coordinates in Tables S1-S8, Supplementary materials), we generated molecular orbitals and .mgf files for inspection with the Jmol 16.3.27 program [37]. Selected LUMO orbitals are plotted in Figure 7 and all HOMO and LUMO, as well as few selected other orbitals, are shown in Figures S1–S23 (Supplementary Materials).

## 4. Conclusions

In this study, we have demonstrated that hydrazide-based isomaleimides easily rearrange to maleimides at room temperature by using catalytic amounts of NHS and imidazole. Thus, we described a new laboratory-scale synthetic pathway towards the maleimide intermediates of the antiviral drug tecovirimat and showed for the first time that tecovirimat can be obtained by using an organocatalytic isoimide rearrangement.

Encompassing the isomaleimide synthesis and rearrangement to maleimide, we obtained intermediates **3a** and **3b** from hydrazides in two steps, in higher yields than the single-step syntheses described in the patent literature (68% vs. 54% for **3a** and 37% vs. 18% for **3b**), without the need to use high reaction temperatures, with the potential to reduce the use of chromatography. For compound **3a**, we performed tecovirimat synthesis according to the patent literature and demonstrated that an improved yield of tecovirimat synthesis can be achieved (38% vs. 35%).

Additionally, we found this approach useful in the synthesis of the isomaleimide and maleimide derivatives of several benzohydrazides and tiophenecarbonyl hydrazide, regardless of the activation or deactivation of the aromatic ring. We also found that isosuccinimides also rearrange to succinimides under the same conditions. We did this by synthesizing tecovirimat from isosuccinimide **4** with 10 mol% NHS and imidazole at room temperature.

Preliminary computational studies on isomaleimide derivatives indicated that the charge distribution of the hydrazide proton (and thus its acidity) has some correlation with the isomerization ability.

## Figures and Tables

**Table 1:** Optimization of synthetic conditions for isomaleimide **2a**. Reactions were performed on a 100 mg scale at 0 °C in CH_2_Cl_2_ overnight, excluding the first entry, which was performed at 0 °C in ethyl acetate in 15 min. Conditions taken from [11].

No	Base	MsCl	Solvent	Isolated Yield (Crude)	HPLC Purity
1	TEA (3 eq.)	3 eq.	EtOAc	86%	80.5%
2	TEA (3 eq.)	1.05 eq.	CH_2_Cl_2_	64%	3%
3	TEA (2 eq.)			62%	78%
**4**	**TEA (1 eq.) + K_2_CO_3_ (1.5 eq.)**			**59%**	**93.9%**
5	TEA (1 eq.) + K_2_CO_3_ (1 eq.)			47%	95%
6	TEA (0.5 eq.) + K_2_CO_3_ (1.5 eq.)			36%	94.3%
7	K_2_CO_3_ (2 eq.)			9%	22%

**Table 2:** Catalyst screening for isomaleimide **2a** to maleimide **3a** rearrangement. Reaction conditions: 5 mg scale, anhydrous CH_2_Cl_2_, room temperature, 5 mol% of each catalyst. Isomerization progress was estimated with ^1^H NMR, based on double-bond peak areas.

No	Catalyst	Isomerization Progress (^1^H NMR) ^a^
1	NMM	-
2	Imidazole	50%
3	NHS	92%
**4**	**Imidazole + NHS**	**99%**
5	NMM + NHS	80%
6	TEA + NHS	80%

^a 1^H NMR signals from double-bond protons were followed (1H doublet at 6.92 ppm for **2a** and 2H singlet at 7.22 ppm for **3a**).

**Table 3:** Net charges for selected atoms and LUMO energies in isomaleimides **2a**–**f**, **2h** and **4**. For convenience, experimentally observed isomerization tendency is marked.

Atom	2a	2b	2c	2d	2e	2f	2h	4
-O-**C**=O	0.62	0.61	0.61	0.62	0.62	0.61	0.60	0.62
=**C**-C=O	−0.25	−0.22	−0.23	−0.27	−0.26	−0.20	−0.21	−0.22 ^a^
N=C-**C**=	−0.07	−0.08	−0.10	−0.06	−0.06	−0.14	−0.12	−0.19 ^a^
O-**C**=N	0.30	0.30	0.24	0.27	0.28	0.30	0.32	0.40
N-**H**	0.37	0.37	0.34	0.37	0.37	0.30	0.37	0.36
LUMO energy [eV]	−1.678	−1.678	−1.724	−1.516	−1.574	−1.704	−1.785	−1.142 ^b^
isomerization	+++	+	+++	++	+++	-	+++	++

^a^ sp^3^ carbon atoms without double bond. ^b^ The LUMO orbital in **4** had a different distribution; it did not cover the isoimide fragment.

## Data Availability

The original contributions presented in this study are included in the article and the Supplementary Materials. Further inquiries can be directed to the corresponding author.

## Supplementary Materials

The following supporting information can be downloaded at: https://www.mdpi.com/article/10.3390/ijms27010061/s1.
