# Supplementary material for: Synthesis of Antiviral Drug Tecovirimat and Its Key Maleimide Intermediates Using Organocatalytic Mumm Rearrangement at Ambient Conditions
Source: Int J Mol Sci. 2025 Dec 20;27(1):61. doi: 10.3390/ijms27010061 (PMC12785354; doi:10.3390/ijms27010061)
Supplement: Supplementary file 1 [file ijms-27-00061-s001.zip › ijms-4031305-supplementary.pdf]

**Electronic supplementary Materials for the article**

# Synthesis of antiviral drug tecovirimat and its key maleimide intermediates using organocatalytic Mumm rearrangement at ambient conditions

Przemysław Szafrański[1*], Wojciech Trybała[1], Adam Mazur, Katarzyna Pańczyk-Straszak[1], Alicja Kacprzak, Vittorio Canale[1], Paweł Zajdel[1]

[1] Department of Organic Chemistry, Faculty of Pharmacy, Jagiellonian University Medical College, 9 Medyczna Street, 30-688 Kraków, Poland

**Contents**

**S1. Synthetic and physicochemical data for hydrazides 1a-1h,** pp. 2-4

**S2. NMR spectra and UPLC/MS datasets,** pp. 5-61

**S3. Computational results,** pp. 62-78

# 1. SYNTHETIC PROCEDURES AND PHYSICOCHEMICAL DATA FOR HYDRAZIDES 1a-1h

## 1.1. 4-trifluoromethylbenzohydrazide (1a)

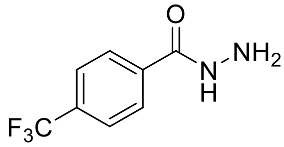

White solid; 783 mg, (72.6% yield); $C_8H_7F_3N_2O$; MW 204.15; $^1$H NMR (500 MHz, $CDCl_3$) δ (ppm) 4.12 (br. s., 2H, $-NH_2$), 7.68 (d, *J*=8.2 Hz, 2H, Ar-H), 7.86 (d, *J*=8.1 Hz, 2H, Ar-H), 7.93 (br. s., 1H, -NH-); HPLC 99.6%, $t_R$=1.23 min.

## 1.2. *Tert*-butyl hydrazinecarboxylate (1b)

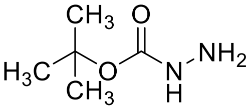

Hydrazine monohydrate (3.44 g, 0.0687 mole, 3 eq.) was dissolved in isopropanol (13 mL) in a 100 mL round-bottom flask. Di-tert-butyl dicarbonate (5 g, 0.0229 mole, 1 eq.) in isopropanol (5 mL) was added dropwise to the prepared solution. After the entire volume was added, the reaction mixture was left at room temperature on a magnetic stirrer for 2 hours. After this time, the reaction mixture was evaporated to dryness. The residue was dissolved in $CH_2Cl_2$ and dried over $MgSO_{4(anh)}$. After filtration and distillation of the solvent, the crude product was obtained and used in further stages of the synthesis.

White solid; 2400 mg (79% yield); $C_5H_{12}N_2O_2$; MW 132.16; $^1$H NMR (500 MHz, $CDCl_3$) δ (ppm) 1.36 - 1.5 (s, 9H, $(-CH_3)_3$), 3.17 - 3.48 (br. s., 2H, $-NH_2$), 5.70 - 5.98 (br. s., 1H, -NH-).

## 1.3. Benzohydrazide (1c)

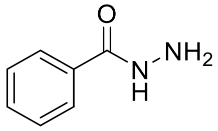

White solid; 760 mg (68% yield); $C_7H_8N_2O$; MW 136.15; $^1$H NMR (500 MHz, $CDCl_3$) δ (ppm) 3.40 (br. s., 2H, $NH_2$) 7.40 - 7.47 (m, 2H, Ar-H) 7.48 - 7.54 (m, 1H, Ar-H) 7.70 - 7.77 (m, 2H, Ar-H).

## 1.4. 4-(Dimethylamino)benzohydrazide (1d)

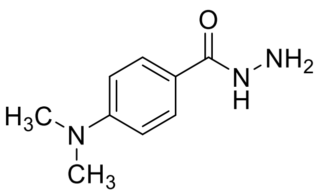

White solid; 640 mg (59% yield); $C_9H_{13}N_3O$; MW 179.22; $^1$H NMR (500 MHz, DMSO-$d_3$) δ (ppm) 2.86 - 2.96 (m, 6H, $(CH_3)_2$), 4.20 - 4.38 (m, 2H, $NH_2$), 6.58 - 6.70 (m, 2H, Ar-H), 7.58 - 7.70 (m, 2H, Ar-H), 9.25 - 9.43 (m, 1H, NH); HPLC 99.53%, $t_R$=0.853 min.

### 1.5. 4-Methoxybenzohydrazide (1e)

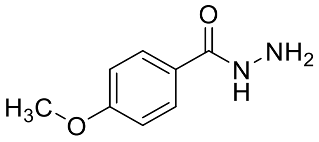


White solid; 680 mg (62% yield); $C_8H_{10}N_2O_2$; MW 166.18; $^1$H NMR (500 MHz, $CDCl_3$) δ (ppm) 3.83 - 3.84 (m, 3H, $-OCH_3$), 6.89 - 6.93 (m, 2H, Ar-H) 7.70 - 7.74 (m, 2H, Ar-H).

### 1.6. Phyenylhydrazine (1f)

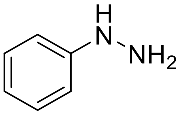


Phenylhydrazine hydrochloride (3 g, 0.0207 mole) was dissolved in a minimum volume of distilled water and treated with 40% NaOH(aq). The resulting base was extracted into $CH_2Cl_2$, dried over $Na_2SO_{4(anh)}$ and the solvent was distilled off. The crude product was used in isomaleimide synthesis.

Yellow oil; 1953 mg (87% yield); $C_6H_8N_2$; MW 108.14; $^1$H NMR (500 MHz, $CDCl_3$) δ (ppm) 3.00 - 4.13 (m, 2H, $NH_2$), 6.79 - 6.84 (m, 2H, Ar-H(3,5)), 7.21 - 7.27 (m, 3H, Ar-H(2,4,6)).

### 1.7. Benzenesulfonohydrazide (1g)

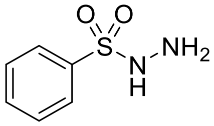


Sulfonyl chloride (1,77 g, 0.0100 mole, 1 eq.) was added dropwise to a stirring solution of hydrazine monohydrate in $CH_2Cl_2$ in a 100 mL round-bottom flask. After the entire volume was added, the reaction mixture was stirred for another 15 minutes at room temperature, and then alkalized with 10% $Na_2CO_{3(aq)}$ to pH=11. The reaction mixture was diluted with $CH_2Cl_2$. After phase separation, the organic layer was dried over $Na_2SO_{4(anh)}$ and the solvent was distilled off. The crude product was crystallized from EtOH and used in the next step of the synthesis.

White solid; 1498 mg (87% yield); $C_6H_8N_2O_2S$; MW 172.20; $^1$H NMR (500 MHz, DMSO-$d_6$) δ (ppm) 4.07 (d, $J$=2.3 Hz, 2H, $NH_2$), 7.53 - 7.59 (m, 2H, Ar-H(3,5)), 7.59 - 7.64 (m, 1H, Ar-H(4)), 7.74 - 7.79 (m, 2H, Ar-H(2,6)), 8.36 (br. s., 1H, NH); HPLC 99.33% (MaxPlot, 200-800 nm), $t_R$=0.861 min.

**1.8 thiophene-2-carbohydrazide 1h**

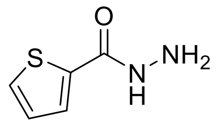

Tiophenecarbonyl chloride (1 g, 0.0068 mol, 1 eq) dissolved in $CH_2Cl_2$ (10 mL) was added dropwise over one hour to a mixture of hydrazine hydrate (4.5 mL, 13 equiv) and dichloromethane (10 mL), at 0°C. After the addition was completed, the stirring continued overnight. After that, the reaction mixture was diluted with $CH_2Cl_2$ (20 mL). The aqueous phase was separated and extracted with ethyl acetate (3x20 mL). Both organic phases were then extracted with 5% aqueous hydrochloric acid (3x20 mL). The combined acidic aqueous phase was neutralized with $NaHCO_3$ to slightly basic pH and extracted with ethyl acetate (4x15 mL). The combined organic phase was dried with anhydrous $Na_2SO_4$ and evaporated to yield the product, which was used without further purification.

White solid; 360 mg (37%), $^1$H NMR (500 MHz, DMSO-$d_6$) d ppm 4.42 (br. s., 2 H) 7.09 (dd, $J$=4.98, 3.76 Hz, 1 H) 7.67 (dd, $J$=3.72, 1.07 Hz, 1 H) 7.70 (dd, $J$=5.01, 1.07 Hz, 1 H) 9.72 (s, 1 H);

# 2. SPECTRA

## 2.1. 4-(Trifluoromethyl)benzohydrazide (1a)

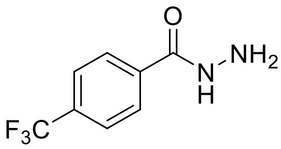

### 2.1.1. $^{1}$H NMR

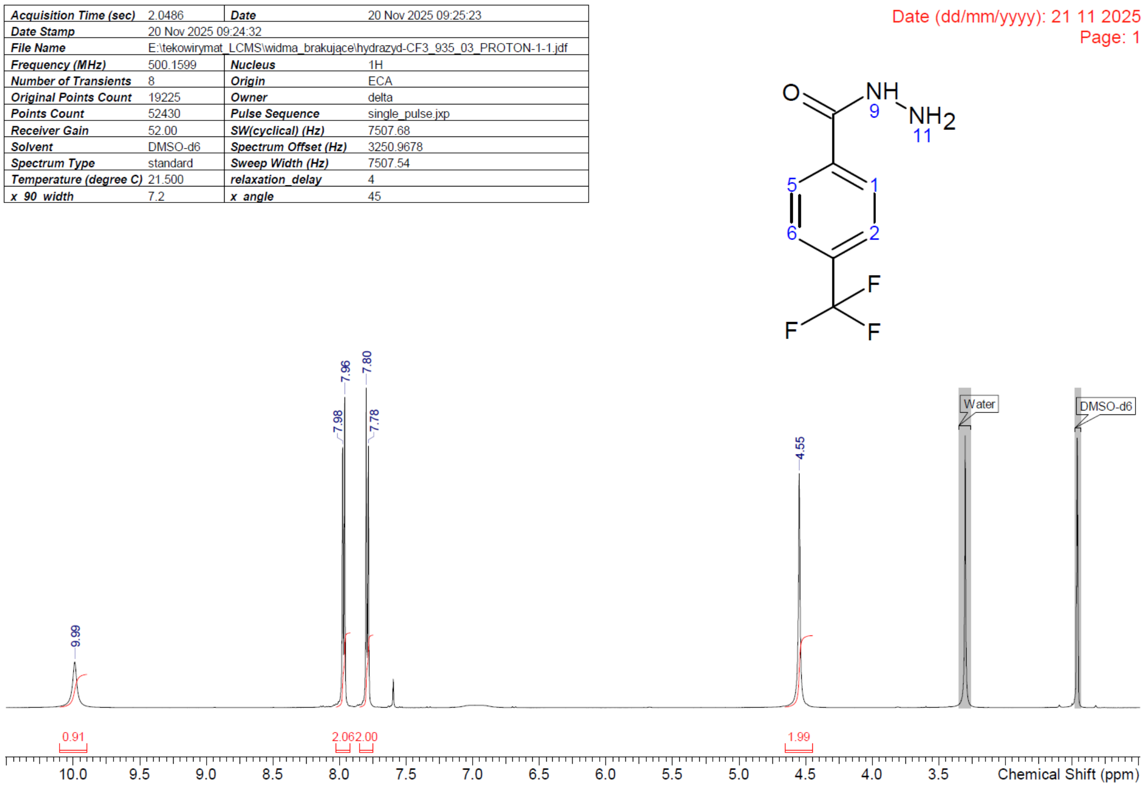

| | | | |
|---|---|---|---|
| Acquisition Time (sec) | 2.0486 | Date | 20 Nov 2025 09:25:23 |
| Date Stamp | 20 Nov 2025 09:24:32 | | |
| File Name | E:\tekowirymat_LCMS\widma_brakujące\hydrazyd-CF3_935_03_PROTON-1-1.jdf | | |
| Frequency (MHz) | 500.1599 | Nucleus | 1H |
| Number of Transients | 8 | Origin | ECA |
| Original Points Count | 19225 | Owner | delta |
| Points Count | 52430 | Pulse Sequence | single_pulse.jxp |
| Receiver Gain | 52.00 | SW(cyclical) (Hz) | 7507.68 |
| Solvent | DMSO-d6 | Spectrum Offset (Hz) | 3250.9678 |
| Spectrum Type | standard | Sweep Width (Hz) | 7507.54 |
| Temperature (degree C) | 21.500 | relaxation_delay | 4 |
| x_90_width | 7.2 | x_angle | 45 |

## 2.2. *Tert*-butyl hydrazinecarboxylate (1b)

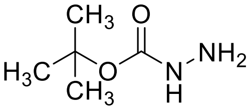

### 2.2.1. $^{1}$H NMR

| | | | |
|---|---|---|---|
| *Acquisition Time (sec)* | 4.3647 | *Date* | 23 Oct 2023 13:47:25 |
| *Date Stamp* | 23 Oct 2023 13:46:16 | | |
| *File Name* | \\AppServerM9\Wyniki_NMR_43_\2023\KPS23-1_935_43_PROTON-1-1.jdf | | |
| *Frequency (MHz)* | 500.1599 | *Nucleus* | 1H |
| *Number of Transients* | 8 | *Origin* | ECA |
| *Original Points Count* | 40960 | *Owner* | delta |
| *Points Count* | 104858 | *Pulse Sequence* | single_pulse.jxp |
| *SW(cyclical) (Hz)* | 7507.54 | *Solvent* | CHLOROFORM-d |
| *Spectrum Offset (Hz)* | 3251.0042 | *Spectrum Type* | standard |
| *Sweep Width (Hz)* | 7507.46 | *Temperature (degree C)* | 20.600 |

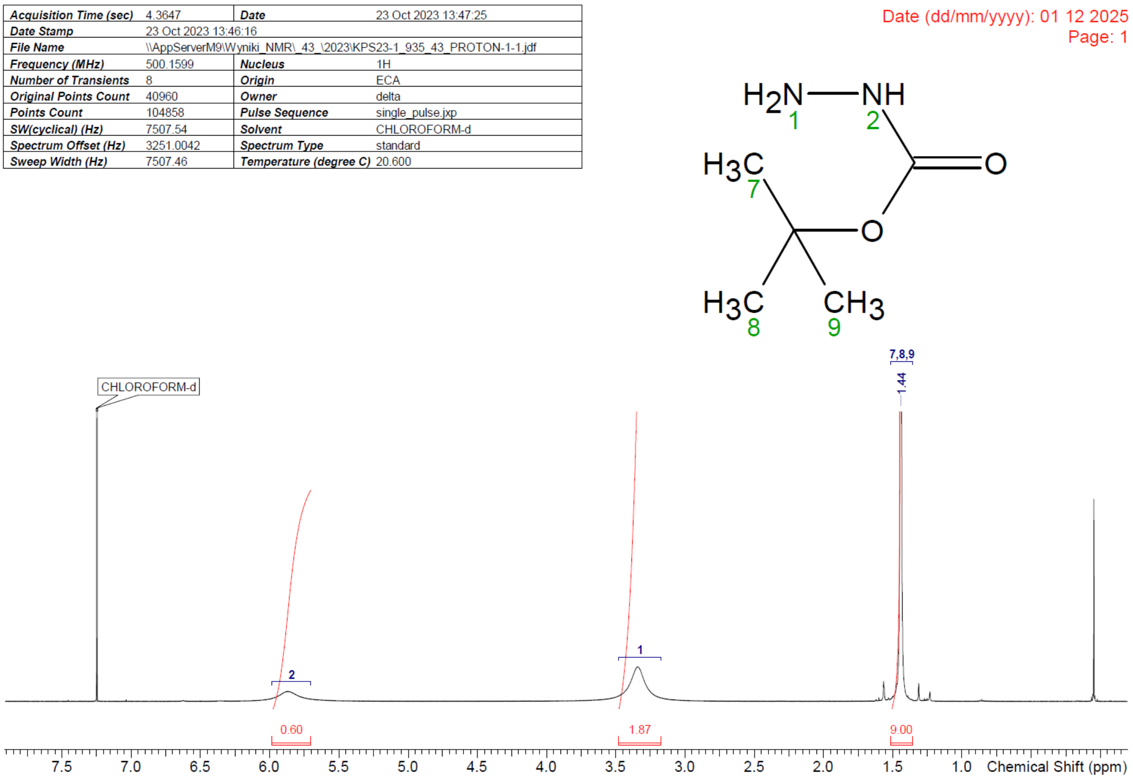

## 2.3. Benzohydrazide (1c)

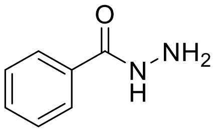


### 2.3.1. $^1$H NMR

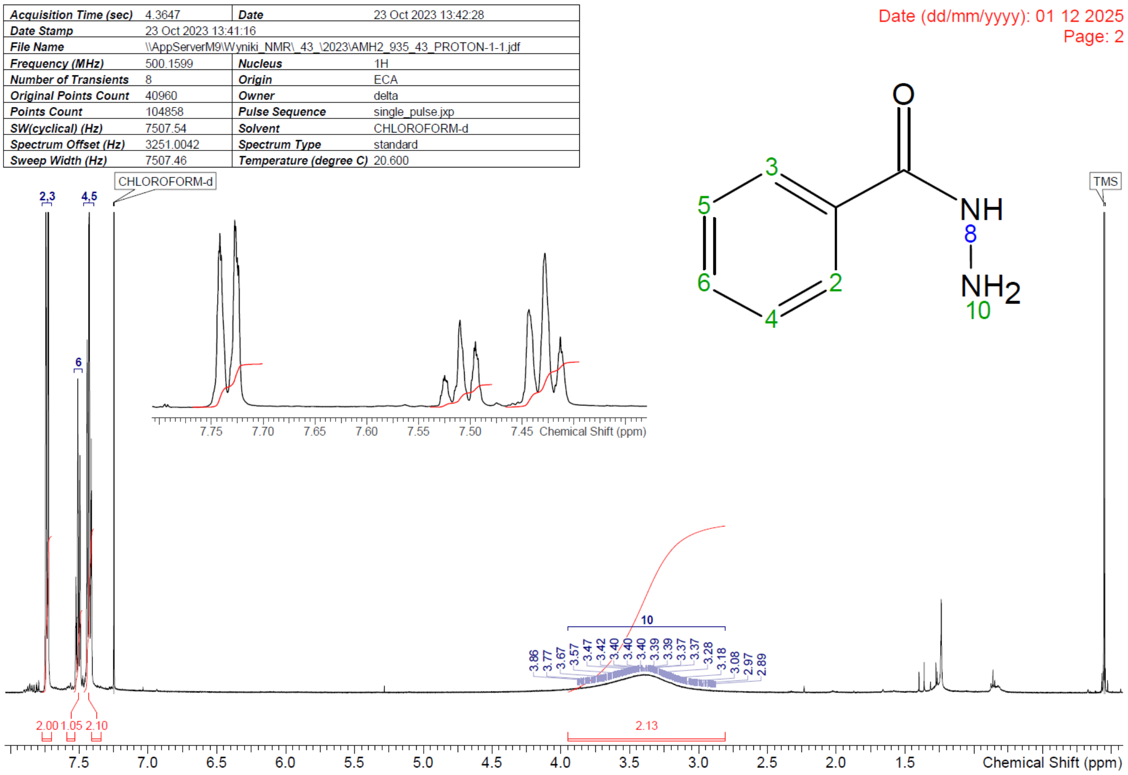

## 2.4. 4-(Dimethylamino)benzohydrazide (1d)

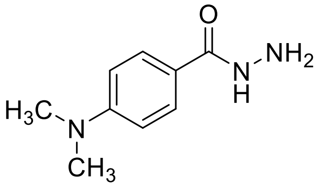

### 2.4.1. $^{1}$H NMR

| | | | |
|---|---|---|---|
| **Acquisition Time (sec)** | 4.3647 | **Date** | 18 Jan 2024 14:53:47 |
| **Date Stamp** | 18 Jan 2024 14:52:37 | | |
| **File Name** | \\AppServerM9\Wyniki_NMR_43_\2024\NCH32-hydrazyd_935_43_PROTON-1-1.jdf | | |
| **Frequency (MHz)** | 500.1599 | **Nucleus** | 1H |
| **Number of Transients** | 8 | **Origin** | ECA |
| **Original Points Count** | 40960 | **Owner** | delta |
| **Points Count** | 104858 | **Pulse Sequence** | single_pulse.jxp |
| **SW(cyclical) (Hz)** | 7507.54 | **Solvent** | DMSO-d6 |
| **Spectrum Offset (Hz)** | 3251.0042 | **Spectrum Type** | standard |
| **Sweep Width (Hz)** | 7507.46 | **Temperature (degree C)** | 21.400 |

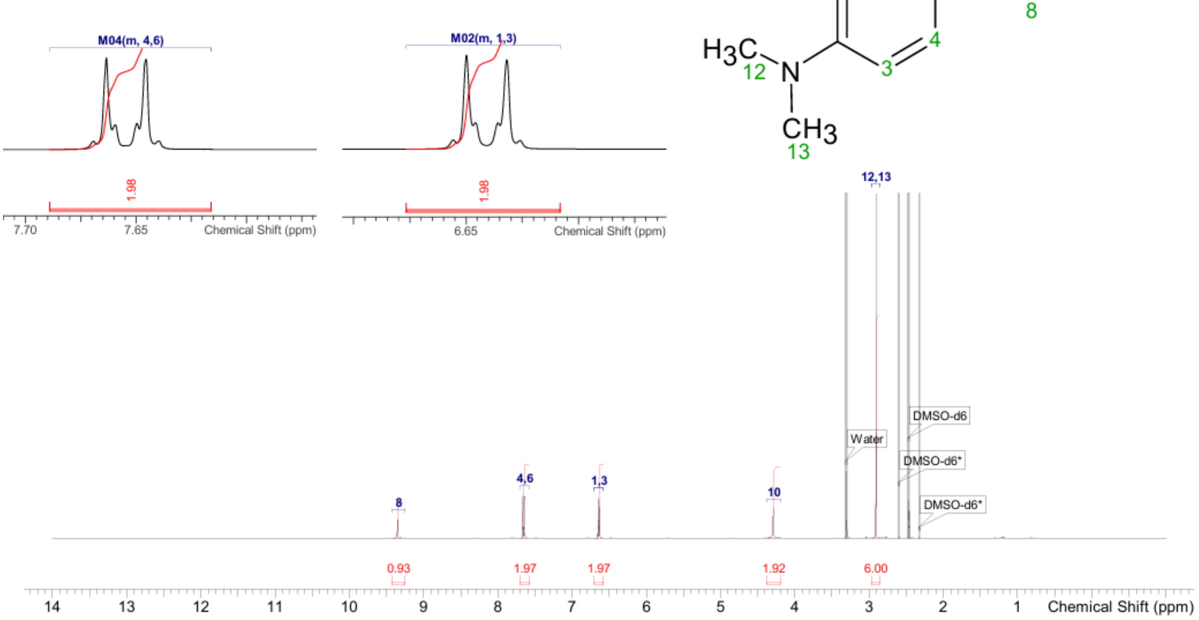

## 2.5. 4-Methoxybenzohydrazide (1e)

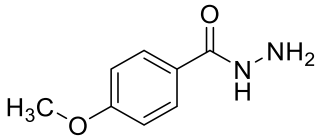


### 2.5.1. $^{1}$H NMR

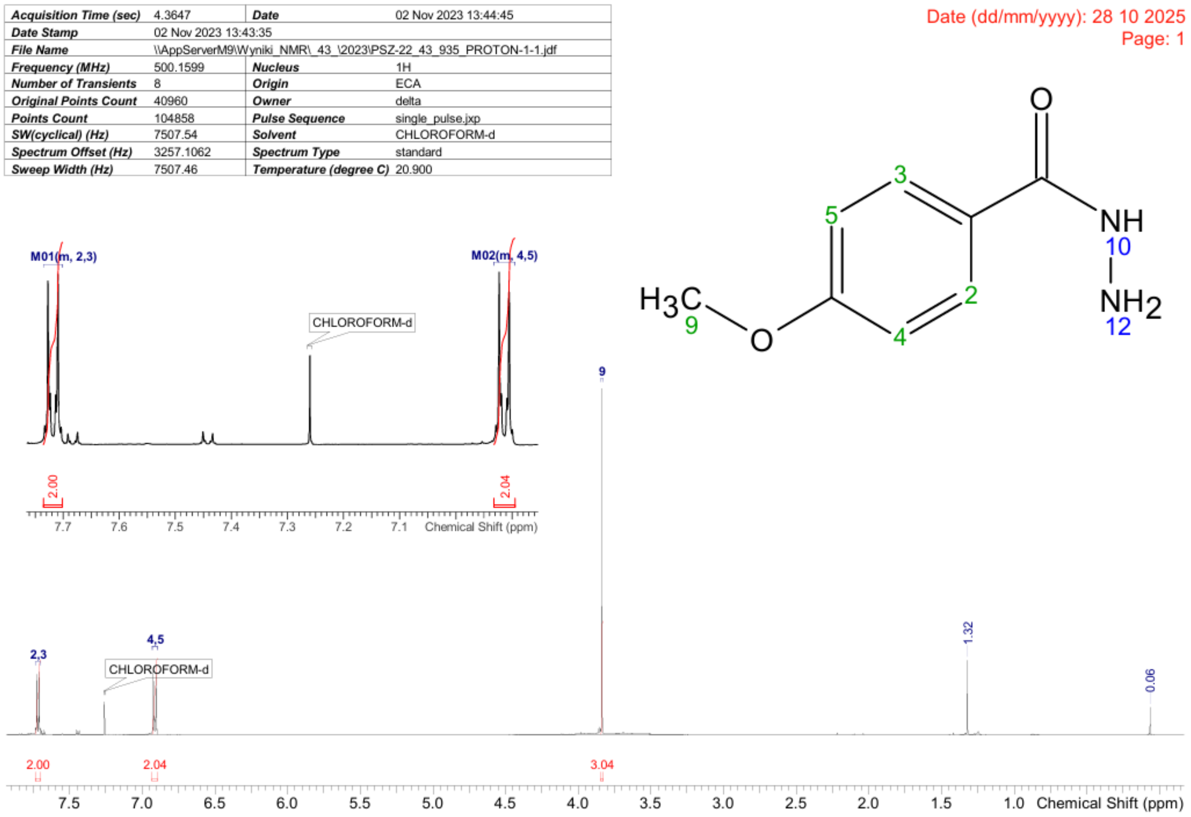

## 2.6. Phenylhydrazine (1f)

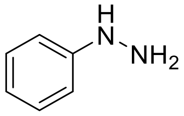


### 2.6.1. $^{1}$H NMR

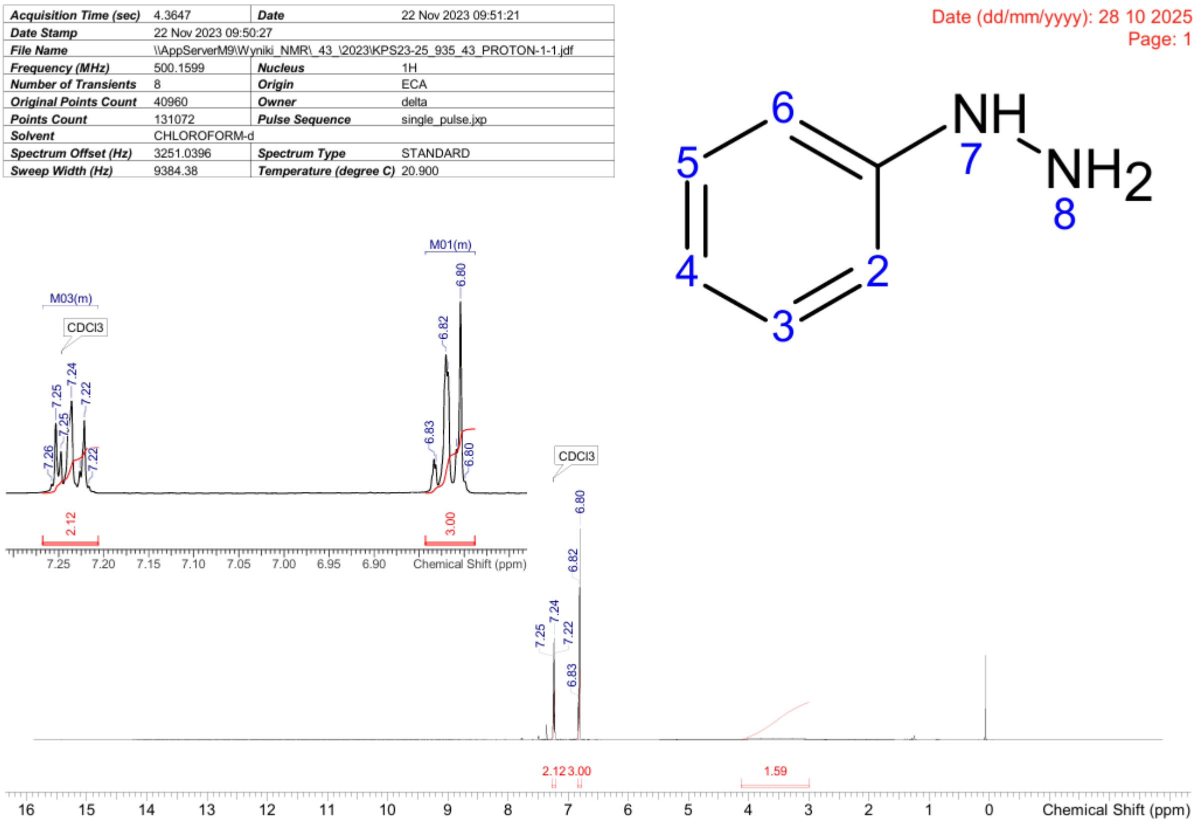

## 2.7. Benzenesulfonohydrazide (1g)

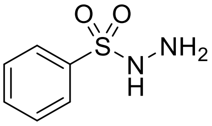

### 2.7.1. ¹H NMR

| Acquisition Time (sec) | 2.0479 | Date | 23 Sep 2025 10:27:16 | | | Date Stamp | 23 Sep 2025 10:26:37 |
|---|---|---|---|---|---|---|---|
| File Name | C:\Users\panczykk\Desktop\Widma_KPS_PZ\NMR\2023\KPS23-4_958_43_PROTON-1-1.jdf | | | | | Frequency (MHz) | 500.16 |
| Nucleus | 1H | Number of Transients | 8 | Origin | ECA | Original Points Count | 19225 |
| Owner | delta | Points Count | 131072 | Pulse Sequence | single_pulse.jxp | Solvent | DMSO-d6 |
| Spectrum Offset (Hz) | 3251.0396 | Spectrum Type | STANDARD | Sweep Width (Hz) | 9387.80 | Temperature (degree C) | 21.200 |

¹H NMR (500 MHz, DMSO-$d_6$) δ ppm 4.07 (d, $J$=2.29 Hz, 1 H), 7.53 - 7.59 (m, 2 H), 7.59 - 7.64 (m, 1 H), 7.74 - 7.79 (m, 2 H), 8.36 (br. s., 1 H)

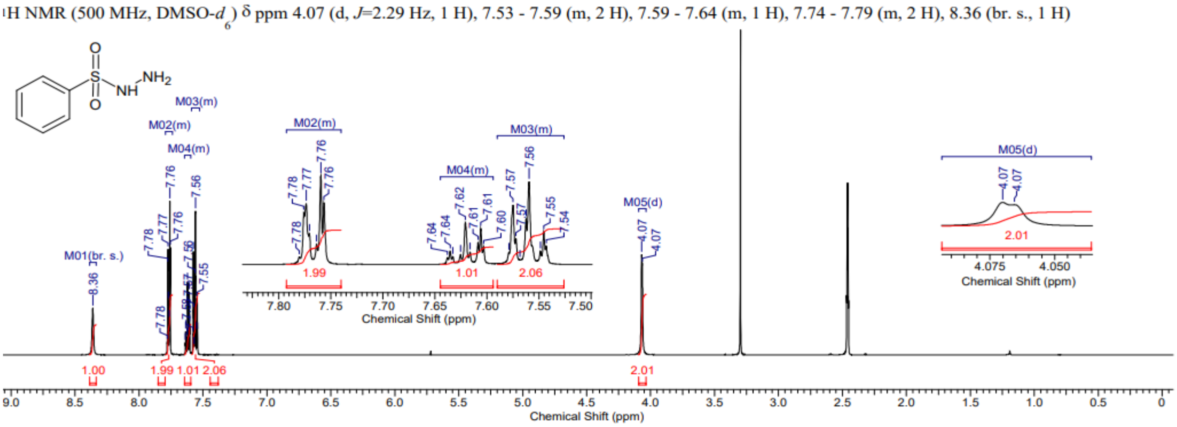


## 2.8. Thiophenecarbonylhydrazide (1h)

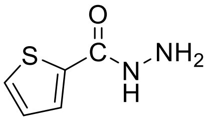

### 2.8.1. ¹H NMR

05.12.2025 01:20:53

| Acquisition Time (sec) | 2.0479 | Date | 04 Dec 2025 10:17:44 | | | Date Stamp | 04 Dec 2025 10:17:05 |
|---|---|---|---|---|---|---|---|
| File Name | C:\Users\Admin\OneDrive - Uniwersytet Jagielloński\nmr\2025\Tiof-hydrazyd_935_03_PROTON-1-1.jdf | | | | | Frequency (MHz) | 500.16 |
| Nucleus | 1H | Number of Transients | 8 | Origin | ECA | Original Points Count | 19225 |
| Owner | delta | Points Count | 131072 | Pulse Sequence | single_pulse.jxp | Solvent | DMSO-d6 |
| Spectrum Offset (Hz) | 3251.0396 | Spectrum Type | STANDARD | Sweep Width (Hz) | 9387.80 | Temperature (degree C) | 20.800 |

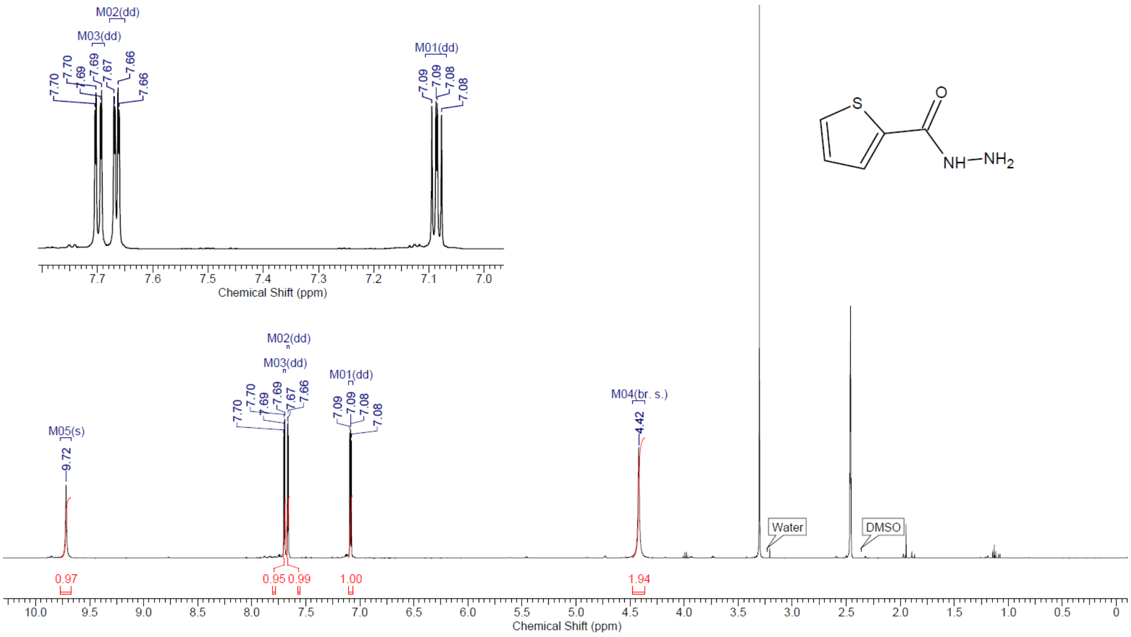

## 2.9. *N'*-(5-Oxofuran-2(5H)-ylidene)-4-(trifluoromethyl)benzohydrazide (2a)

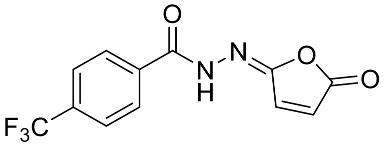

### 2.9.1. $^{1}$H NMR

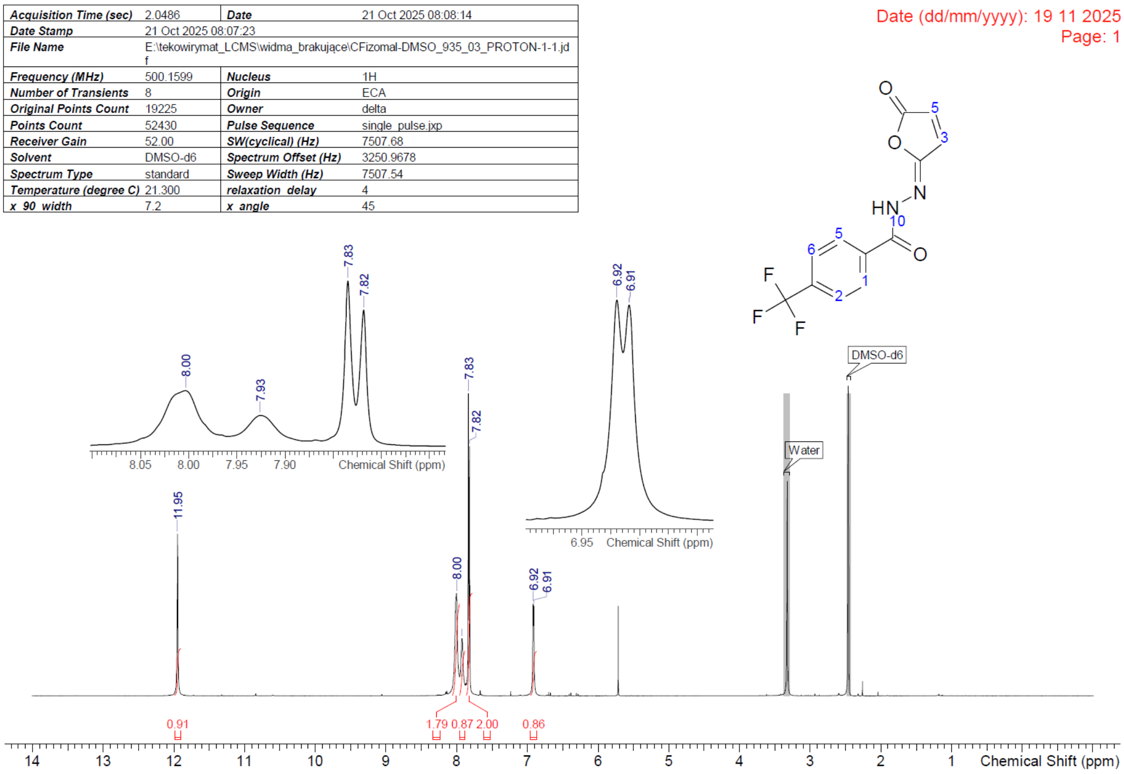

| | | | |
|---|---|---|---|
| Acquisition Time (sec) | 2.0486 | Date | 21 Oct 2025 08:08:14 |
| Date Stamp | 21 Oct 2025 08:07:23 | | |
| File Name | E:\tekowirymat_LCMS\widma_brakujące\CFizomal-DMSO_935_03_PROTON-1-1.jdf | | |
| Frequency (MHz) | 500.1599 | Nucleus | 1H |
| Number of Transients | 8 | Origin | ECA |
| Original Points Count | 19225 | Owner | delta |
| Points Count | 52430 | Pulse Sequence | single_pulse.jxp |
| Receiver Gain | 52.00 | SW(cyclical) (Hz) | 7507.68 |
| Solvent | DMSO-d6 | Spectrum Offset (Hz) | 3250.9678 |
| Spectrum Type | standard | Sweep Width (Hz) | 7507.54 |
| Temperature (degree C) | 21.300 | relaxation_delay | 4 |
| x_90_width | 7.2 | x_angle | 45 |

### 2.9.2. $^{13}$C NMR

| Acquisition Time (sec) | 1.9997 | Date | 21 Oct 2025 09:21:05 |
| --- | --- | --- | --- |
| Date Stamp | 21 Oct 2025 08:12:29 | | |
| File Name | E:\tekowirymat_LCMS\widma_brakujące\CFizomal-DMSO_935_03_CARBON-1-1.jdf | | |
| Frequency (MHz) | 125.7653 | Nucleus | 13C |
| Number of Transients | 1024 | Origin | ECA |
| Original Points Count | 78604 | Owner | delta |
| Points Count | 209716 | Pulse Sequence | carbon.jxp |
| Receiver Gain | 52.00 | SW(cyclical) (Hz) | 31446.66 |
| Solvent | DMSO-d6 | Spectrum Offset (Hz) | 12576.4551 |
| Spectrum Type | standard | Sweep Width (Hz) | 31446.51 |
| Temperature (degree C) | 21.200 | relaxation_delay | 2 |
| x_90_width | 12 | x_angle | 30 |

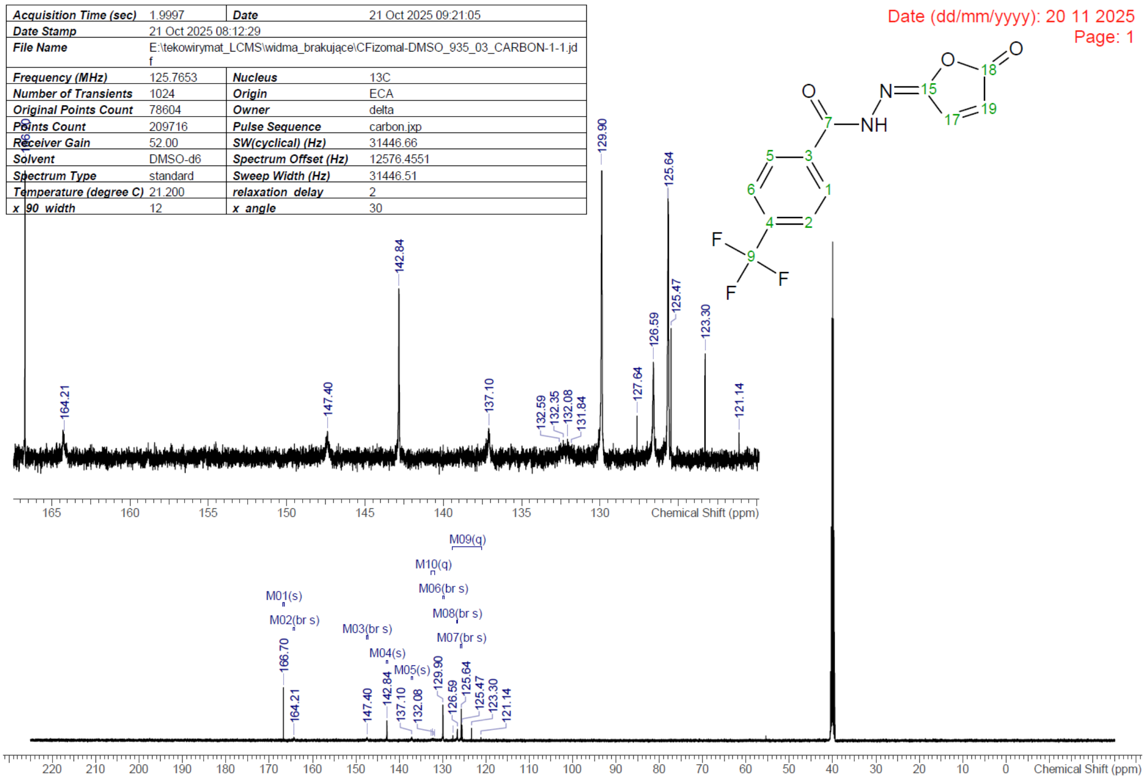


### 2.9.3. $^{19}$F NMR

| Acquisition Time (sec) | 2.9861 | Date | 21 Oct 2025 08:10:54 |
| --- | --- | --- | --- |
| Date Stamp | 21 Oct 2025 08:09:52 | | |
| File Name | E:\tekowirymat_LCMS\widma_brakujące\CFizomal-DMSO_935_03_Fluorine-1-1.jdf | | |
| Frequency (MHz) | 470.6205 | Nucleus | 19F |
| Number of Transients | 8 | Origin | ECA |
| Original Points Count | 352967 | Owner | delta |
| Points Count | 838862 | Pulse Sequence | single_pulse.jxp |
| Receiver Gain | 62.00 | SW(cyclical) (Hz) | 94562.79 |
| Solvent | DMSO-d6 | Spectrum Offset (Hz) | -47062.1016 |
| Spectrum Type | standard | Sweep Width (Hz) | 94562.68 |
| Temperature (degree C) | 21.200 | relaxation_delay | 4 |
| x_90_width | 7.79 | x_angle | 45 |

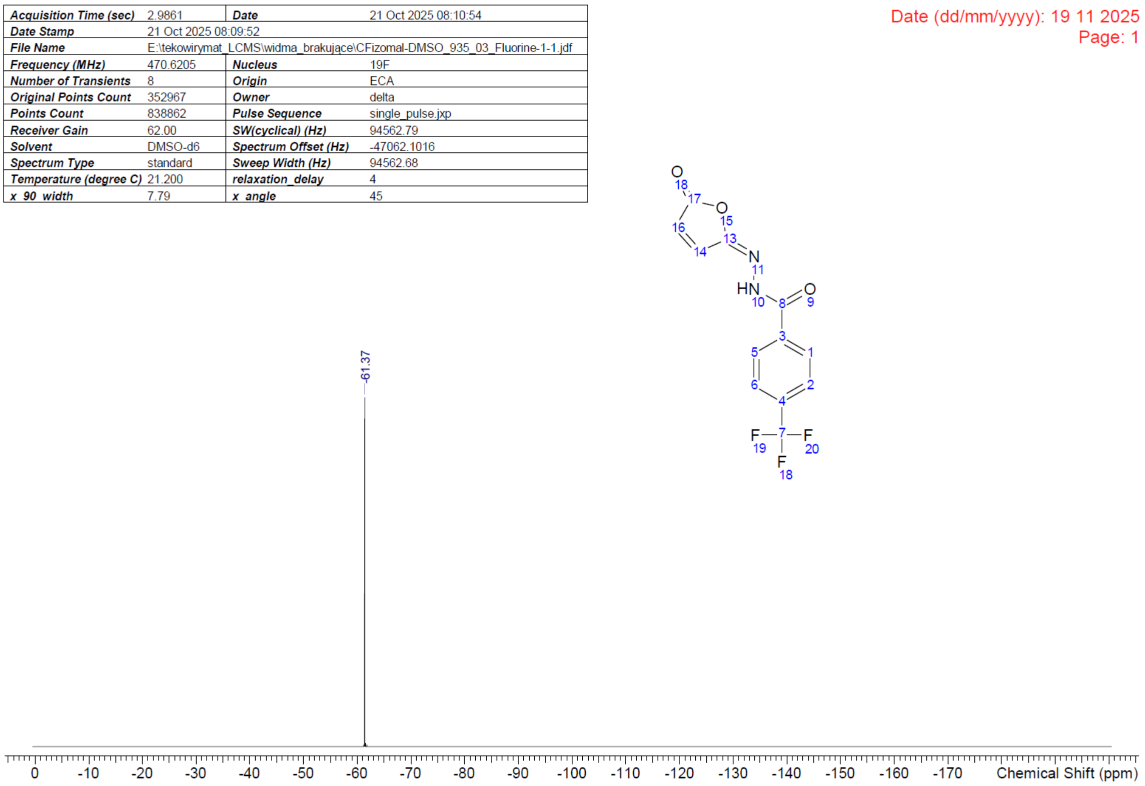

### 2.9.4. LCMS

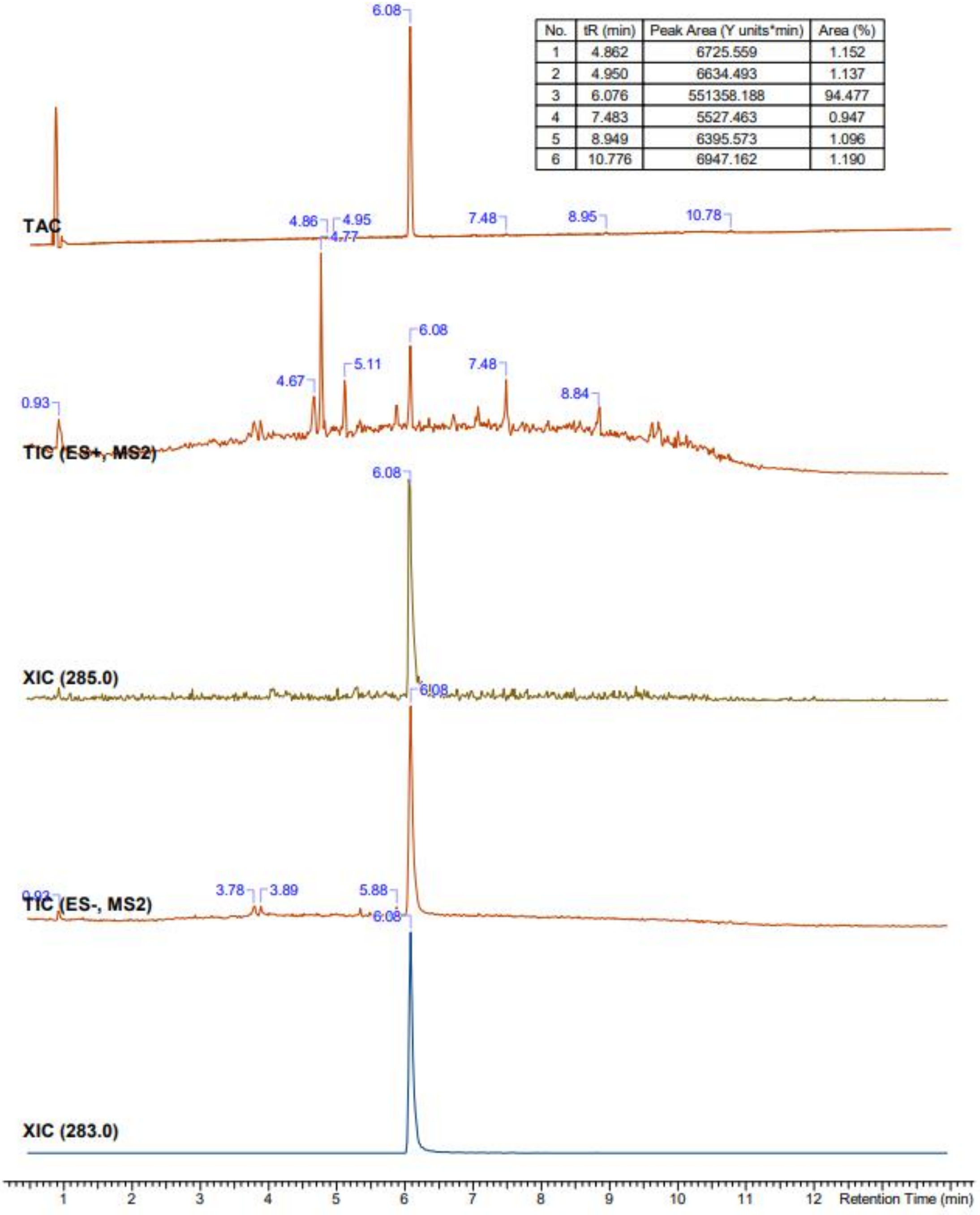


| No. | tR (min) | Peak Area (Y units*min) | Area (%) |
|---|---|---|---|
| 1 | 4.862 | 6725.559 | 1.152 |
| 2 | 4.950 | 6634.493 | 1.137 |
| 3 | 6.076 | 551358.188 | 94.477 |
| 4 | 7.483 | 5527.463 | 0.947 |
| 5 | 8.949 | 6395.573 | 1.096 |
| 6 | 10.776 | 6947.162 | 1.190 |

| Compo | tR(min) | Mass(Ao) | [M+H]+ | [M-H]- | M+· | M-· | MF | Structure | Area (%) |
|---|---|---|---|---|---|---|---|---|---|
| 1 | 6.076 | 284.041 | 285.048 | 283.034 | 284.040 | 284.041 | $C_{12}H_7F_3N_2O_3$ | 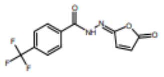 | TIC (ES-, MS2) = 89.94<br>Single XIC = 100.00<br>TIC (ES+, MS2) = 15.54<br>Single XIC = 100.00<br>TAC = 94.48 |

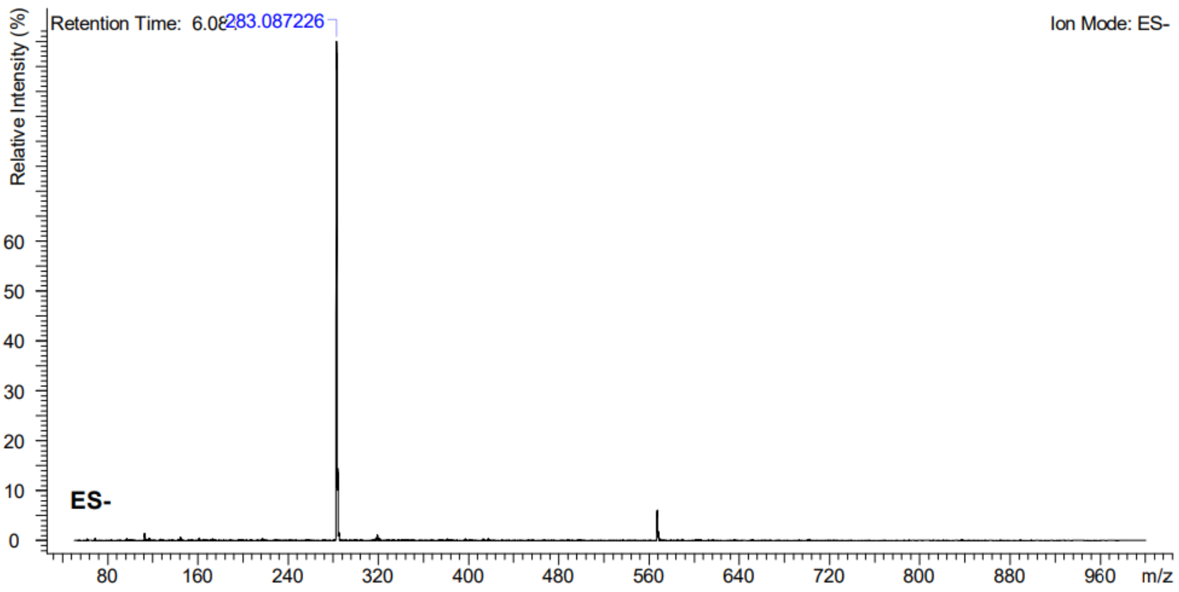
Retention Time: 6.08
283.087226
Ion Mode: ES-
Relative Intensity (%)
ES-
0
10
20
30
40
50
60
80
160
240
320
400
480
560
640
720
800
880
960
m/z

## 2.10. *Tert*-butyl 2-(5-oxofuran-2(5*H*)-ylidene)hydrazine-1-carboxylate (2b)

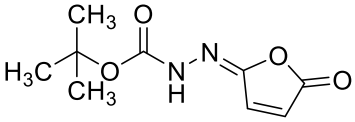

-

### 2.10.1. $^{1}$H NMR

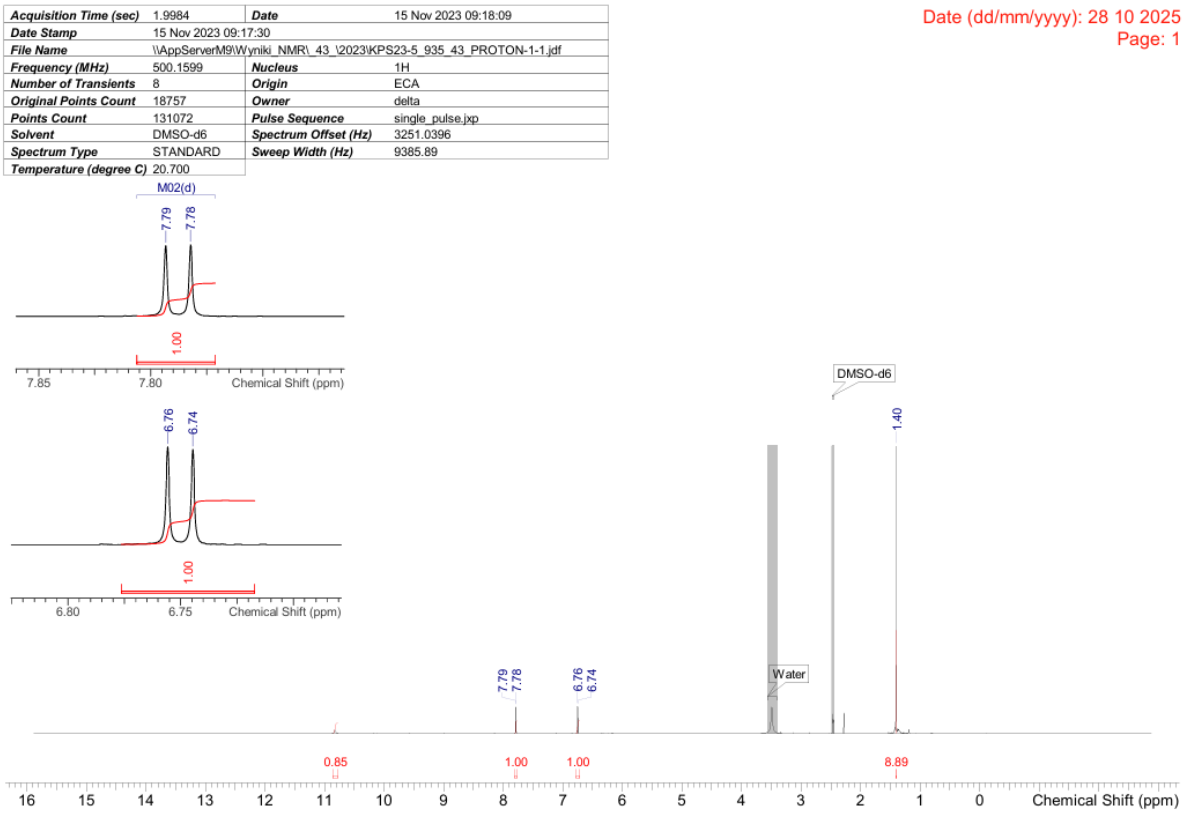

## 2.10.2. $^{13}$C NMR

| Acquisition Time (sec) | 1.9996 | Date | 15 Nov 2023 10:10:44 |
|---|---|---|---|
| Date Stamp | 15 Nov 2023 09:18:41 | | |
| File Name | \\AppServerM9\Wyniki_NMR_43_\2023\KPS23-5_935_43_CARBON-1-1.jdf | | |
| Frequency (MHz) | 125.7653 | Nucleus | 13C |
| Number of Transients | 1024 | Origin | ECA |
| Original Points Count | 78604 | Owner | delta |
| Points Count | 131072 | Pulse Sequence | carbon.jxp |
| Solvent | DMSO-d6 | Spectrum Offset (Hz) | 12576.5293 |
| Sweep Width (Hz) | 39310.18 | Temperature (degree C) | 20.800 |

Date (dd/mm/yyyy): 28 10 2025
Page: 1

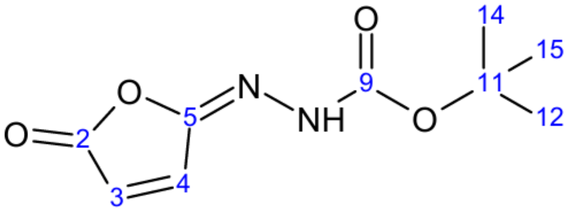


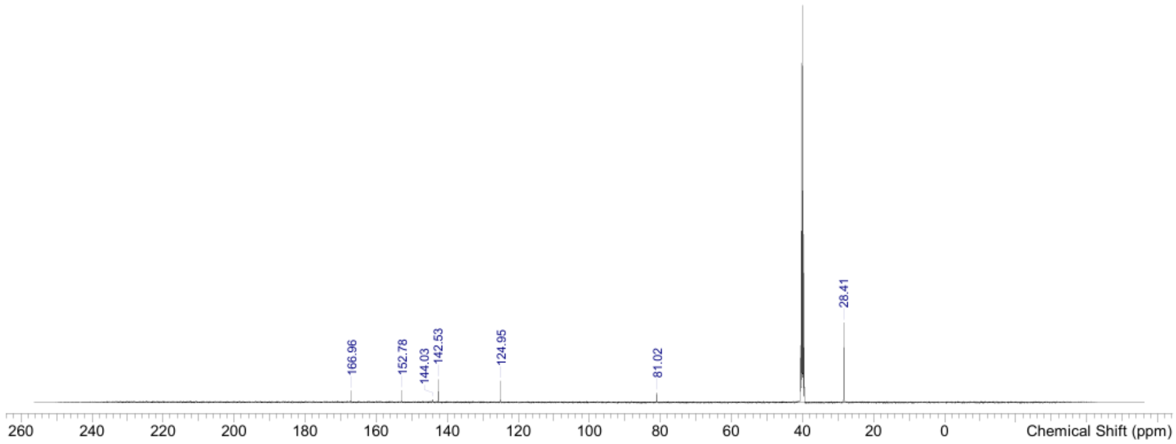

## **2.10.3.** LCMS

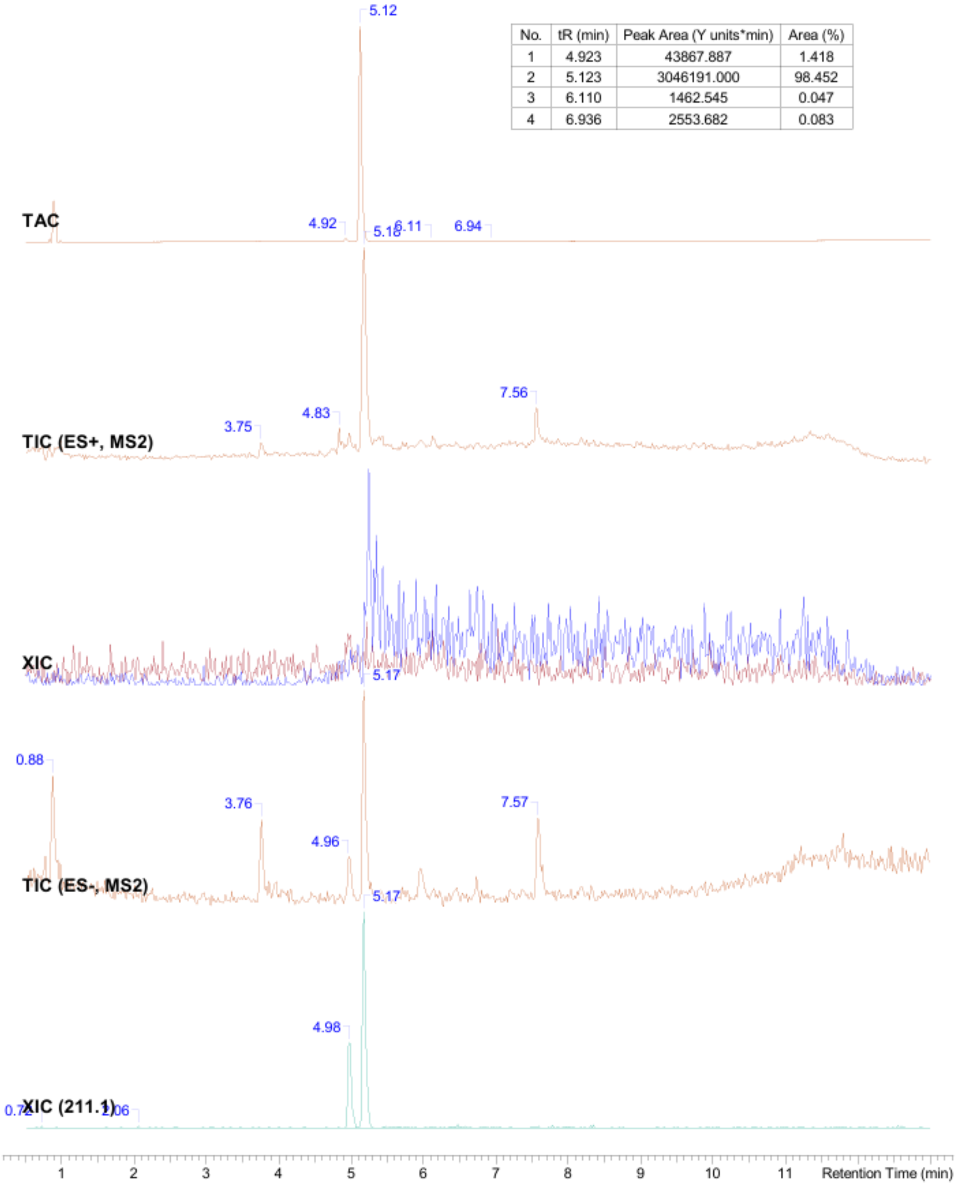


| No. | tR (min) | Peak Area (Y units*min) | Area (%) |
|---|---|---|---|
| 1 | 4.923 | 43867.887 | 1.418 |
| 2 | 5.123 | 3046191.000 | 98.452 |
| 3 | 6.110 | 1462.545 | 0.047 |
| 4 | 6.936 | 2553.682 | 0.083 |

| Compo | tR(min) | Mass(Ao) | [M+H]+ | [M-H]- | M+· | M-· | MF | Structure | Area (%) |
|---|---|---|---|---|---|---|---|---|---|
| 1 | 5.123 | 212.080 | 213.087 | 211.072 | 212.079 | 212.080 | $C_9H_{12}N_2O_4$ | 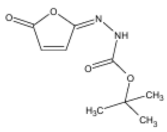 | TIC (ES-, MS2) = 37.19<br>Single XIC = 70.05<br>TIC (ES+, MS2) = 72.48<br>TAC = 98.45 |

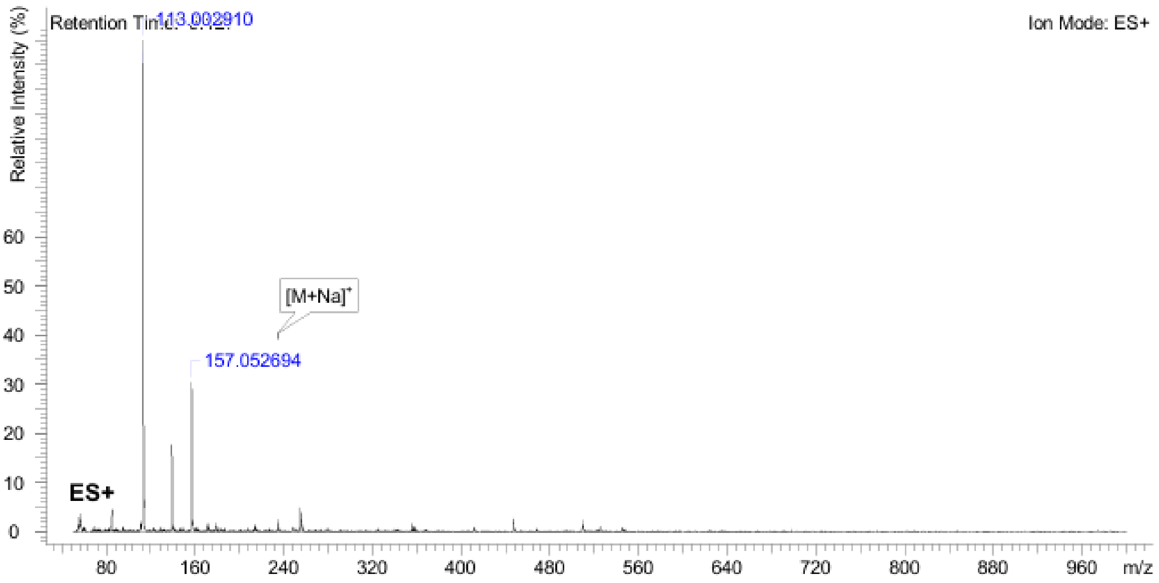
Retention Time: 5.12
113.002910
Ion Mode: ES+
Relative Intensity (%)
[M+Na]+
157.052694
ES+
m/z

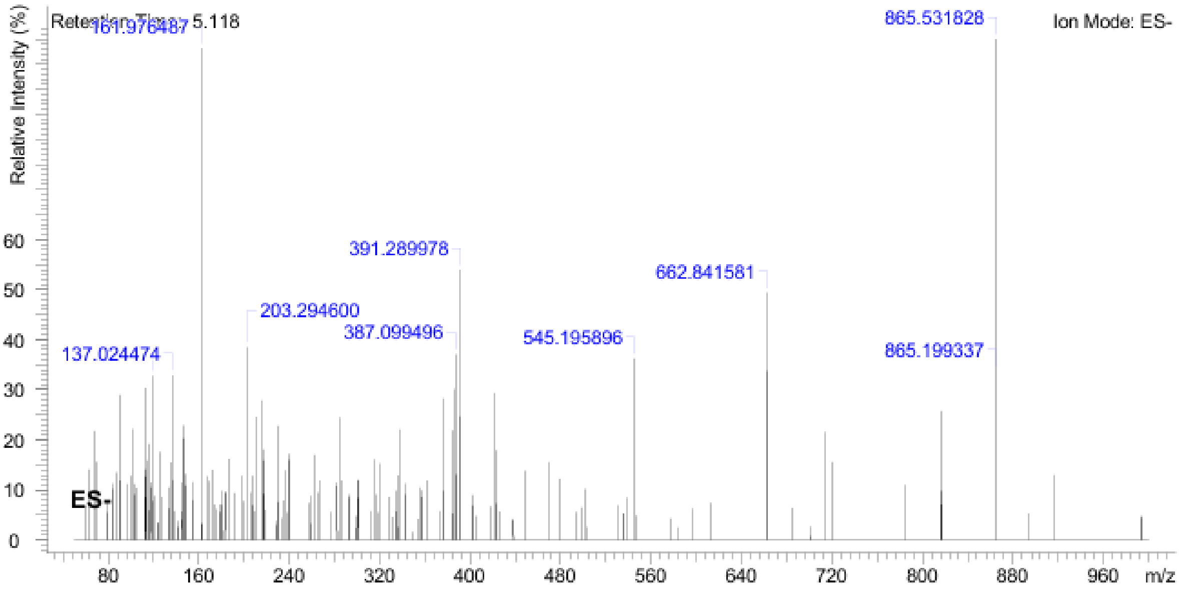
Retention Time: 5.118
161.976487
865.531828
Ion Mode: ES-
Relative Intensity (%)
391.289978
662.841581
203.294600
387.099496
545.195896
137.024474
865.199337
ES-
m/z

## 2.11. *N'*-(5-Oxofuran-2(*5H*)-ylidene)benzohydrazide (2c)

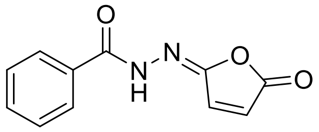

### 2.11.1. $^{1}$H NMR

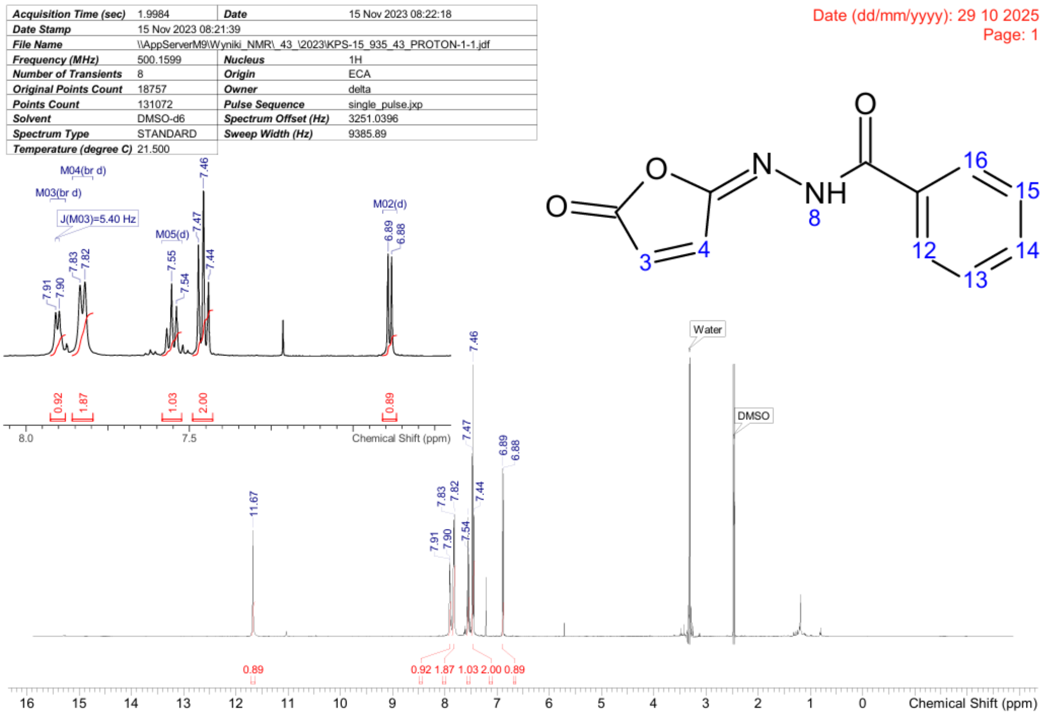


### 2.11.2. $^{13}$C NMR

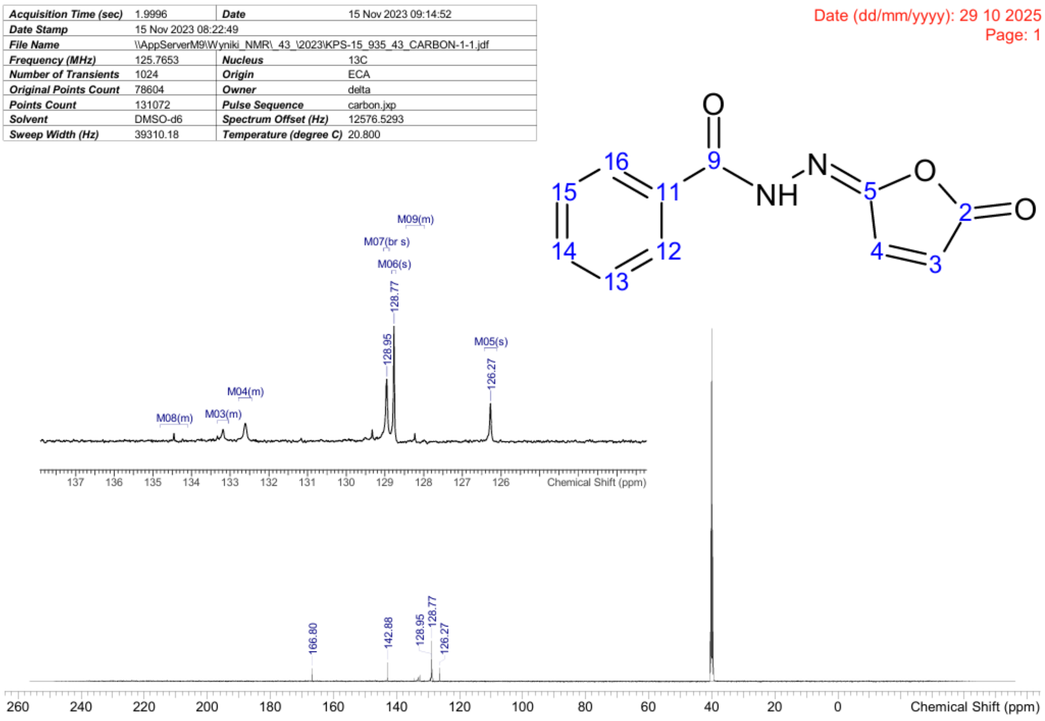

## 2.11.3. LCMS

Advanced Chemistry Development

| No. | tR (min) | Peak Area (Y units*min) | Area (%) |
|---|---|---|---|
| 1 | 0.835 | 13915.439 | 0.146 |
| 2 | 0.887 | 58882.070 | 0.618 |
| 3 | 0.983 | 12784.523 | 0.134 |
| 4 | 3.023 | 30060.084 | 0.315 |
| 5 | 4.088 | 30974.262 | 0.325 |
| 6 | 4.218 | 47547.813 | 0.499 |
| 7 | 4.477 | 9256329.000 | 97.075 |
| 8 | 5.353 | 18494.859 | 0.194 |
| 9 | 5.609 | 29752.229 | 0.312 |
| 10 | 6.882 | 12299.273 | 0.129 |
| 11 | 7.668 | 24147.623 | 0.253 |

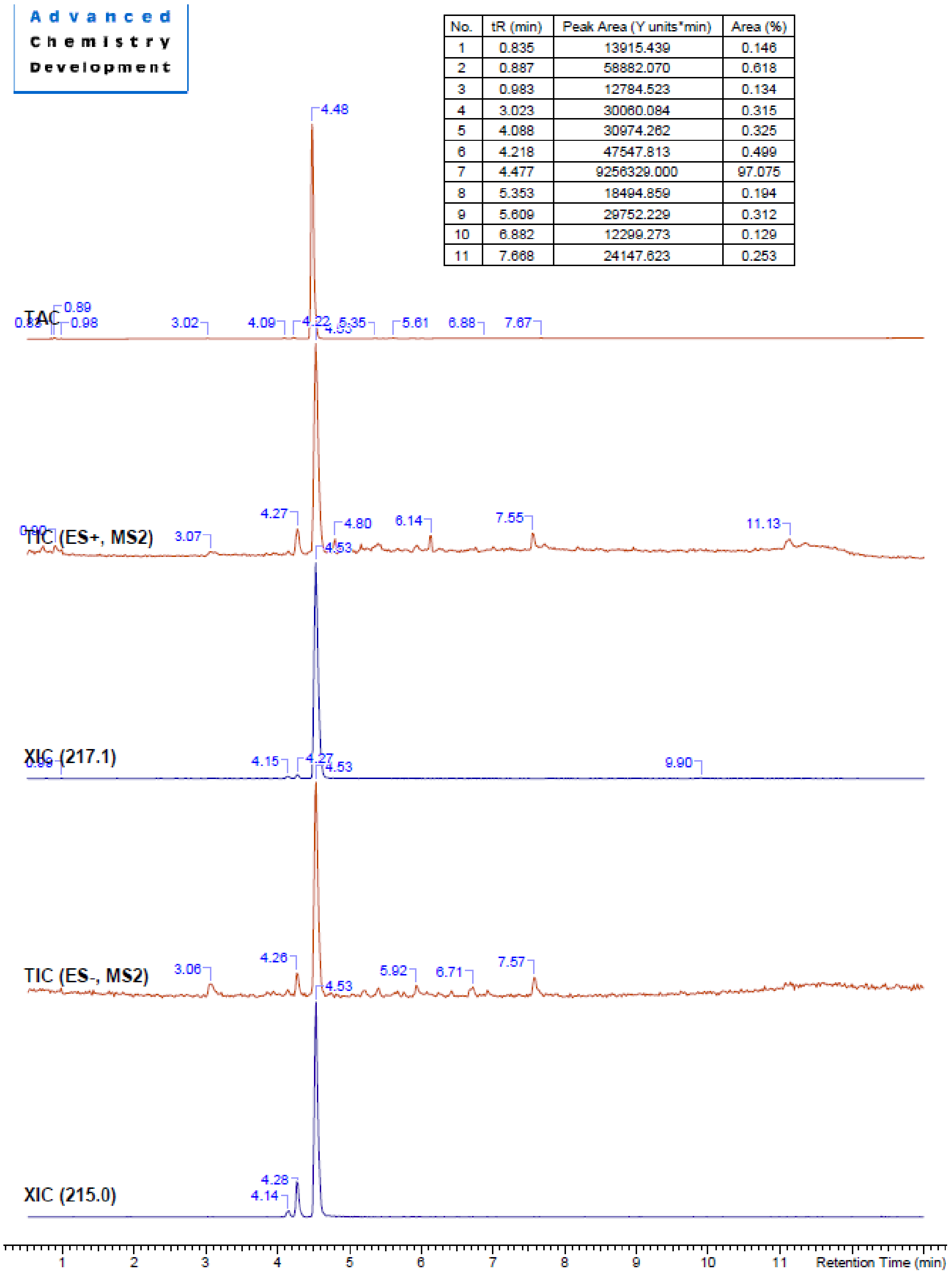


| tR (min) | Mass (Ao) | [M+H]+ | [M-H]- | Area (%) |
|---|---|---|---|---|
| 4.477 | 216.053 | 217.061 | 215.046 | TIC (ES-, MS2) = 69.94<br>XIC = 76.88<br>TIC (ES+, MS2) = 70.06<br>XIC = 89.10<br>TAC = 97.08 |

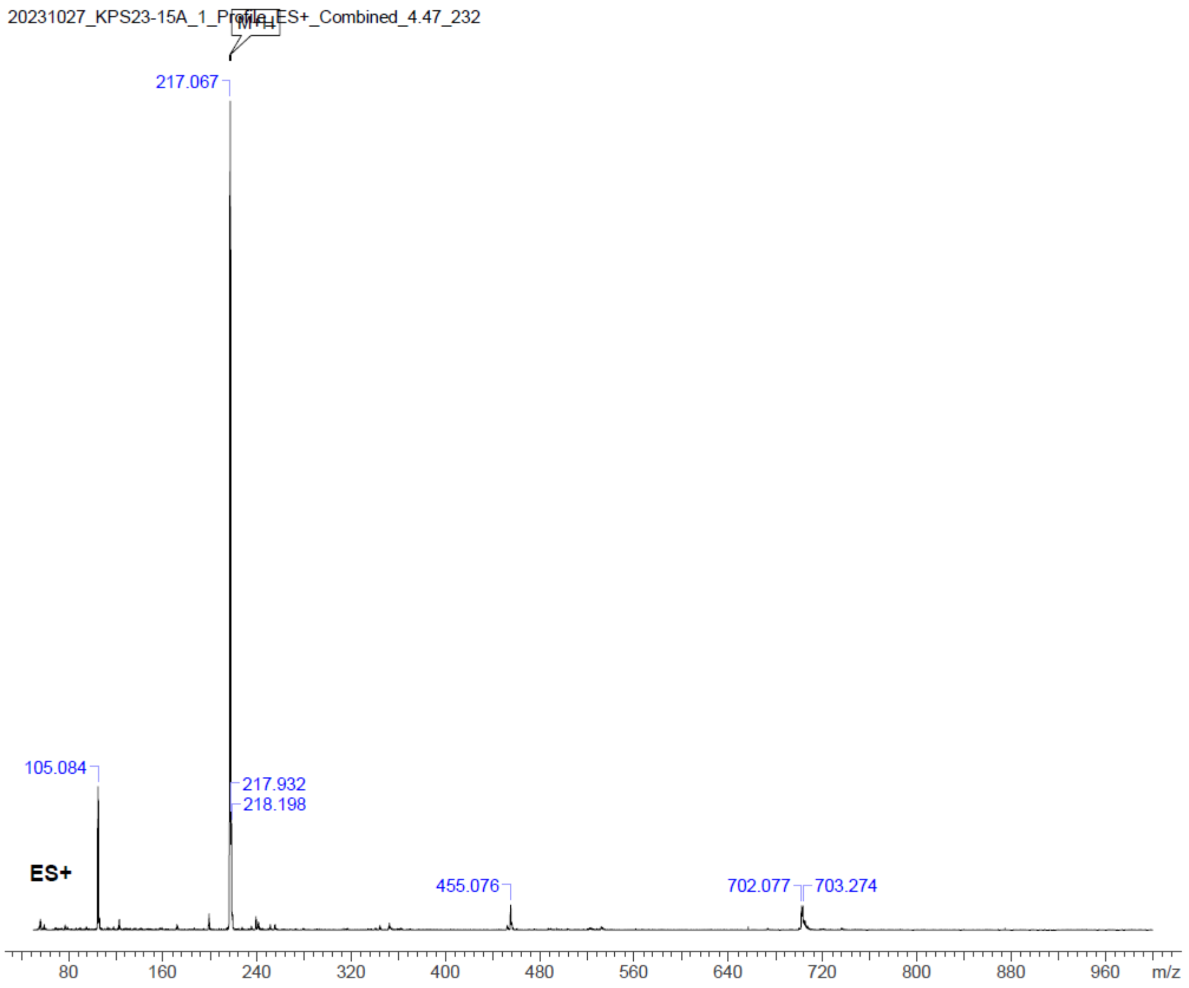

20231027_KPS23-15A_1_Profile_ES+_Combined_4.47_232
M+H
217.067
105.084
217.932
218.198
ES+
455.076
702.077
703.274
80
160
240
320
400
480
560
640
720
800
880
960
m/z

## 2.12.4-(Dimethylamino)-N'-(5-oxofuran-2(*5H*)-ylidene)benzohydrazide (2d)

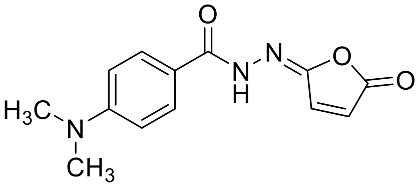

### 2.12.1. $^{1}$H NMR

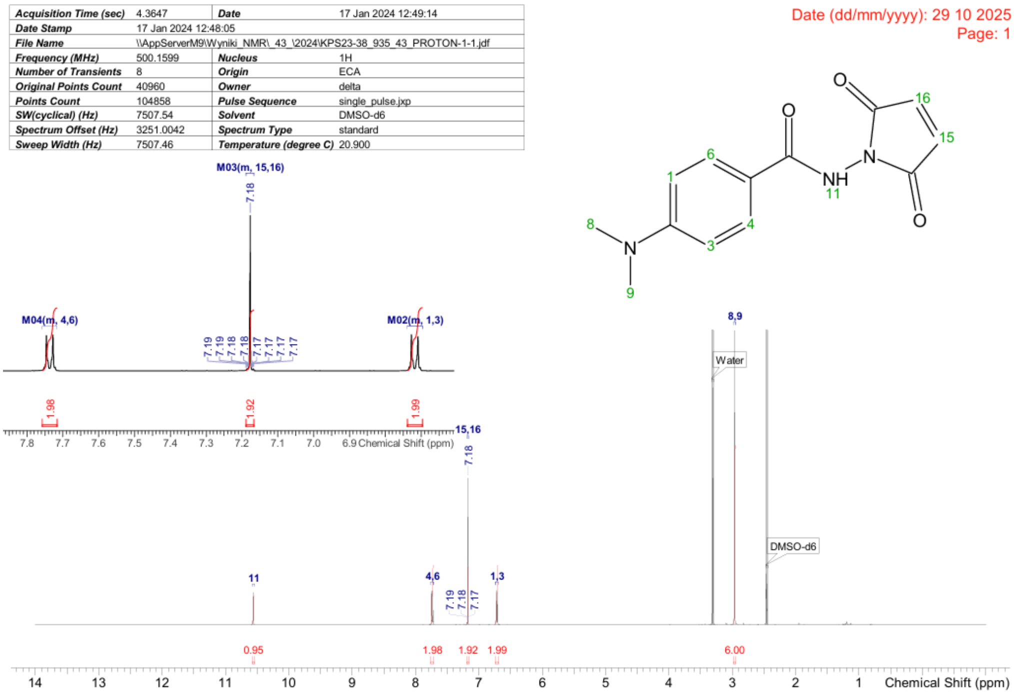

### 2.12.2. $^{13}$C NMR

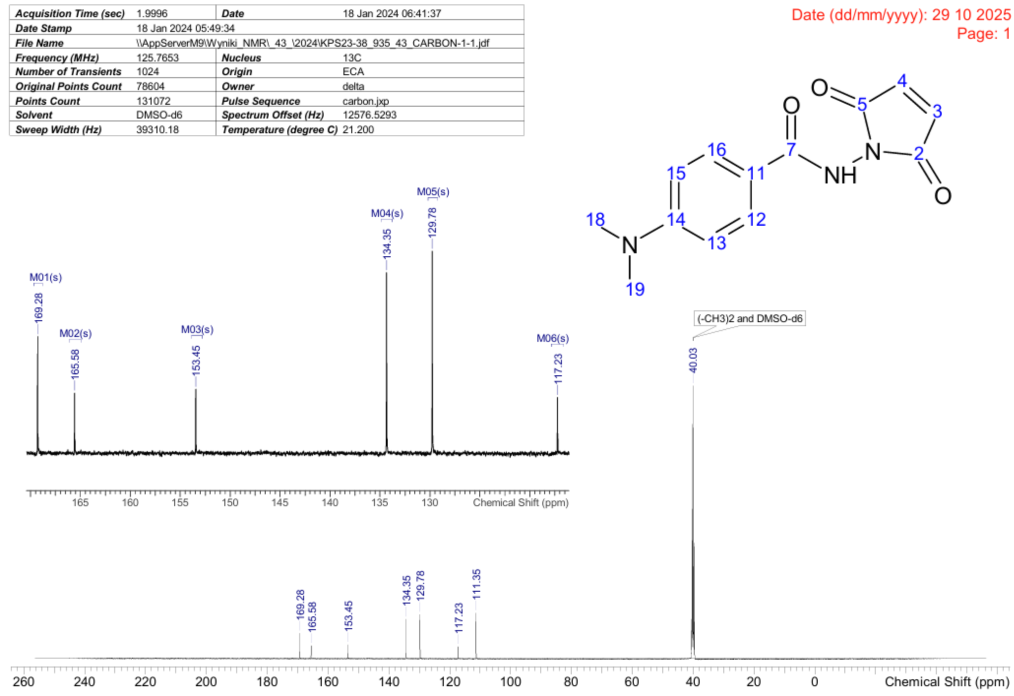

**2.12.3.** LCMS

Development

| No. | tR (min) | Peak Area (Y units*min) | Area (%) |
|---|---|---|---|
| 1 | 3.874 | 146960.000 | 1.991 |
| 2 | 4.439 | 21416.916 | 0.290 |
| 3 | 4.828 | 44516.508 | 0.603 |
| 4 | 4.908 | 19354.879 | 0.262 |
| 5 | 5.251 | 7132842.500 | 96.652 |
| 6 | 6.130 | 14795.882 | 0.200 |

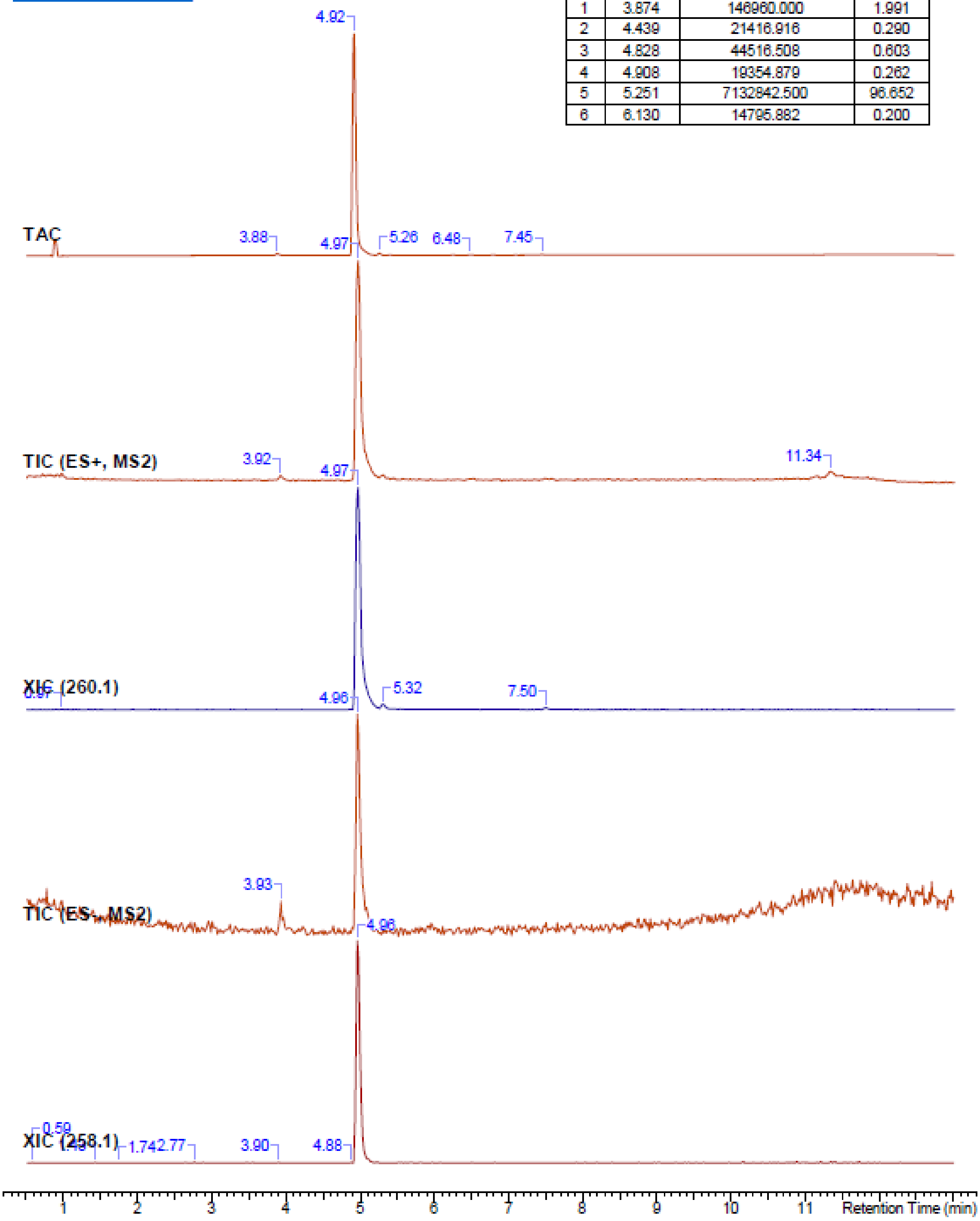


| tR (min) | Mass (Ao) | [M+H]+ | [M-H]- | Area (%) |
|---|---|---|---|---|
| 5.251 | 259.096 | 260.103 | 258.088 | TIC (ES-, MS2) = 82.72<br>XIC = 55.17<br>TIC (ES+, MS2) = 86.09<br>XIC = 75.98<br>TAC = 96.65 |

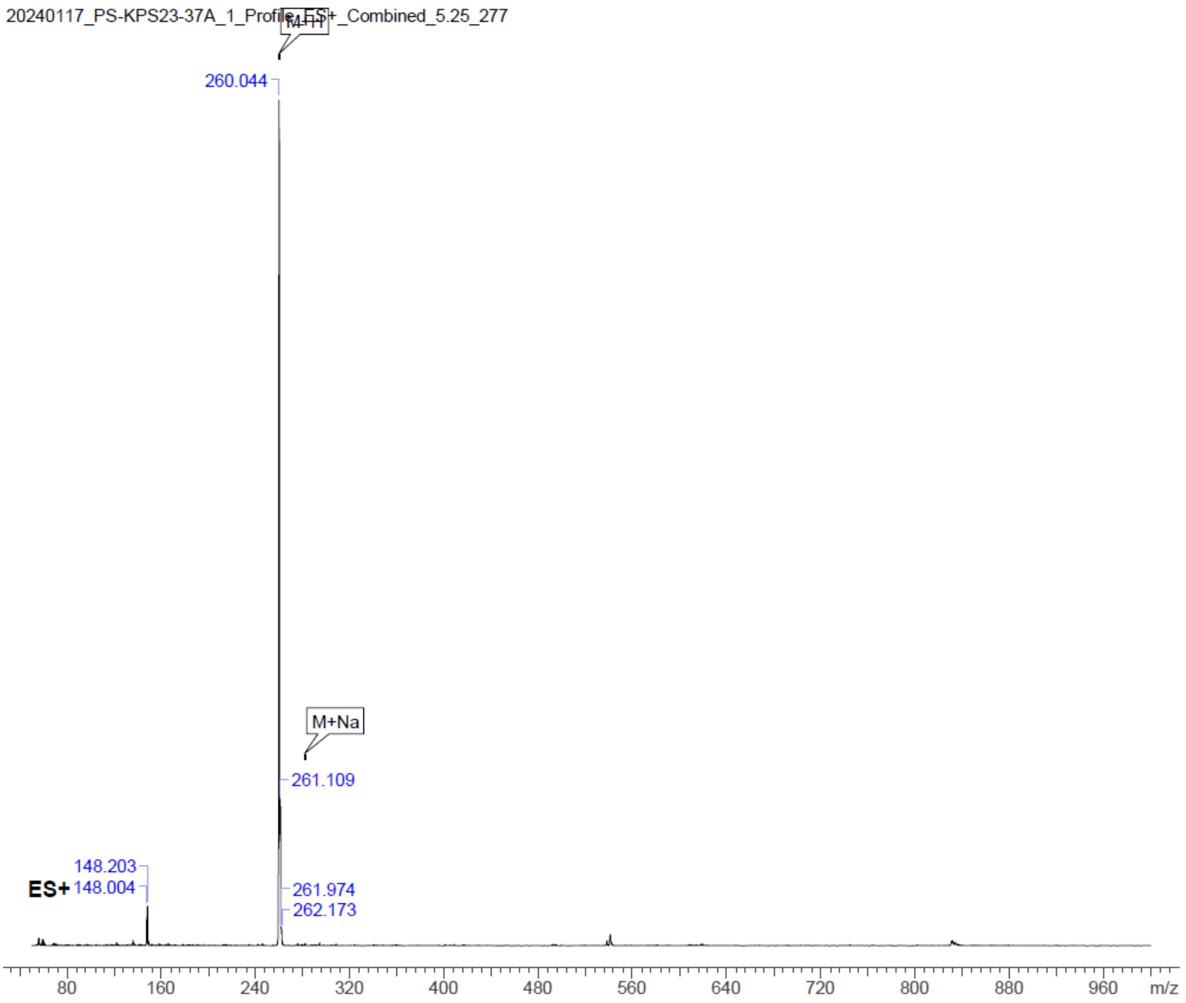
20240117_PS-KPS23-37A_1_Profile_ES+_Combined_5.25_277
M+H
260.044
M+Na
261.109
148.203
ES+
148.004
261.974
262.173
80
160
240
320
400
480
560
640
720
800
880
960
m/z

## 2.13.4-Methoxy-*N'*-(5-oxofuran-2(*5H*)-ylidene)benzohydrazide (2e)

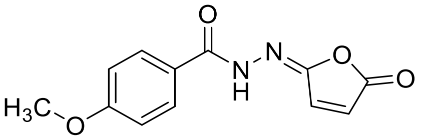


### 2.13.1. $^{1}$H NMR

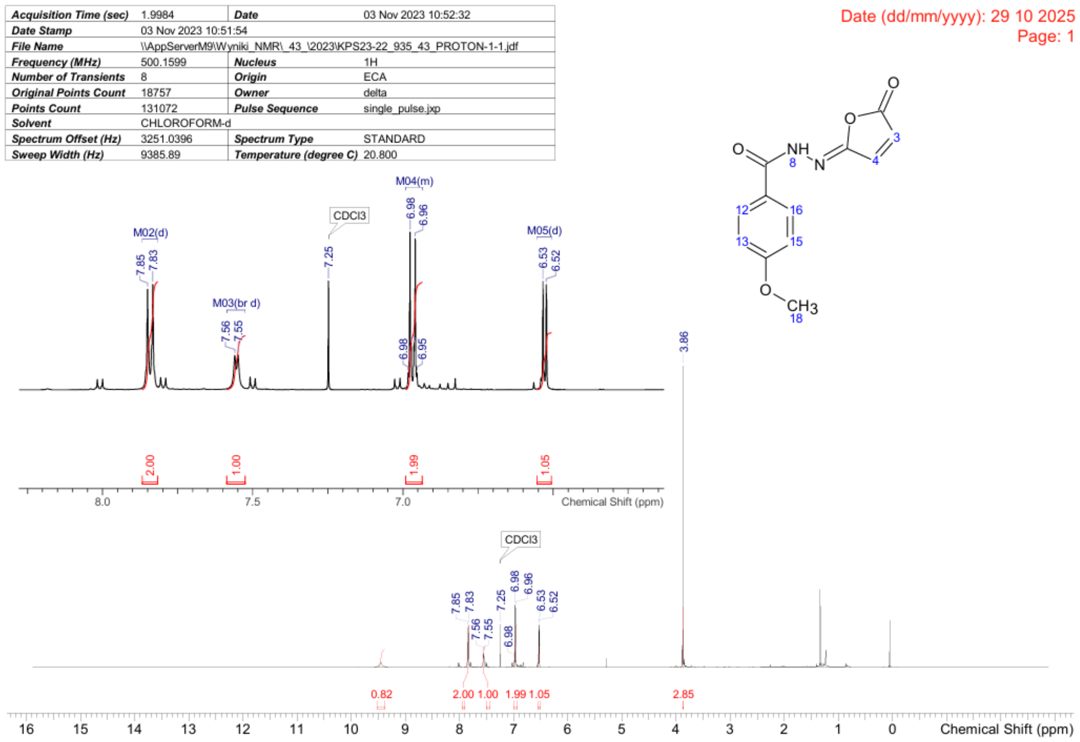


### 2.13.2. $^{13}$C NMR

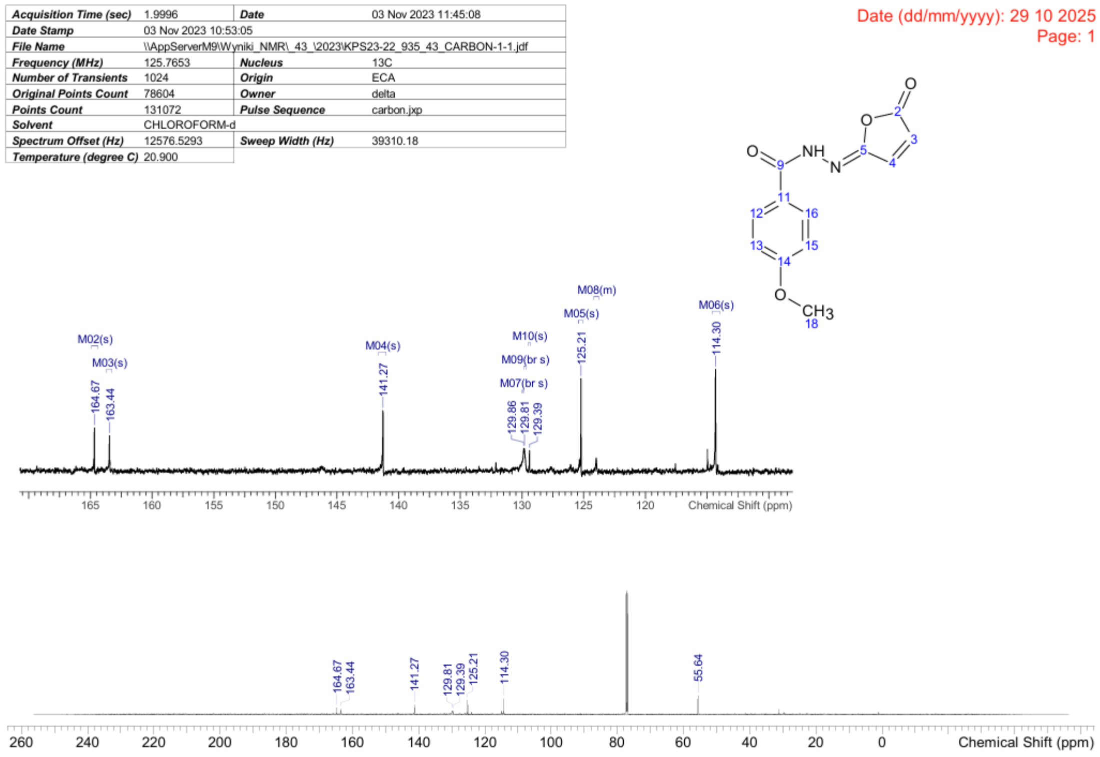

**2.13.3.** LCMS

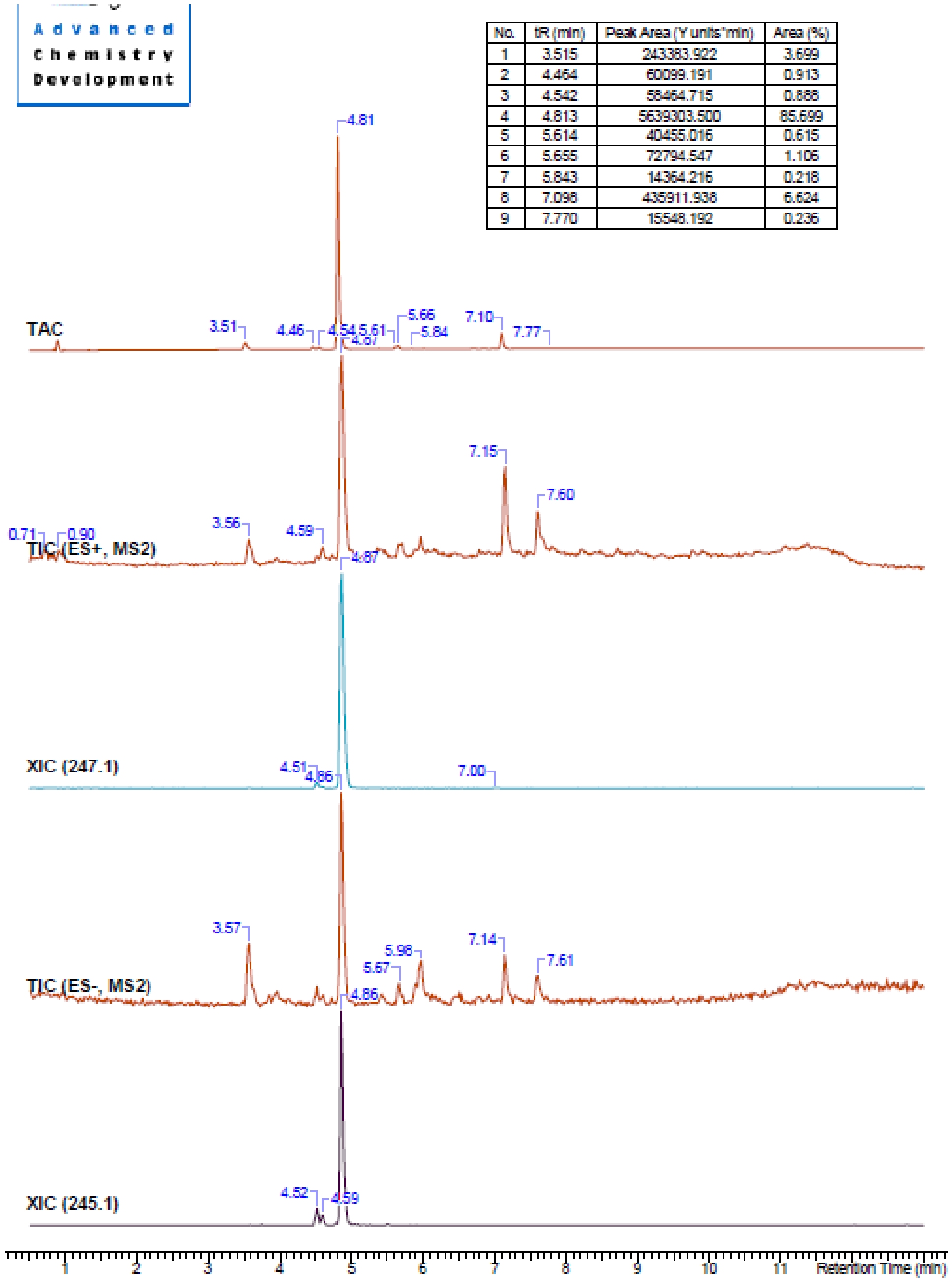


| No. | tR (min) | Peak Area (Y units*min) | Area (%) |
|---|---|---|---|
| 1 | 3.515 | 243383.922 | 3.699 |
| 2 | 4.464 | 60099.191 | 0.913 |
| 3 | 4.542 | 58464.715 | 0.888 |
| 4 | 4.813 | 5639303.500 | 85.699 |
| 5 | 5.614 | 40455.016 | 0.615 |
| 6 | 5.655 | 72794.547 | 1.106 |
| 7 | 5.843 | 14364.216 | 0.218 |
| 8 | 7.098 | 435911.938 | 6.624 |
| 9 | 7.770 | 15548.192 | 0.236 |

| tR (min) | Mass (Ao) | [M+H]+ | [M-H]- | Area (%) |
|---|---|---|---|---|
| 4.813 | 246.064 | 247.071 | 245.057 | TIC (ES-, MS2) = 44.57<br>XIC = 79.58<br>TIC (ES+, MS2) = 49.85<br>XIC = 90.98<br>TAC = 85.70 |

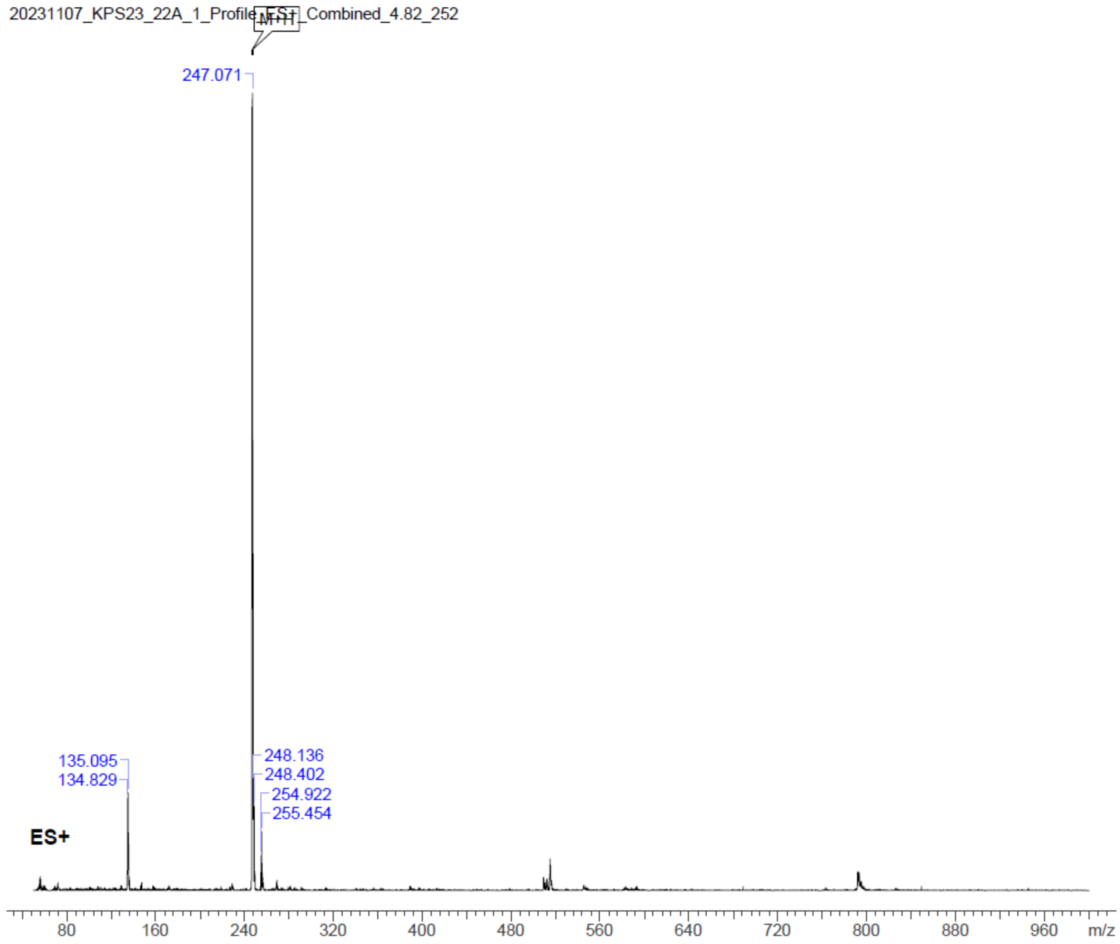

20231107_KPS23_22A_1_Profile_ES+_Combined_4.82_252
M+H
247.071
135.095
134.829
248.136
248.402
254.922
255.454
ES+
80
160
240
320
400
480
560
640
720
800
880
960
m/z

## 2.14.5-(2-Phenylhydrazineylidene)furan-2(*5H*)-one (2f)

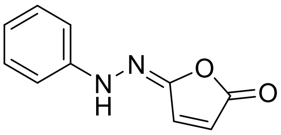

### 2.14.1. $^{1}$H NMR

| Acquisition Time (sec) | 4.3647 | Date | 09 Feb 2024 13:32:57 |
|---|---|---|---|
| Date Stamp | 09 Feb 2024 13:31:47 | | |
| File Name | \\AppServerM9\Wyniki_NMR_43_\2024\KPS24-7-fr14-25_935_43_PROTON-1-1.jdf | | |
| Frequency (MHz) | 500.1599 | Nucleus | 1H |
| Number of Transients | 8 | Origin | ECA |
| Original Points Count | 40960 | Owner | delta |
| Points Count | 104858 | Pulse Sequence | single_pulse.jxp |
| SW(cyclical) (Hz) | 7507.54 | Solvent | CHLOROFORM-d |
| Spectrum Offset (Hz) | 3251.0042 | Spectrum Type | standard |
| Sweep Width (Hz) | 7507.46 | Temperature (degree C) | 20.200 |

Date (dd/mm/yyyy): 29 10 2025
Page: 2

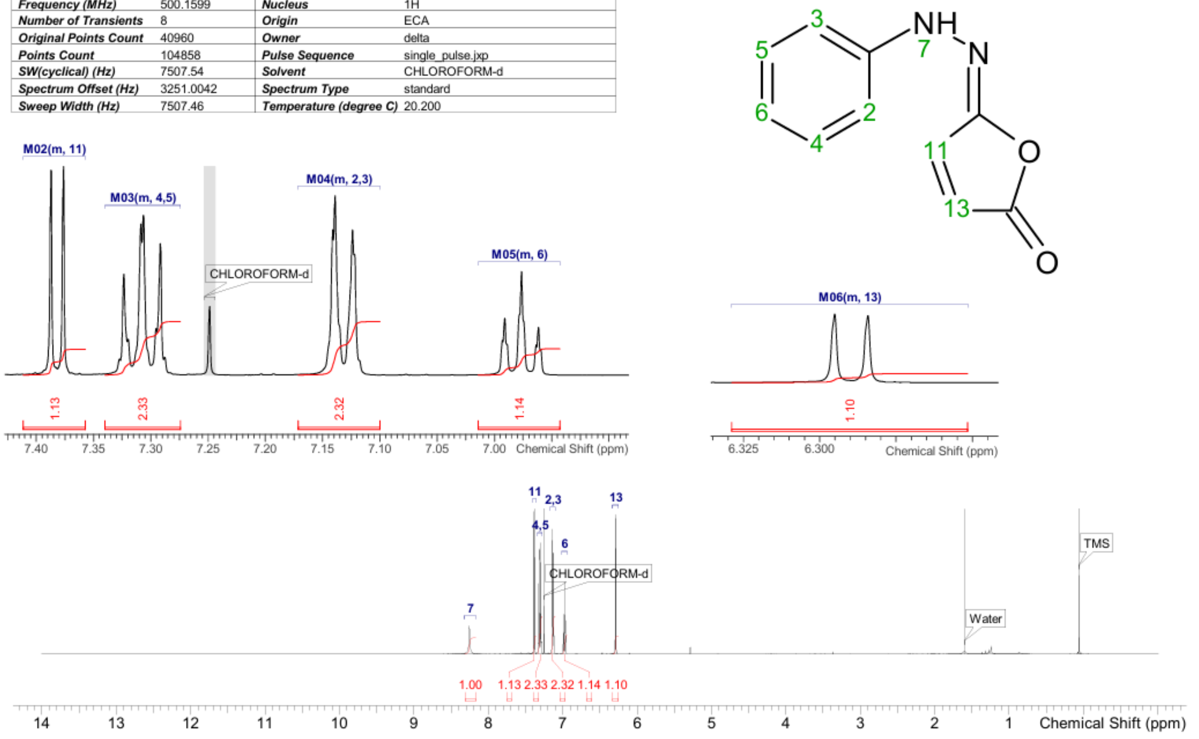


### 2.14.2. $^{13}$C NMR

| Acquisition Time (sec) | 1.9996 | Date | 09 Feb 2024 12:20:12 | | | Date Stamp | 09 Feb 2024 11:28:09 |
|---|---|---|---|---|---|---|---|
| File Name | C:\Users\panczykk\Desktop\Widma_KPS_PZ\NMR\2024\KPS24-7-fr14-25_935_43_CARBON-1-1.jdf | | | | | Frequency (MHz) | 125.77 |
| Nucleus | 13C | Number of Transients | 1024 | Origin | ECA | Original Points Count | 78604 |
| Owner | delta | Points Count | 131072 | Pulse Sequence | carbon.jxp | Solvent | CHLOROFORM-d |
| Spectrum Offset (Hz) | 12576.5293 | Sweep Width (Hz) | 39310.18 | Temperature (degree C) | 20.100 | | |

$^{13}$C NMR (126 MHz, CHLOROFORM-*d*) δ ppm 113.41 (s, 1 C), 120.11 (s, 1 C), 122.29 (s, 1 C), 129.57 (s, 1 C), 139.77 (s, 1 C), 141.41 (s, 1 C), 142.54 (s, 1 C), 166.42 (s, 1 C)

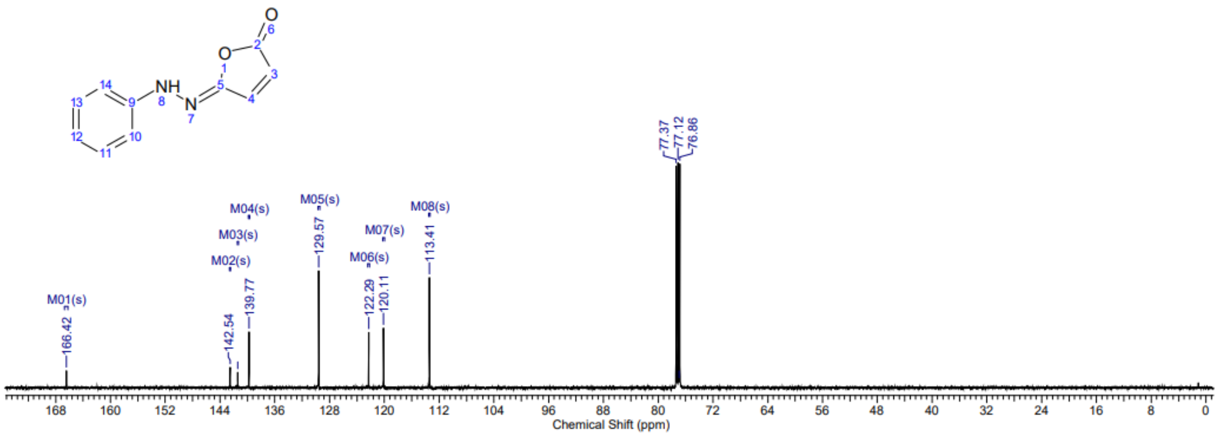

## **2.14.3.** LCMS

| No. | tR (min) | Peak Area (Y units*min) | Area (%) |
|---|---|---|---|
| 1 | 3.501 | 3752.794 | 0.057 |
| 2 | 3.558 | 985.523 | 0.015 |
| 3 | 3.692 | 12786.192 | 0.194 |
| 4 | 3.701 | 6817.278 | 0.104 |
| 5 | 4.791 | 2193.344 | 0.033 |
| 6 | 4.850 | 1708.168 | 0.026 |
| 7 | 4.898 | 1646.208 | 0.025 |
| 8 | 5.014 | 15022.431 | 0.228 |
| 9 | 5.026 | 2008.124 | 0.031 |
| 10 | 6.011 | 1015.560 | 0.015 |
| 11 | 6.041 | 5554.978 | 0.084 |
| 12 | 6.085 | 7171.559 | 0.109 |
| 13 | 6.202 | 1682.644 | 0.026 |
| 14 | 6.210 | 2585.633 | 0.039 |
| 15 | 6.749 | 6493140.500 | 98.726 |
| 16 | 7.027 | 3351.924 | 0.051 |
| 17 | 7.247 | 1253.181 | 0.019 |
| 18 | 7.261 | 1260.272 | 0.019 |
| 19 | 7.267 | 7169.134 | 0.109 |
| 20 | 7.278 | 3748.772 | 0.057 |
| 21 | 7.290 | 1938.826 | 0.029 |

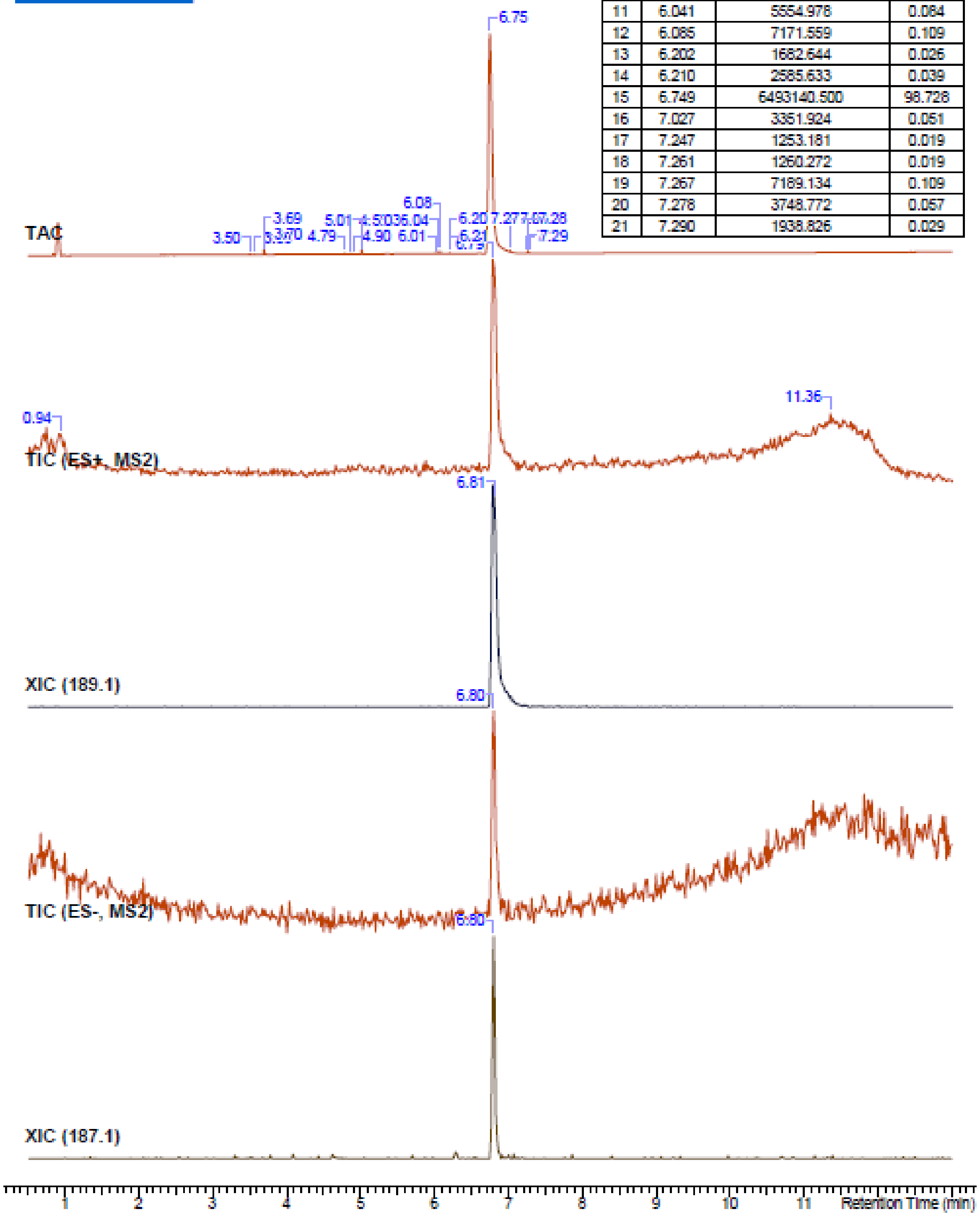

| tR (min) | Mass (Ao) | [M+H]+ | [M-H]- | Structure | Area (%) |
|---|---|---|---|---|---|
| 6.749 | 188.059 | 189.066 | 187.051 | 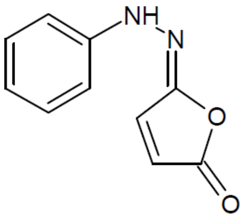 | TIC (ES-, MS2) = 100.00<br>XIC = 72.61<br>TIC (ES+, MS2) = 80.71<br>XIC = 69.61<br>TAC = 98.73 |

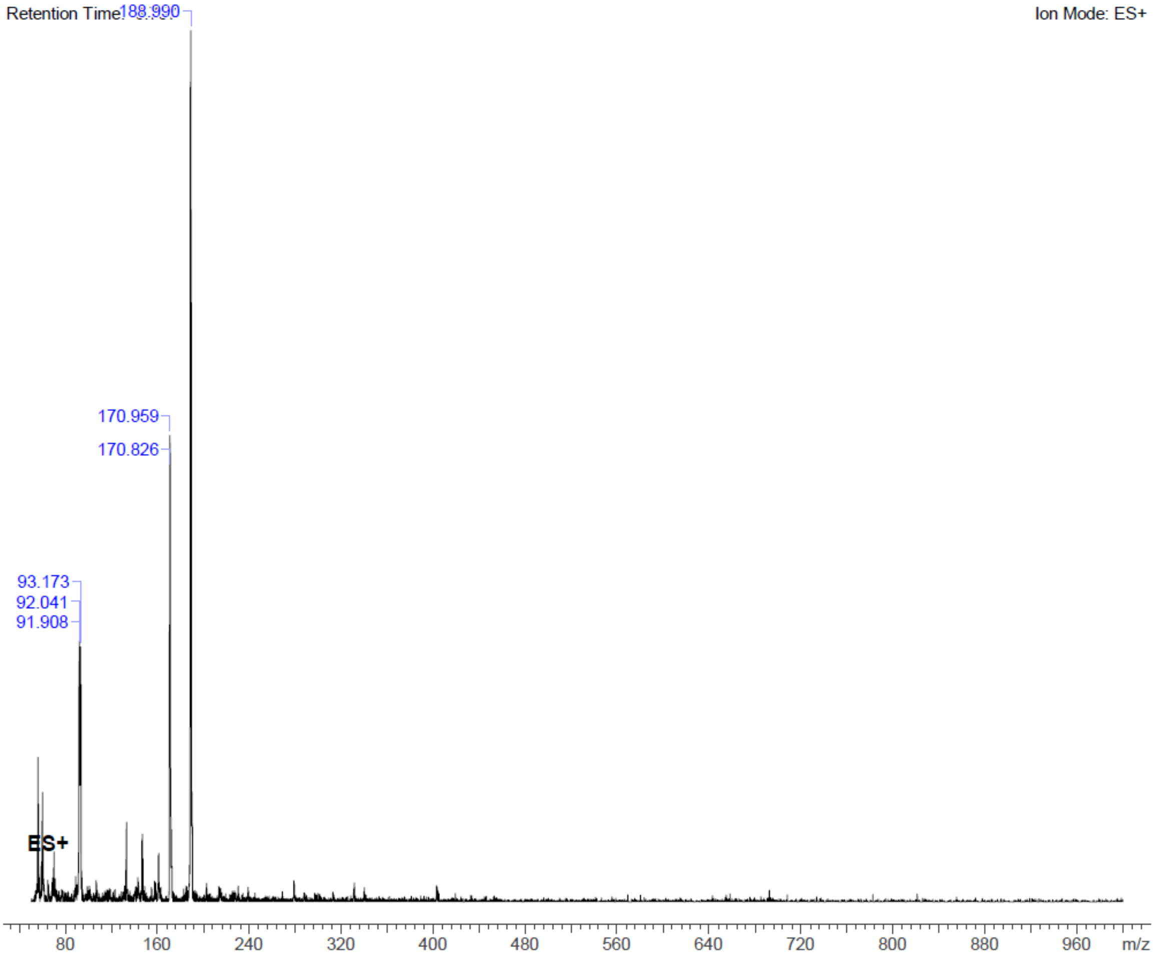

## 2.15. N'-[5-oxo-2,5-dihydrofuran-2-ylidene]thiophene-2-carbohydrazide (2h)

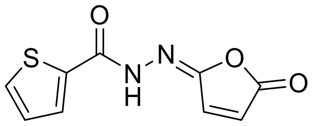

### 2.15.1. $^{1}$H NMR

05.12.2025 01:36:44

| Acquisition Time (sec) | 2.0479 | Date | 02 Dec 2025 12:02:31 | | | Date Stamp | 02 Dec 2025 12:01:52 |
|---|---|---|---|---|---|---|---|
| File Name | C:\Users\Admin\OneDrive - Uniwersytet Jagielloński\nmr\2025\izomal-tiof_935_03_PROTON-1-1.jdf | | | | | Frequency (MHz) | 500.16 |
| Nucleus | 1H | Number of Transients | 8 | Origin | ECA | Original Points Count | 19225 |
| Owner | delta | Points Count | 131072 | Pulse Sequence | single_pulse.jxp | Solvent | CHLOROFORM-d |
| Spectrum Offset (Hz) | 3251.0396 | Spectrum Type | STANDARD | Sweep Width (Hz) | 9387.80 | Temperature (degree C) | 20.800 |

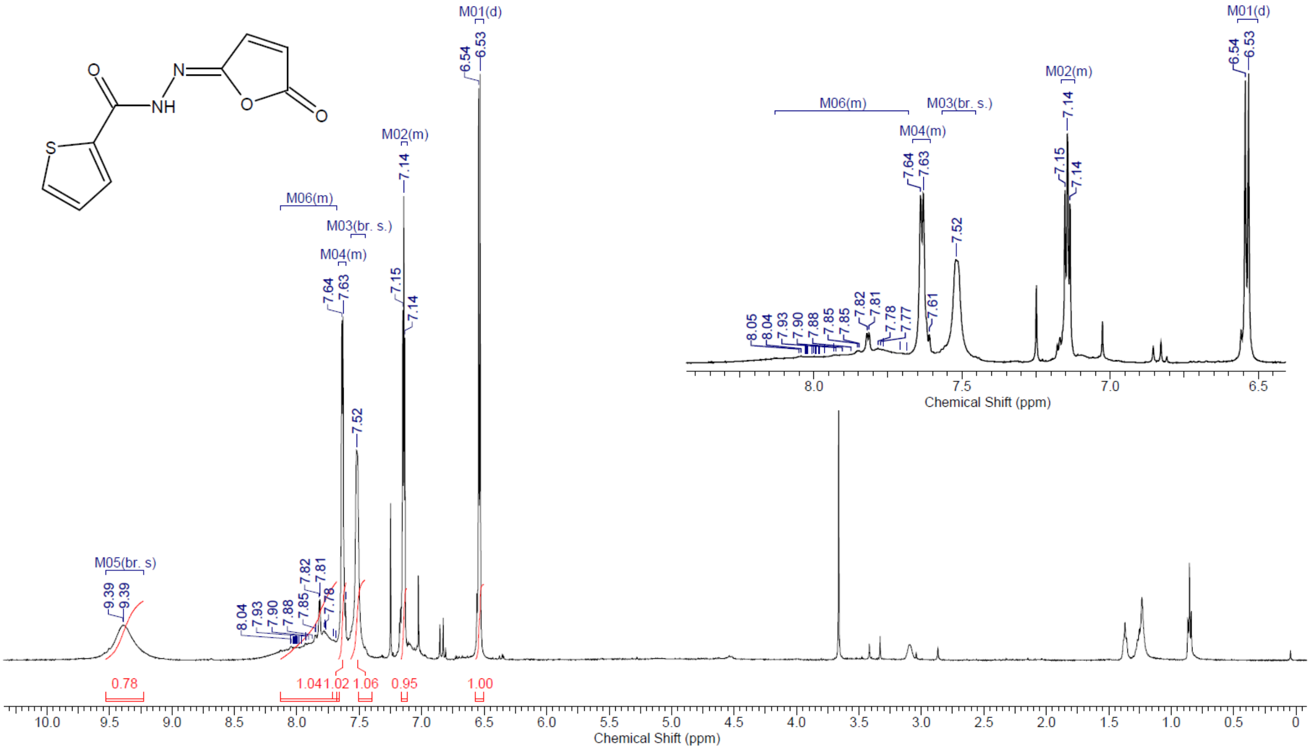

## 2.15.2. UPLC-MS

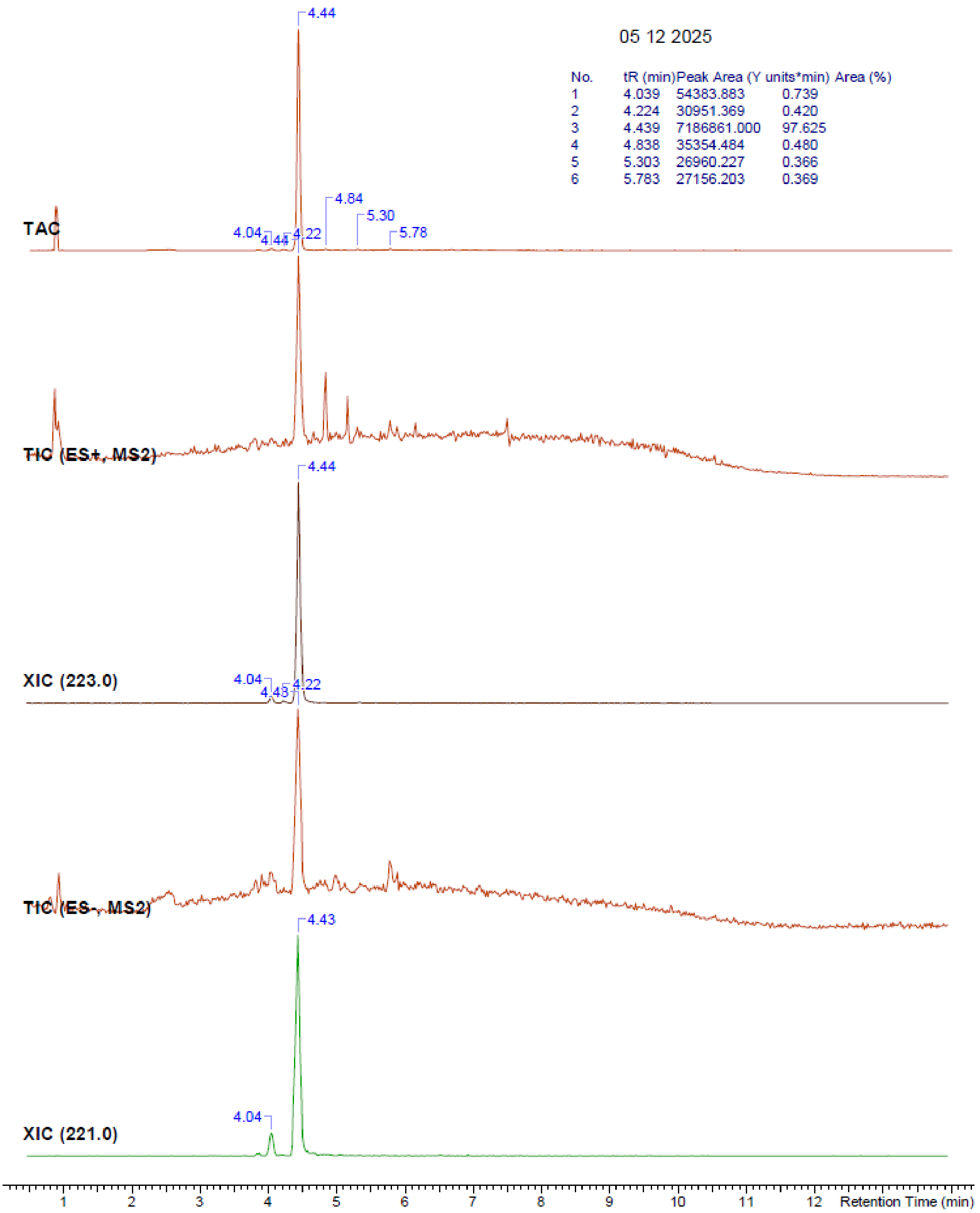


| Comp | tR(min) | Mass(Ao) | [M+H]+ | [M-H]- | M+· | M-· | MF | Structure | Area (%) |
|---|---|---|---|---|---|---|---|---|---|
| 1 | 4.440 | 222.010 | 223.017 | 221.003 | 222.009 | 222.010 | $C_9H_6N_2O_3S$ | 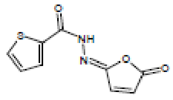 | TIC (ES-, MS2) = 100.00<br>Single XIC = 91.96<br>TIC (ES+, MS2) = 100.00<br>Single XIC = 96.78<br>TAC = 97.63 |

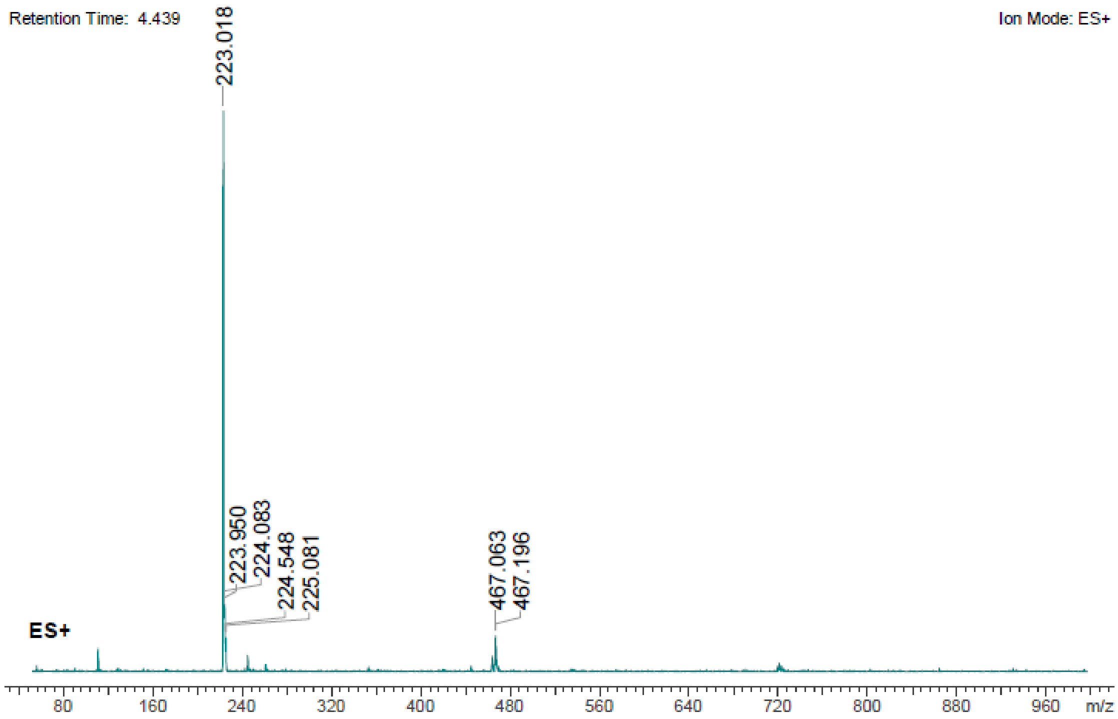
Retention Time: 4.439
Ion Mode: ES+
223.018
223.950
224.083
224.548
225.081
467.063
467.196
ES+
80
160
240
320
400
480
560
640
720
800
880
960
m/z

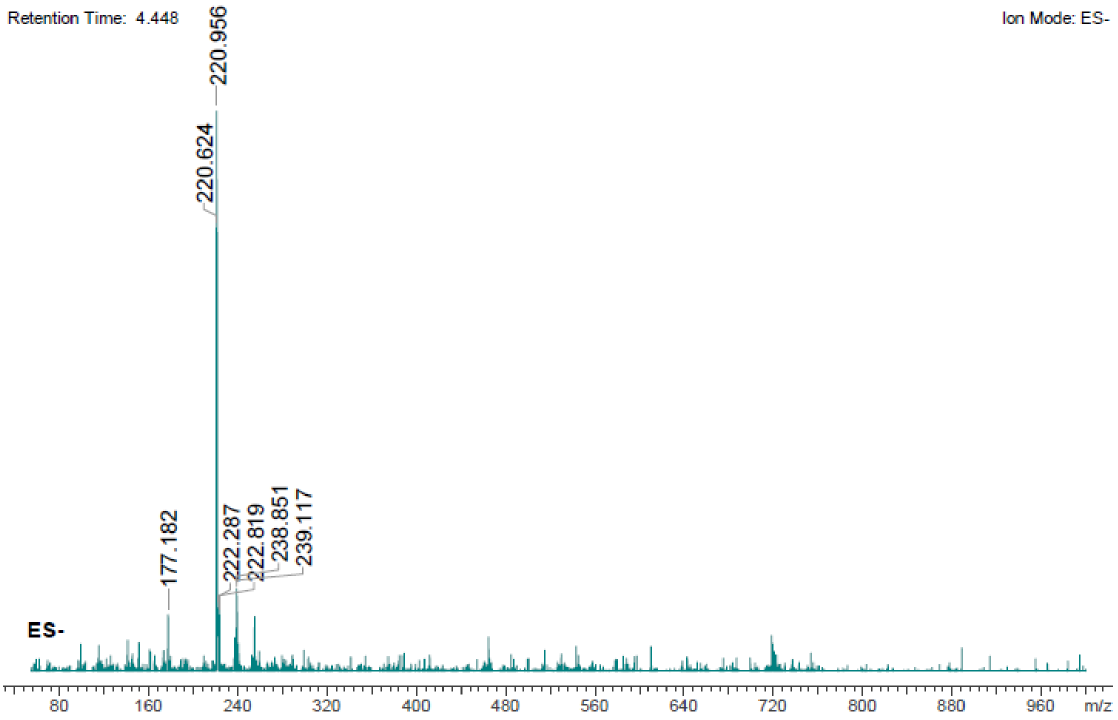
Retention Time: 4.448
Ion Mode: ES-
220.956
220.624
177.182
222.287
222.819
238.851
239.117
ES-
80
160
240
320
400
480
560
640
720
800
880
960
m/z

## 2.16. *N*-(2,5-Dioxo-2,5-dihydro-*1H*-pyrrol-1-yl)-4-(trifluoromethyl)benzamide (3a)

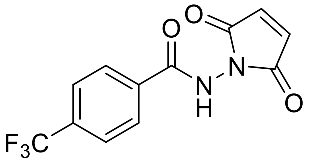

### 2.16.1. $^{1}$H NMR

| | | | | | | | |
|---|---|---|---|---|---|---|---|
| ***Acquisition Time (sec)*** | 2.0479 | ***Date*** | 12 Sep 2025 07:48:02 | | | ***Date Stamp*** | 12 Sep 2025 07:47:23 |
| ***File Name*** | C:\Users\panczykk\Desktop\Widma_KPS_PZ\NMR\2025\KPS25003_935_43_PROTON-1-1.jdf | | | | | ***Frequency (MHz)*** | 500.16 |
| ***Nucleus*** | 1H | ***Number of Transients*** | 8 | ***Origin*** | ECA | ***Original Points Count*** | 19225 |
| ***Owner*** | delta | ***Points Count*** | 131072 | ***Pulse Sequence*** | single_pulse.jxp | ***Solvent*** | DMSO-d6 |
| ***Spectrum Offset (Hz)*** | 3251.0396 | ***Spectrum Type*** | STANDARD | ***Sweep Width (Hz)*** | 9387.80 | ***Temperature (degree C)*** | 21.100 |

$^{1}$H NMR (500 MHz, DMSO-*d*$_6$) δ ppm 11.32 (s, 1H), 8.08 (d, *J*=8.17 Hz, 2H), 7.92 (d, *J*=8.31 Hz, 2H), 7.22 - 7.26 (m, 2H)

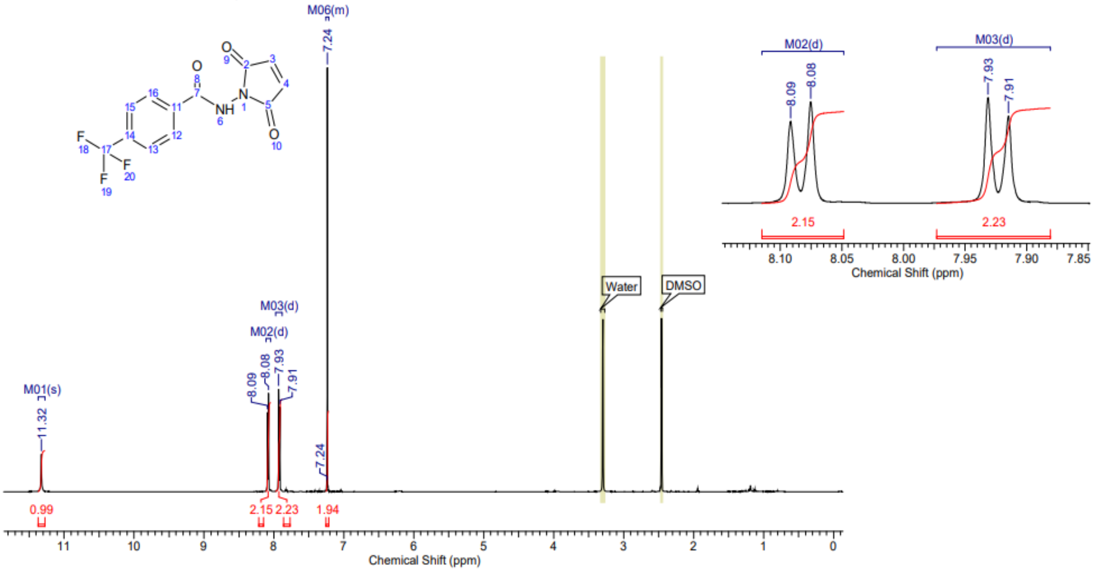

### 2.16.2. $^{13}$C NMR

| | | | | | | | |
|---|---|---|---|---|---|---|---|
| ***Acquisition Time (sec)*** | 1.9996 | ***Date*** | 12 Sep 2025 10:13:01 | | | ***Date Stamp*** | 12 Sep 2025 09:20:58 |
| ***File Name*** | C:\Users\panczykk\Desktop\Widma_KPS_PZ\NMR\2025\KPS25003_935_43_CARBON-1-1.jdf | | | | | ***Frequency (MHz)*** | 125.77 |
| ***Nucleus*** | 13C | ***Number of Transients*** | 1024 | ***Origin*** | ECA | ***Original Points Count*** | 78604 |
| ***Owner*** | delta | ***Points Count*** | 131072 | ***Pulse Sequence*** | carbon.jxp | ***Solvent*** | DMSO-d6 |
| ***Spectrum Offset (Hz)*** | 12576.5293 | ***Sweep Width (Hz)*** | 39310.18 | ***Temperature (degree C)*** | 21.500 | | |

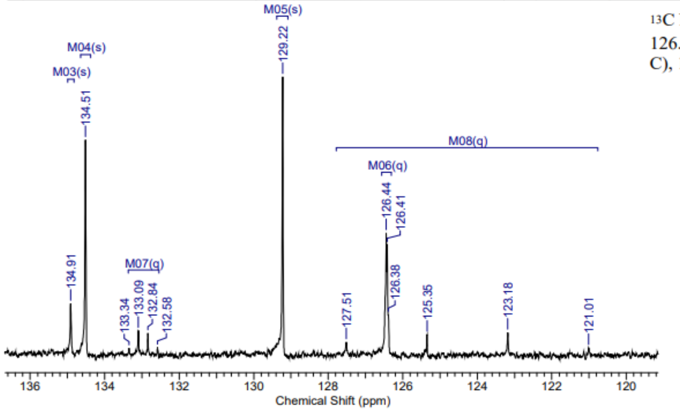

$^{13}$C NMR (126 MHz, DMSO-*d*$_6$) δ ppm 124.26 (q, *J*=272.00 Hz, 0 C), 126.43 (q, *J*=3.60 Hz, 1 C), 129.22 (s, 1 C), 132.96 (q, *J*=32.10 Hz, 1 C), 134.51 (s, 1 C), 134.91 (s, 1 C), 164.94 (s, 1 C), 168.64 (s, 1 C)

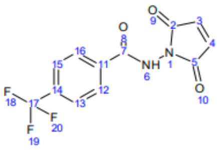

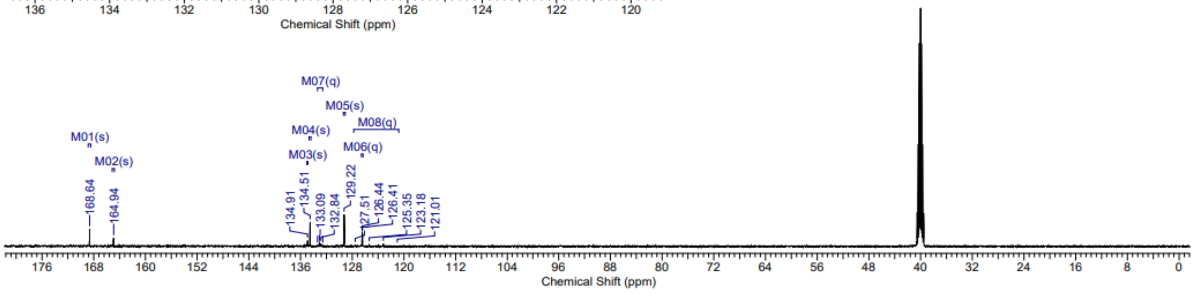

### 2.16.3. $^{19}$F NMR

| Acquisition Time (sec) | 2.9861 | Date | 26 Mar 2024 11:29:23 | | | Date Stamp | 26 Mar 2024 11:28:36 |
|---|---|---|---|---|---|---|---|
| File Name | C:\Users\Przemek\OneDrive - Uniwersytet Jagielloński\nmr\o2024\CF3-mal_935_03_Fluorine-1-1.jdf | | | | | Frequency (MHz) | 470.62 |
| Nucleus | 19F | Number of Transients | 8 | Origin | ECA | Original Points Count | 352967 |
| Owner | delta | Points Count | 524288 | Pulse Sequence | single_pulse.jxp | Solvent | DMSO-d6 |
| Spectrum Offset (Hz) | -47062.0469 | Spectrum Type | STANDARD | Sweep Width (Hz) | 118203.65 | Temperature (degree C) | 20.700 |

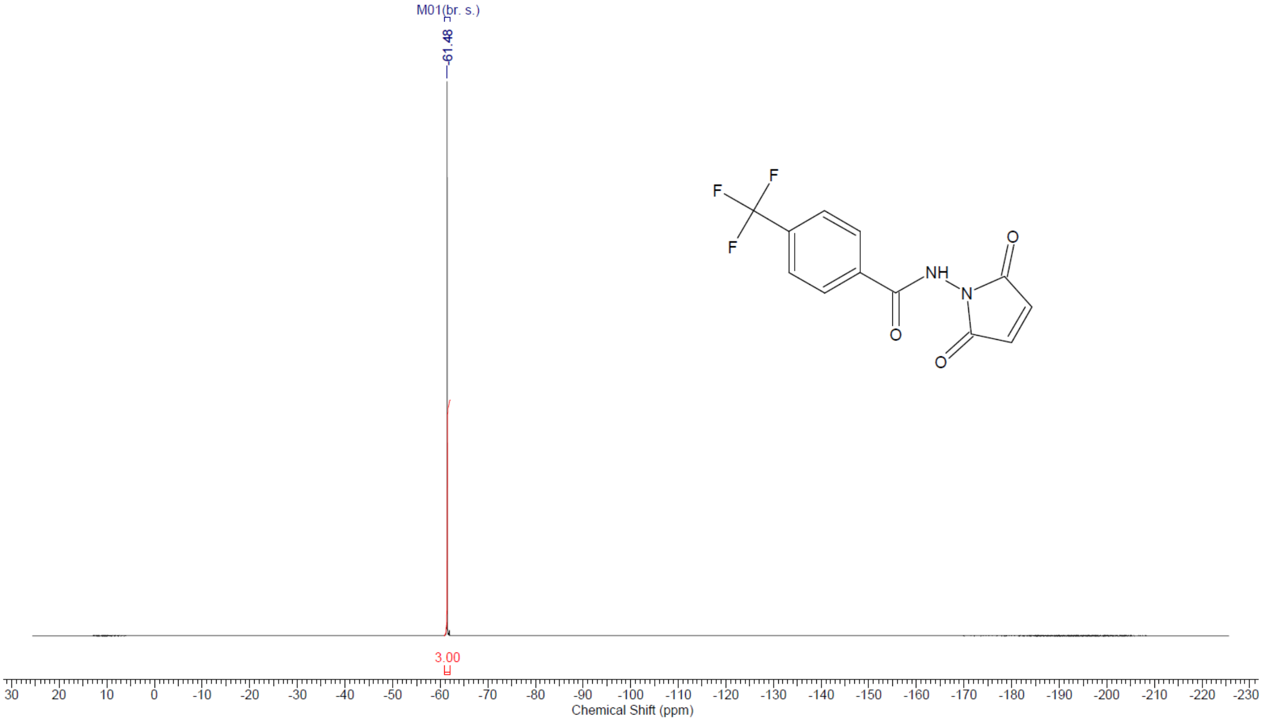

**2.16.4.** LCMS

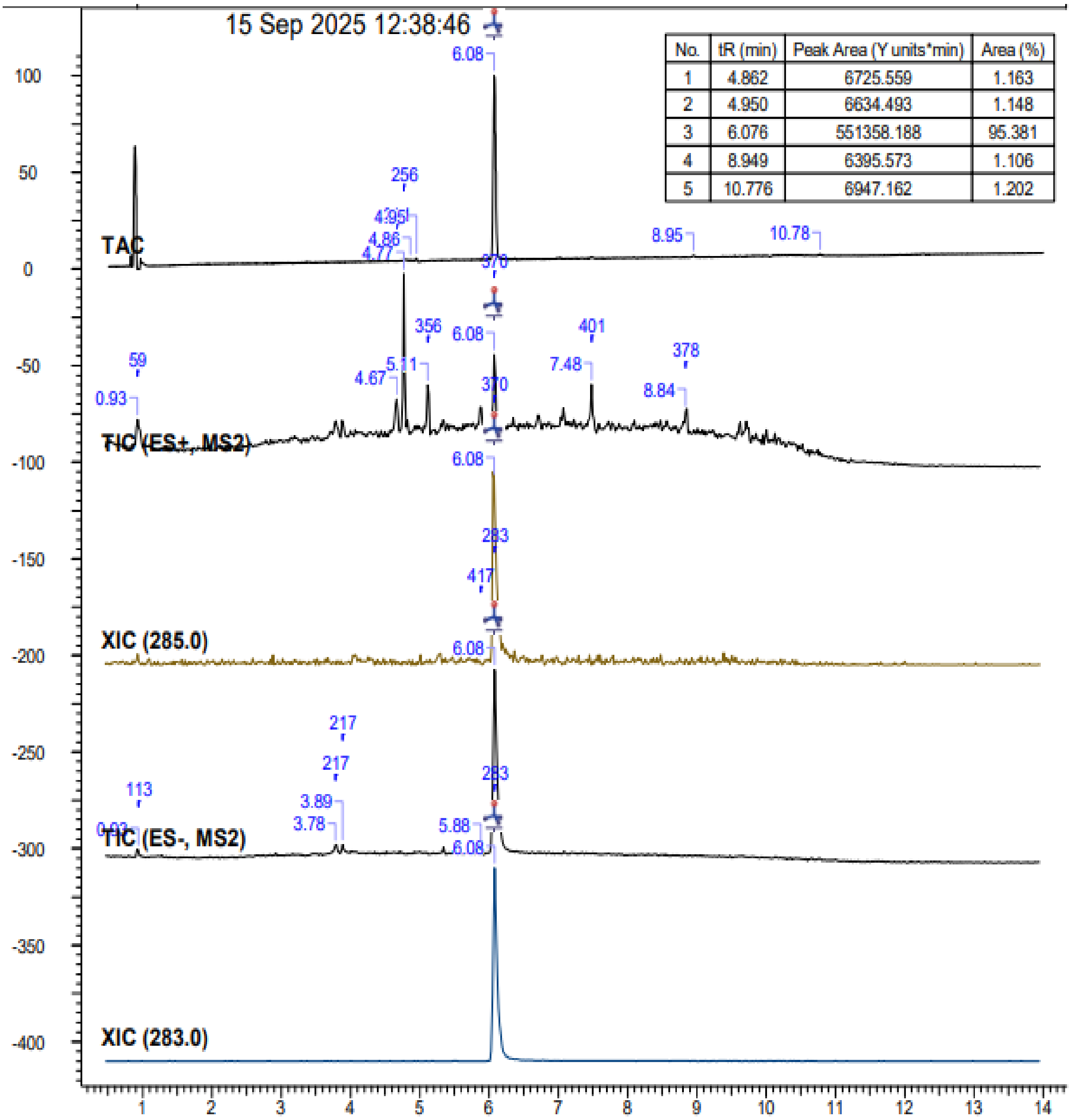


| No. | tR (min) | Peak Area (Y units*min) | Area (%) |
|---|---|---|---|
| 1 | 4.862 | 6725.559 | 1.163 |
| 2 | 4.950 | 6634.493 | 1.148 |
| 3 | 6.076 | 551358.188 | 95.381 |
| 4 | 8.949 | 6395.573 | 1.106 |
| 5 | 10.776 | 6947.162 | 1.202 |

| No. | tR(min) | Mass(Ao) | [M+H]+ | [M-H]- | MF | Structure | Name | Area (%) |
|---|---|---|---|---|---|---|---|---|
| 1 | 6.08 | 284.0409 | 285.0482 | 283.0336 | $C_{12}H_7F_3N_2O_3$ | 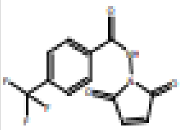 | | TIC (ES-, MS2) = 89.94<br>Single XIC = 100.00<br>TIC (ES+, MS2) = 15.54<br>Single XIC = 100.00<br>TAC = 95.38 |

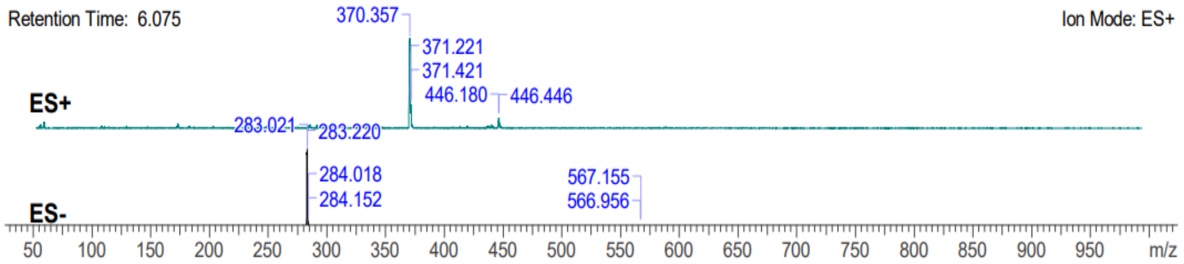
Retention Time: 6.075
Ion Mode: ES+
ES+
370.357
371.221
371.421
446.180
446.446
283.021
283.220
ES-
284.018
284.152
567.155
566.956
50
100
150
200
250
300
350
400
450
500
550
600
650
700
750
800
850
900
950
m/z

## 2.17. *Tert*-butyl (2,5-dioxo-2,5-dihydro-*1H*-pyrrol-1-yl)carbamate (3b)

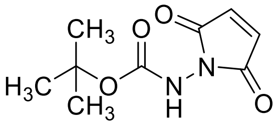


### 2.17.1. $^{1}$H NMR

| Acquisition Time (sec) | 1.9984 | Date | 06 Feb 2024 10:10:22 |
|---|---|---|---|
| Date Stamp | 06 Feb 2024 10:09:44 | | |
| File Name | \\AppServerM9\Wyniki_NMR_43_\2024\KPS24-8-fr-14-22_935_43_PROTON-1-1.jdf | | |
| Frequency (MHz) | 500.1599 | Nucleus | 1H |
| Number of Transients | 8 | Origin | ECA |
| Original Points Count | 18757 | Owner | delta |
| Points Count | 131072 | Pulse Sequence | single_pulse.jxp |
| Solvent | DMSO-d6 | Spectrum Offset (Hz) | 3251.0396 |
| Spectrum Type | STANDARD | Sweep Width (Hz) | 9385.89 |
| Temperature (degree C) | 20.300 | | |

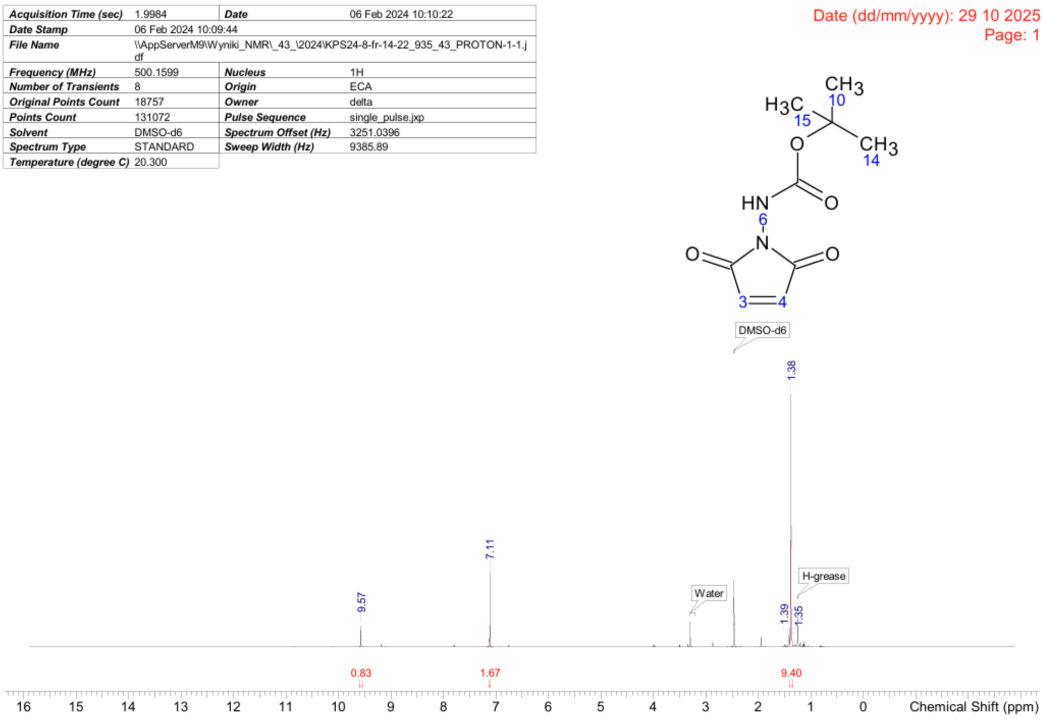


### 2.17.2. $^{13}$C NMR

| Acquisition Time (sec) | 1.9996 | Date | 06 Feb 2024 11:02:55 |
|---|---|---|---|
| Date Stamp | 06 Feb 2024 10:10:52 | | |
| File Name | \\AppServerM9\Wyniki_NMR_43_\2024\KPS24-8-fr-14-22_935_43_CARBON-1-1.jdf | | |
| Frequency (MHz) | 125.7653 | Nucleus | 13C |
| Number of Transients | 1024 | Origin | ECA |
| Original Points Count | 78604 | Owner | delta |
| Points Count | 131072 | Pulse Sequence | carbon.jxp |
| Solvent | DMSO-d6 | Spectrum Offset (Hz) | 12576.5293 |
| Sweep Width (Hz) | 39310.18 | Temperature (degree C) | 20.300 |

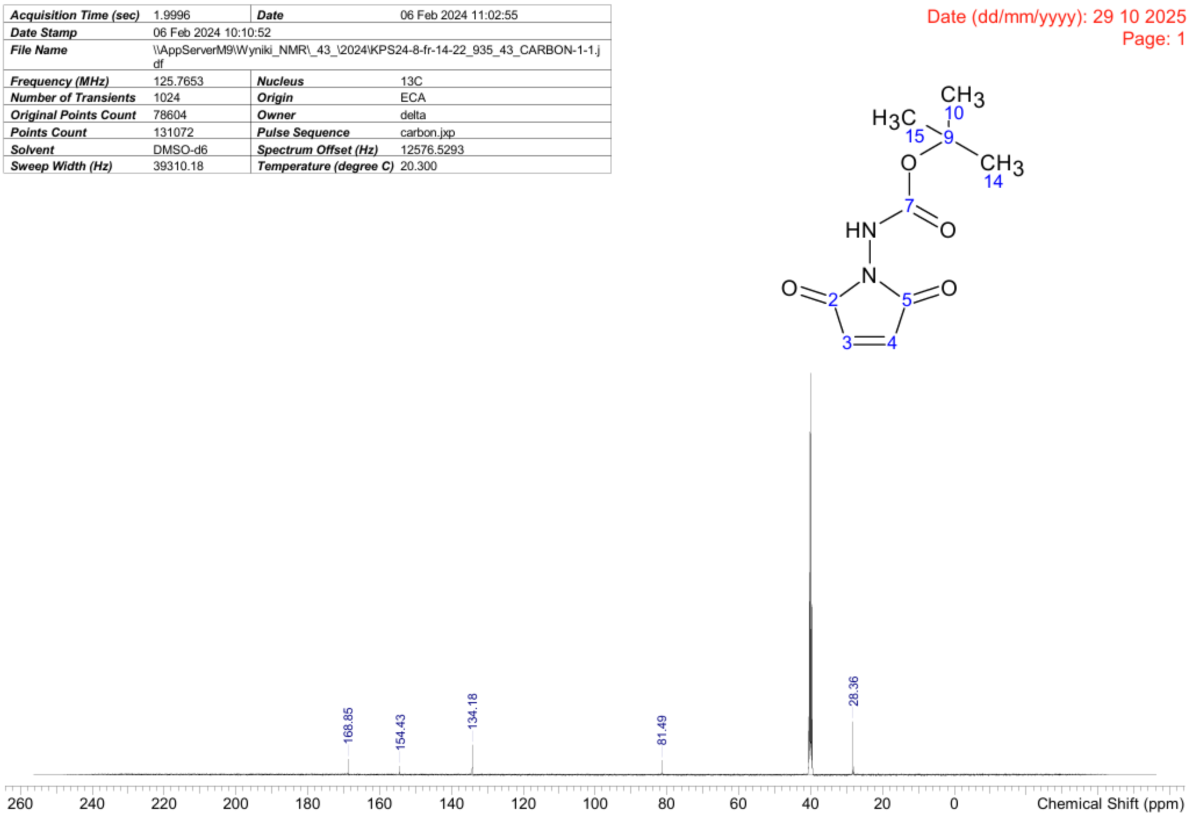

### **2.17.3.** LCMS

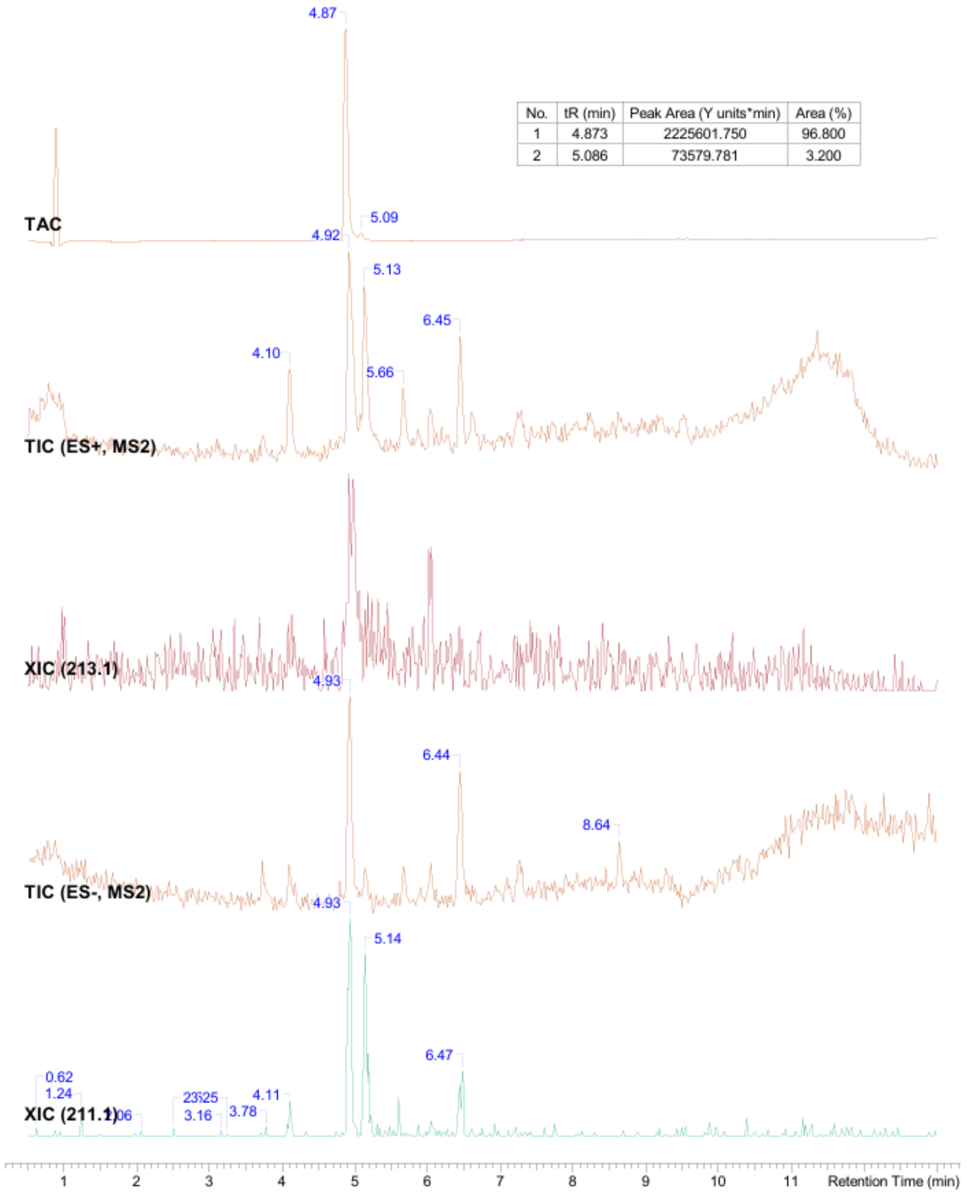


| Compoı | tR(min) | Mass(Ao) | [M+H]+ | [M-H]- | M+· | M-· | MF | Structure | Area (%) |
|---|---|---|---|---|---|---|---|---|---|
| 1 | 4.873 | 212.080 | 213.087 | 211.072 | 212.079 | 212.080 | $C_9H_{12}N_2O_4$ | 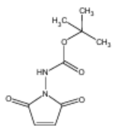 | TIC (ES-, MS2) = 49.86 Single XIC = 42.95 TIC (ES+, MS2) = 34.81 TAC = 96.80 |

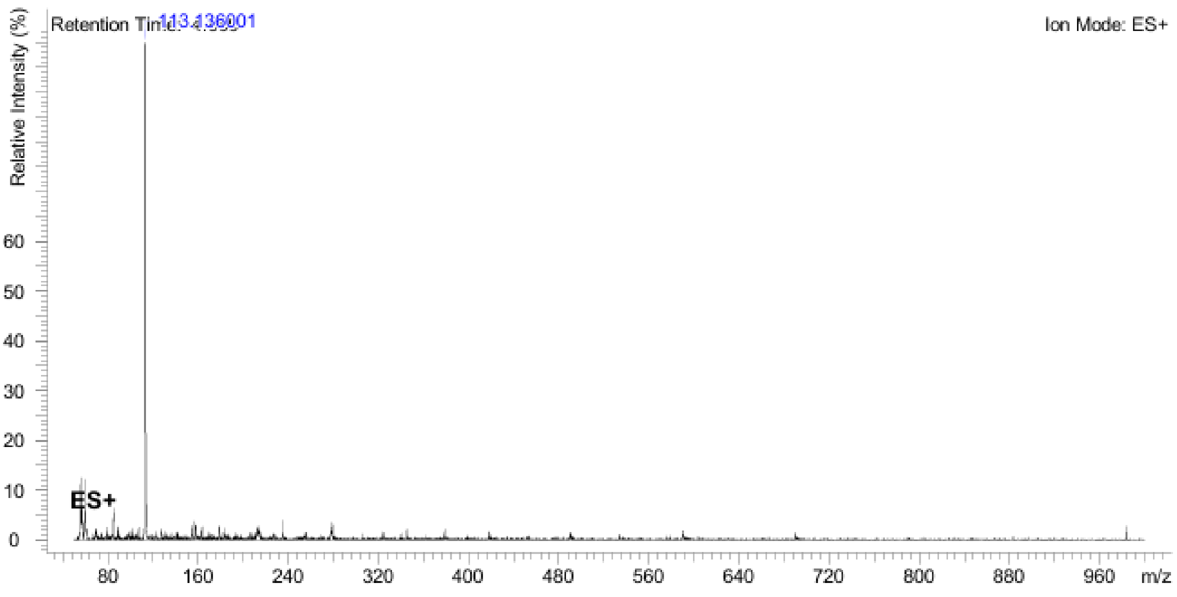


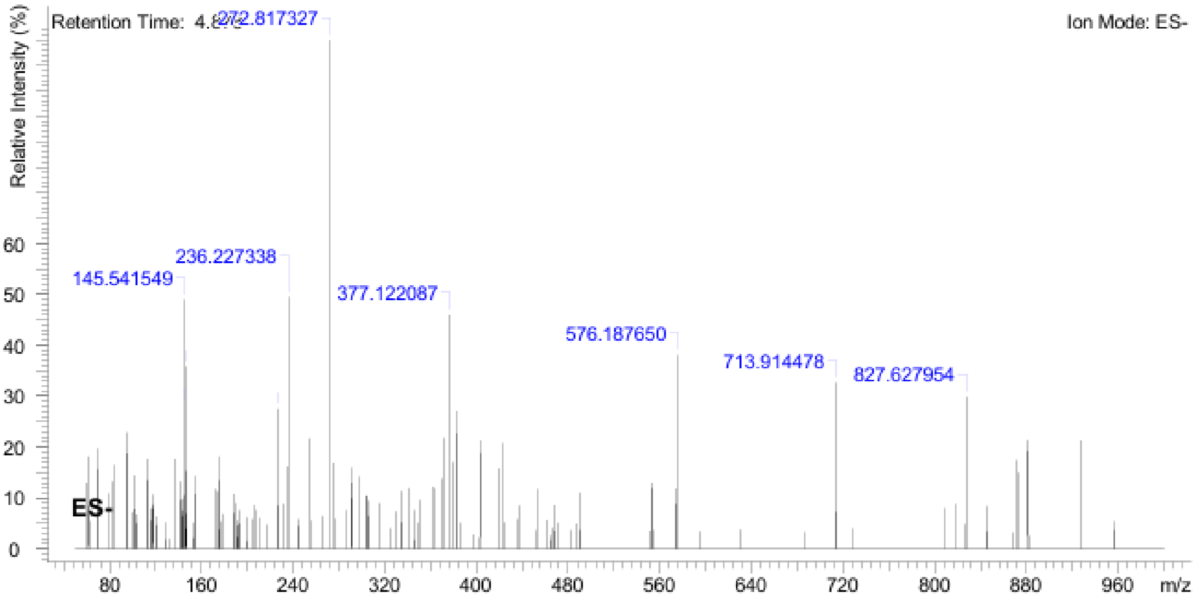


**2.17.4.** Data before purification by column chromatography

$^{1}$H NMR (500 MHz, DMSO-$d_6$) δ (ppm) 1.38 (s, 9H, $CH_3$) 7.11 (s, 2H, >CH-) 9.57 (s, 1H, NH)

| | | | |
|---|---|---|---|
| ***Acquisition Time (sec)*** | 4.3647 | ***Date*** | 06 Feb 2024 09:12:17 |
| ***Date Stamp*** | 06 Feb 2024 09:11:23 | | |
| ***File Name*** | \\AppServerM9\Wyniki_NMR_43_\2024\KPS24-8_935_43_PROTON-1-1.jdf | | |
| ***Frequency (MHz)*** | 500.1599 | ***Nucleus*** | 1H |
| ***Number of Transients*** | 8 | ***Origin*** | ECA |
| ***Original Points Count*** | 40960 | ***Owner*** | delta |
| ***Points Count*** | 131072 | ***Pulse Sequence*** | single_pulse.jxp |
| ***Solvent*** | DMSO-d6 | ***Spectrum Offset (Hz)*** | 3251.0396 |
| ***Spectrum Type*** | STANDARD | ***Sweep Width (Hz)*** | 9384.38 |
| ***Temperature (degree C)*** | 20.100 | | |

Date (dd/mm/yyyy): 28 10 2025
Page: 1

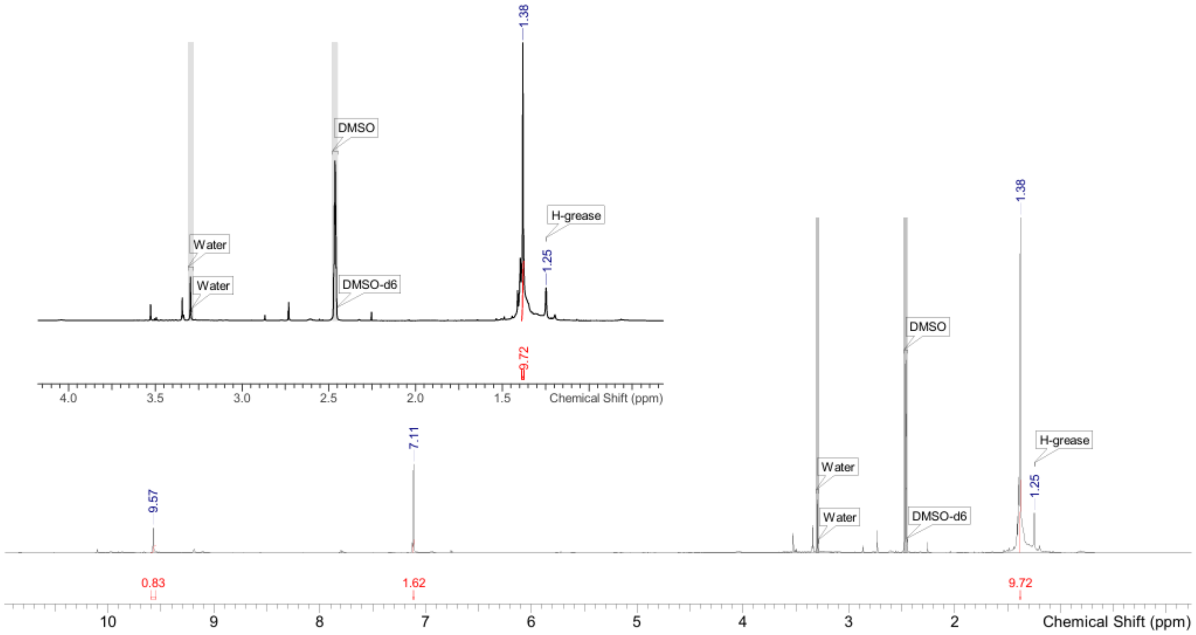

## 2.18. *N*-(2,5-Dioxo-2,5-dihydro-*1H*-pyrrol-1-yl)benzamide (3c)

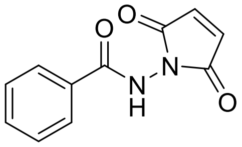

### 2.18.1. $^{1}$H NMR

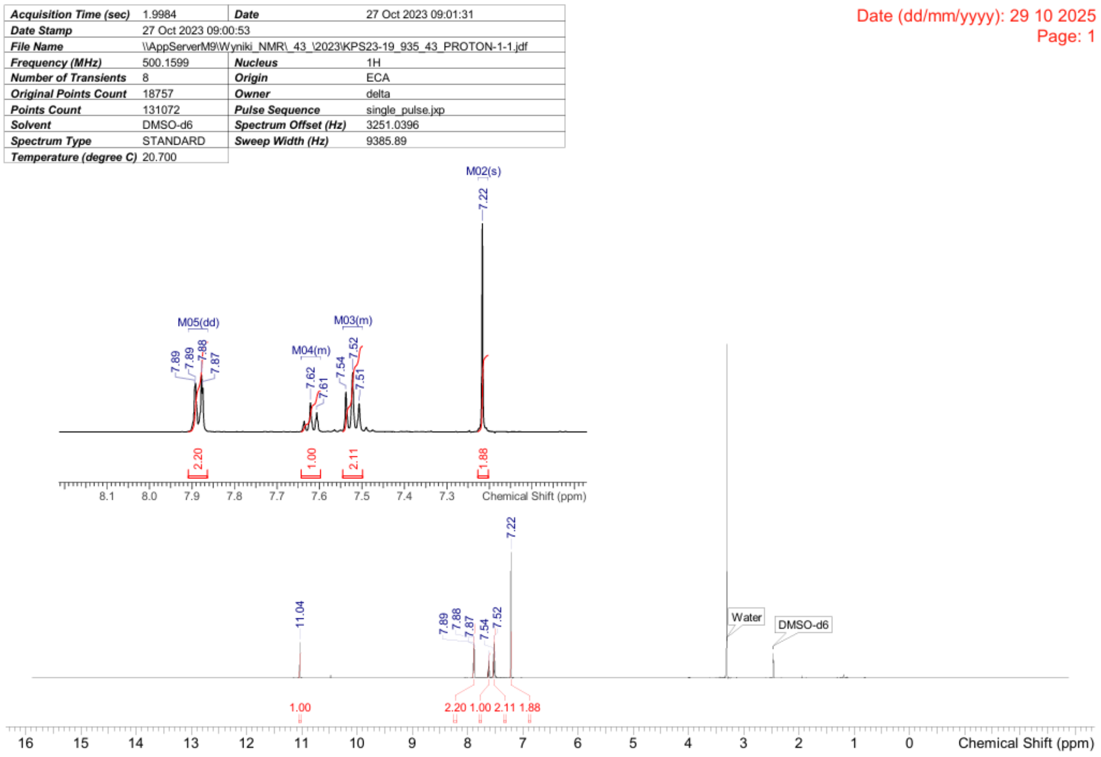

| | | | |
|---|---|---|---|
| Acquisition Time (sec) | 1.9984 | Date | 27 Oct 2023 09:01:31 |
| Date Stamp | 27 Oct 2023 09:00:53 | | |
| File Name | \\AppServerM9\Wyniki_NMR_43_\2023\KPS23-19_935_43_PROTON-1-1.jdf | | |
| Frequency (MHz) | 500.1599 | Nucleus | 1H |
| Number of Transients | 8 | Origin | ECA |
| Original Points Count | 18757 | Owner | delta |
| Points Count | 131072 | Pulse Sequence | single_pulse.jxp |
| Solvent | DMSO-d6 | Spectrum Offset (Hz) | 3251.0396 |
| Spectrum Type | STANDARD | Sweep Width (Hz) | 9385.89 |
| Temperature (degree C) | 20.700 | | |



### 2.18.2. $^{13}$C NMR

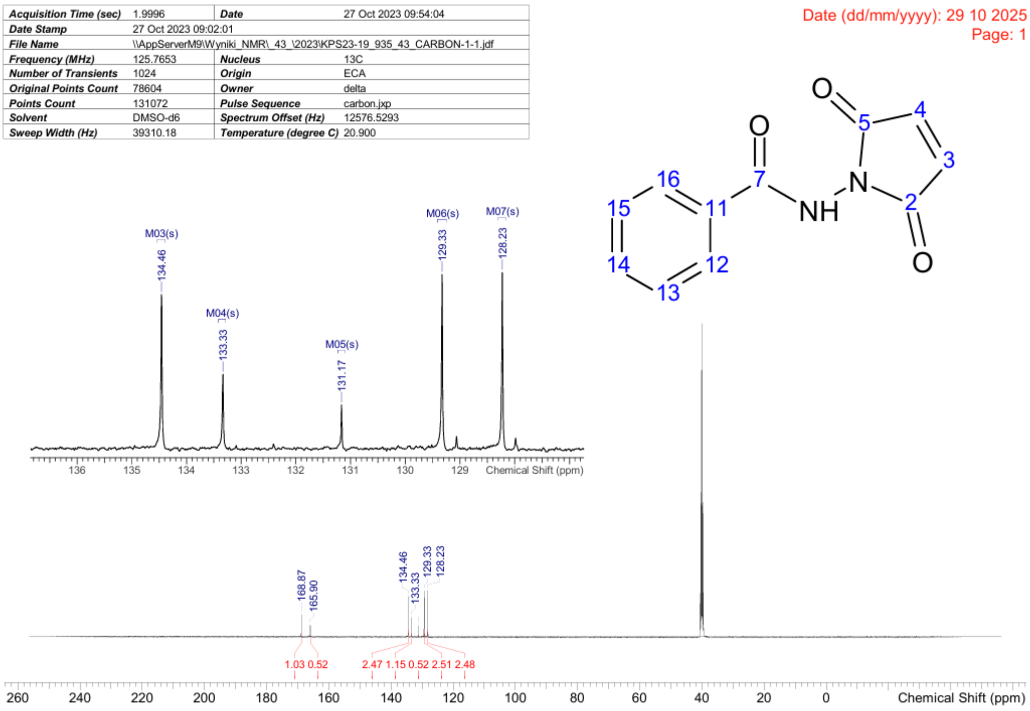

| | | | |
|---|---|---|---|
| Acquisition Time (sec) | 1.9996 | Date | 27 Oct 2023 09:54:04 |
| Date Stamp | 27 Oct 2023 09:02:01 | | |
| File Name | \\AppServerM9\Wyniki_NMR_43_\2023\KPS23-19_935_43_CARBON-1-1.jdf | | |
| Frequency (MHz) | 125.7653 | Nucleus | 13C |
| Number of Transients | 1024 | Origin | ECA |
| Original Points Count | 78604 | Owner | delta |
| Points Count | 131072 | Pulse Sequence | carbon.jxp |
| Solvent | DMSO-d6 | Spectrum Offset (Hz) | 12576.5293 |
| Sweep Width (Hz) | 39310.18 | Temperature (degree C) | 20.900 |

**2.18.3.** LCMS

Chemistry
Development

| No. | tR (min) | Peak Area (Y units*min) | Area (%) |
|---|---|---|---|
| 1 | 3.040 | 6107.761 | 0.150 |
| 2 | 4.202 | 3876196.750 | 95.223 |
| 3 | 4.378 | 118048.672 | 2.900 |
| 4 | 4.519 | 9611.770 | 0.236 |
| 5 | 5.414 | 5105.151 | 0.125 |
| 6 | 6.115 | 12195.104 | 0.300 |
| 7 | 7.177 | 4419.718 | 0.109 |
| 8 | 11.573 | 38956.371 | 0.957 |

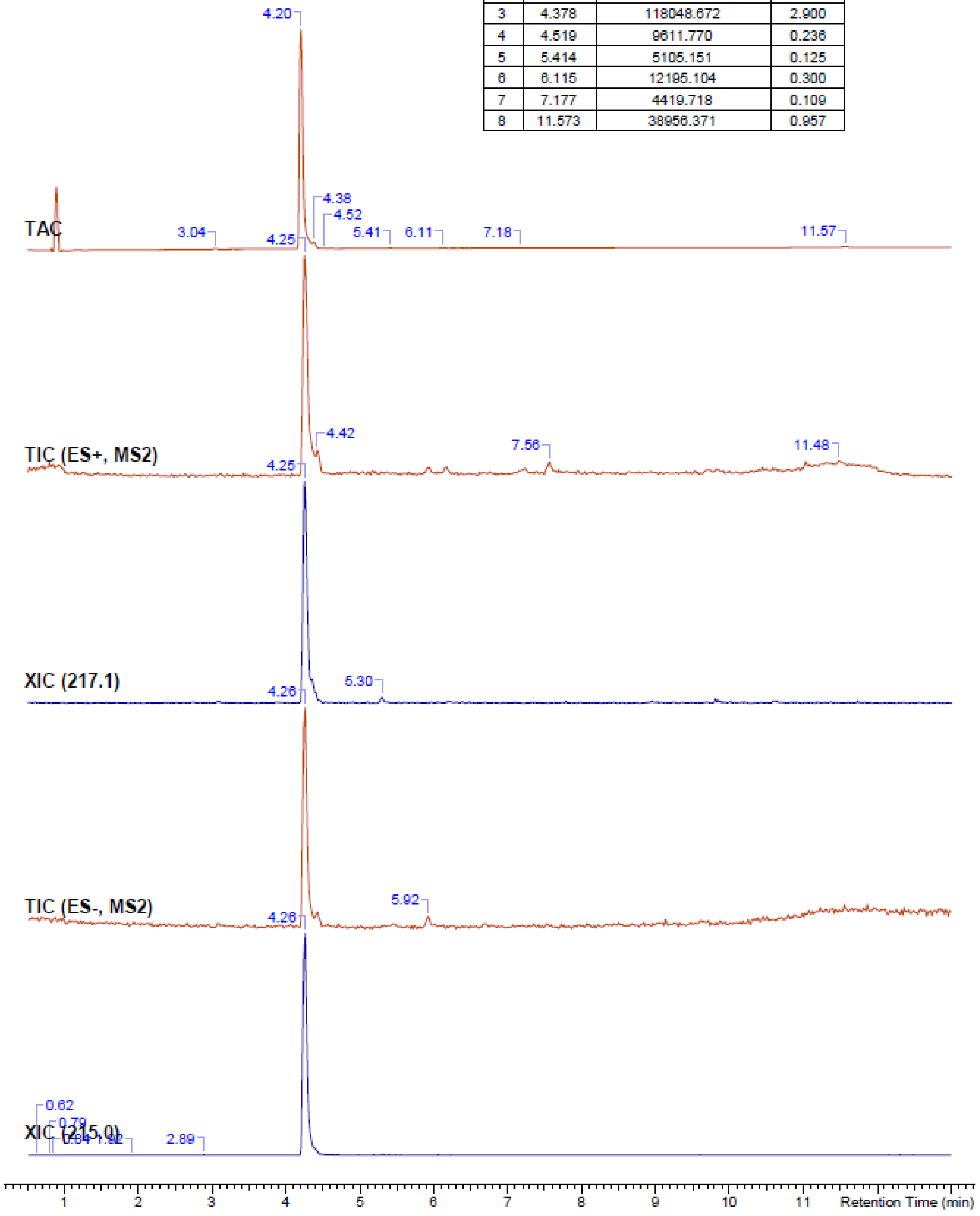


| tR (min) | Mass (Ao) | [M+H]+ | [M-H]- | Area (%) |
|---|---|---|---|---|
| 4.202 | 216.053 | 217.061 | 215.046 | TIC (ES-, MS2) = 93.99<br>XIC = 89.86<br>TIC (ES+, MS2) = 81.15<br>XIC = 59.03<br>TAC = 95.22 |

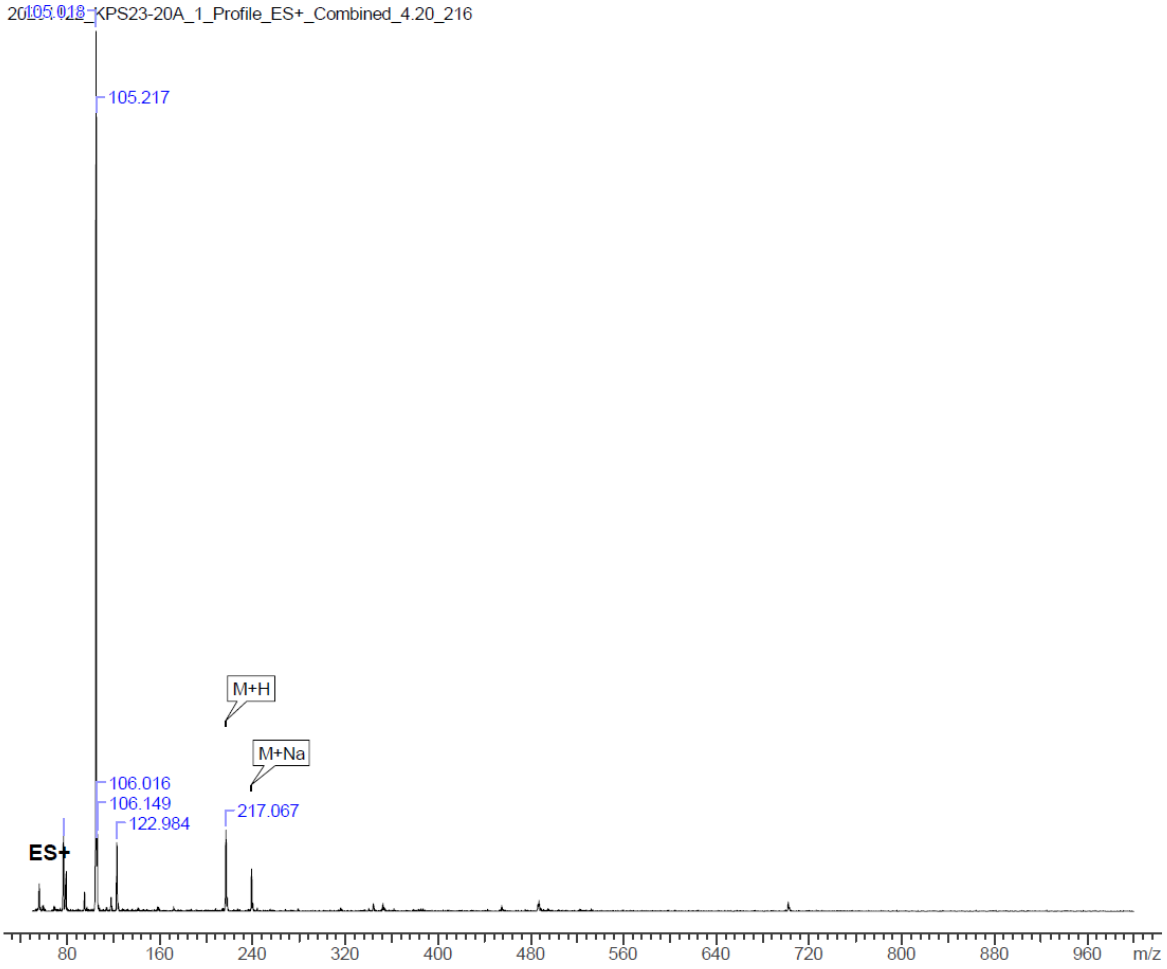
20105.018_KPS23-20A_1_Profile_ES+_Combined_4.20_216
105.217
M+H
M+Na
106.016
106.149
122.984
217.067
ES+
80
160
240
320
400
480
560
640
720
800
880
960
m/z

## 2.19.4-(Dimethylamino)-*N*-(2,5-dioxo-2,5-dihydro-*1H*-pyrrol-1-yl)benzamide (3d)

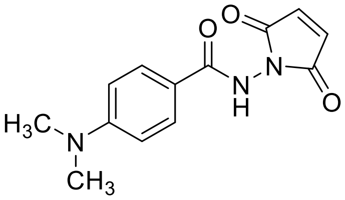

### 2.19.1. $^{1}$H NMR

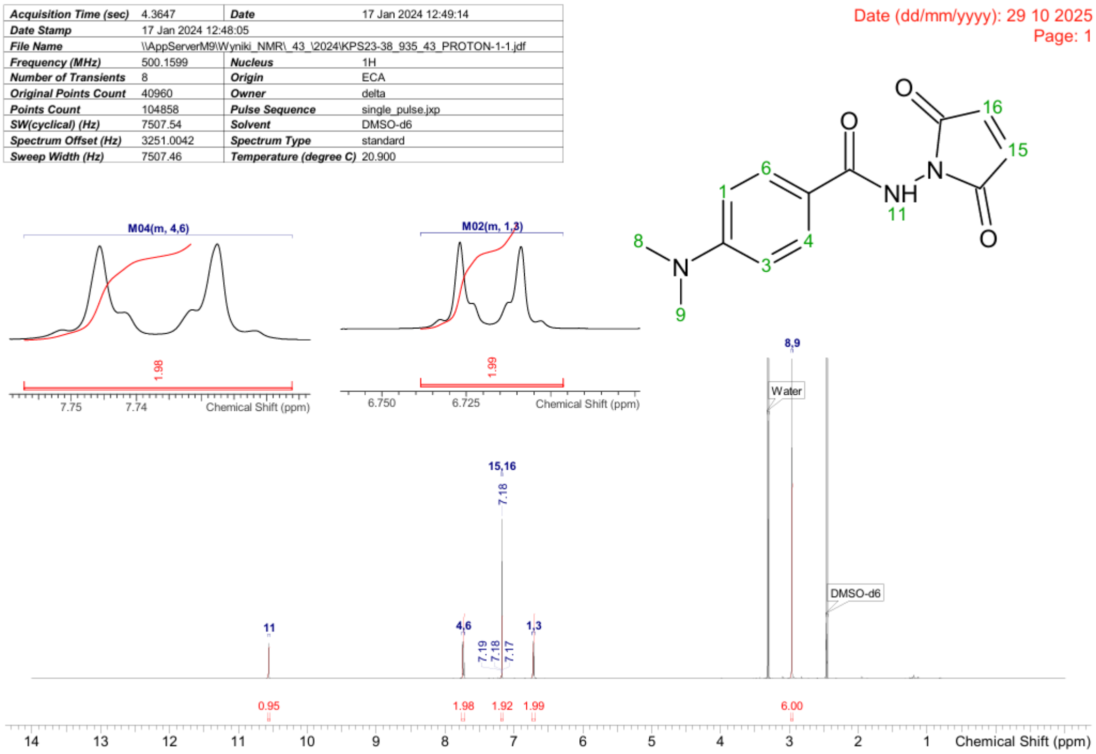


### 2.19.2. $^{13}$C NMR

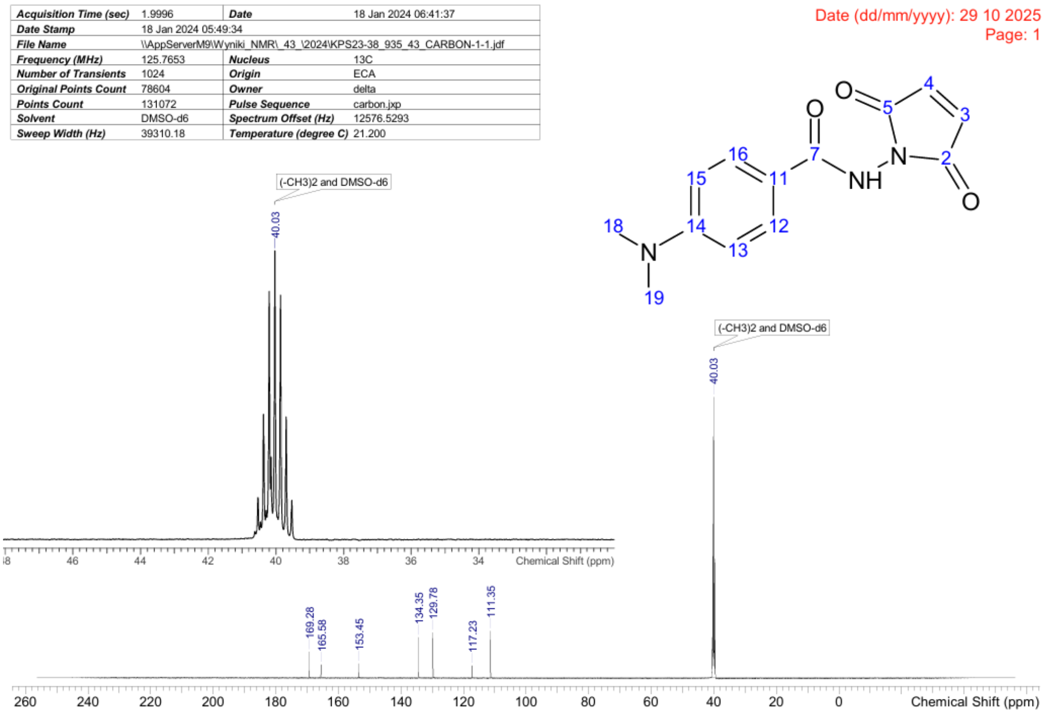

**2.19.3.** LCMS

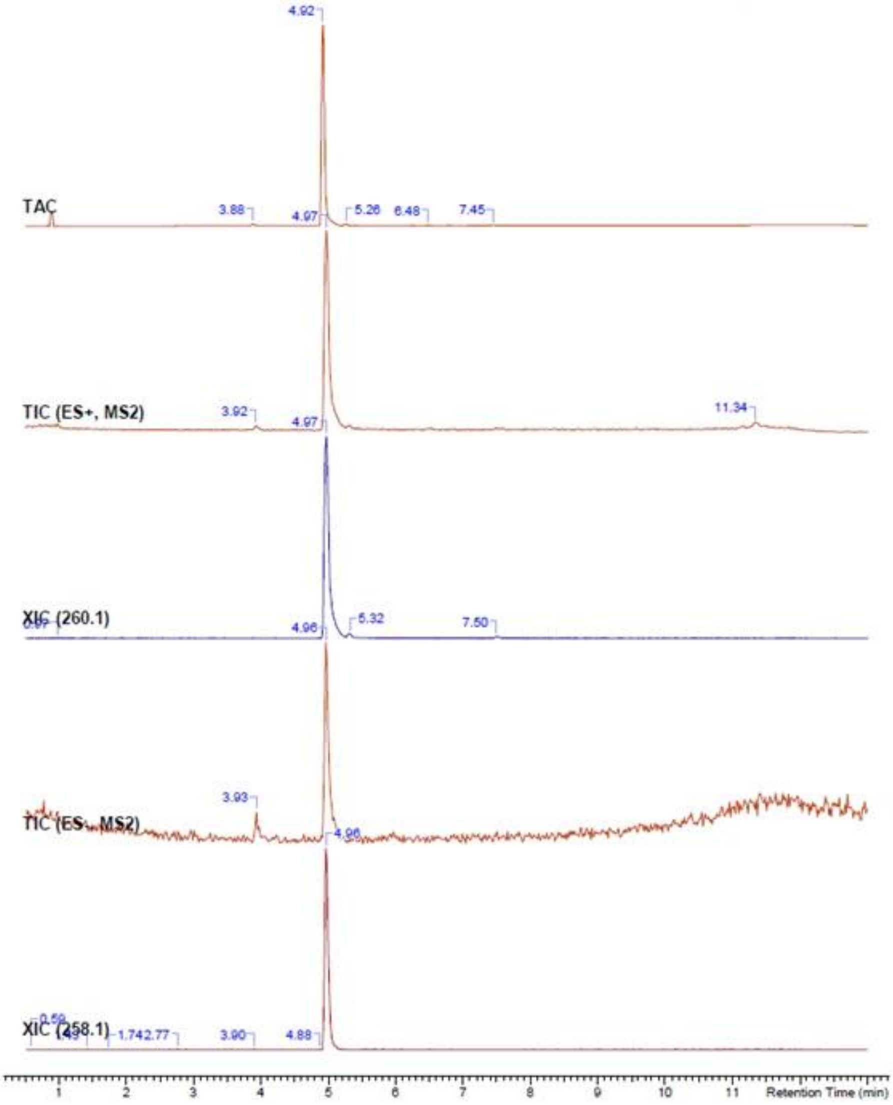


| tR (min) | Mass (Ao) | [M+H]+ | [M-H]- | Area (%) |
|---|---|---|---|---|
| 4.919 | 259.096 | 260.103 | 258.088 | TIC (ES-, MS2) = 91.98<br>XIC = 85.52<br>TIC (ES+, MS2) = 88.53<br>XIC = 77.93<br>TAC = 97.84 |

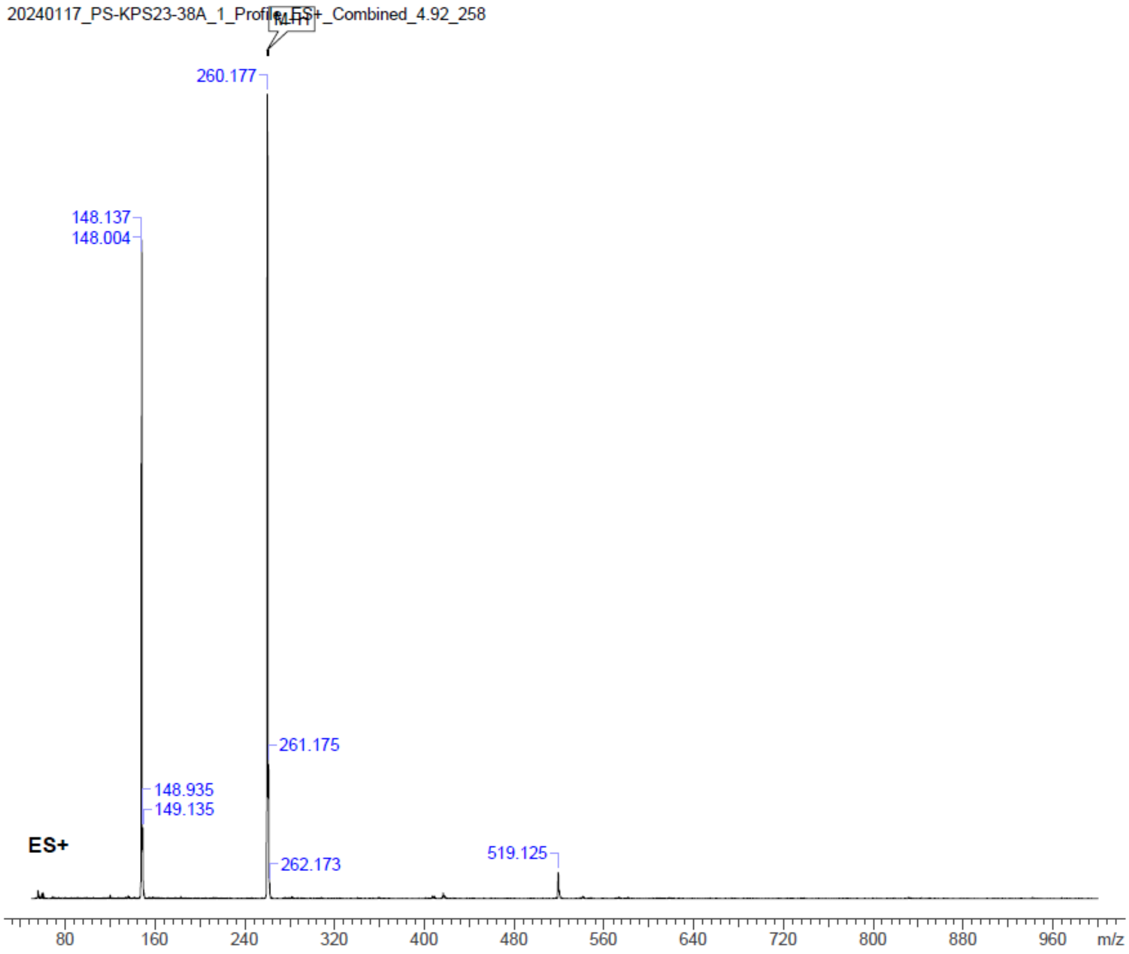
20240117_PS-KPS23-38A_1_Profile_ES+_Combined_4.92_258
M+H
260.177
148.137
148.004
261.175
148.935
149.135
ES+
262.173
519.125
80
160
240
320
400
480
560
640
720
800
880
960
m/z

## 2.20. *N*-(2,5-Dioxo-2,5-dihydro-*1H*-pyrrol-1-yl)-4-methoxybenzamide (3e)

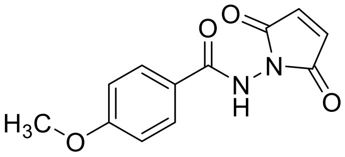

### 2.20.1. $^{1}$H NMR

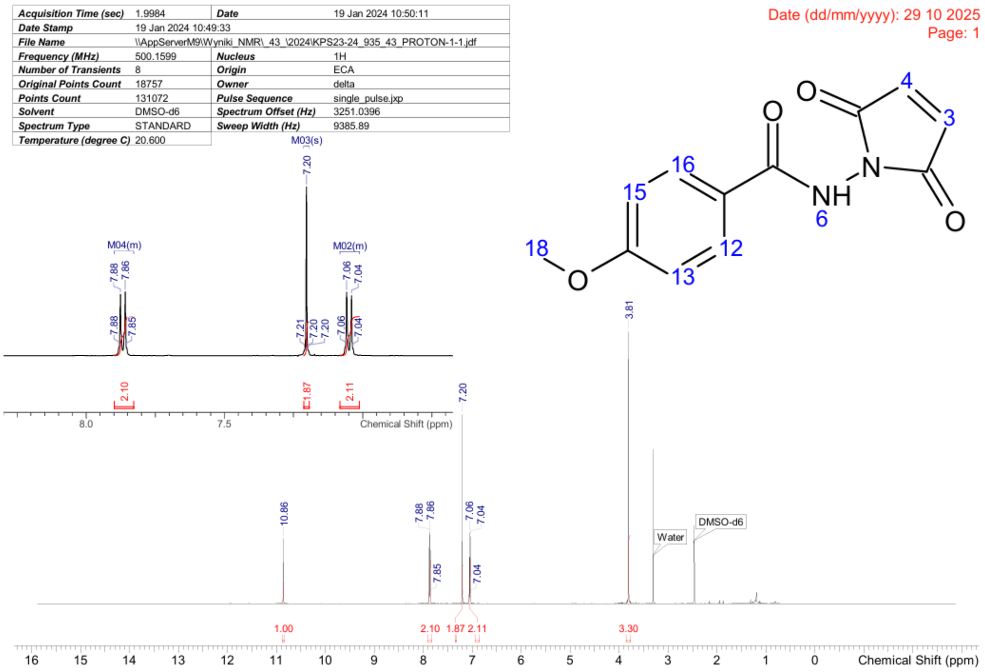


### 2.20.2. $^{13}$C NMR

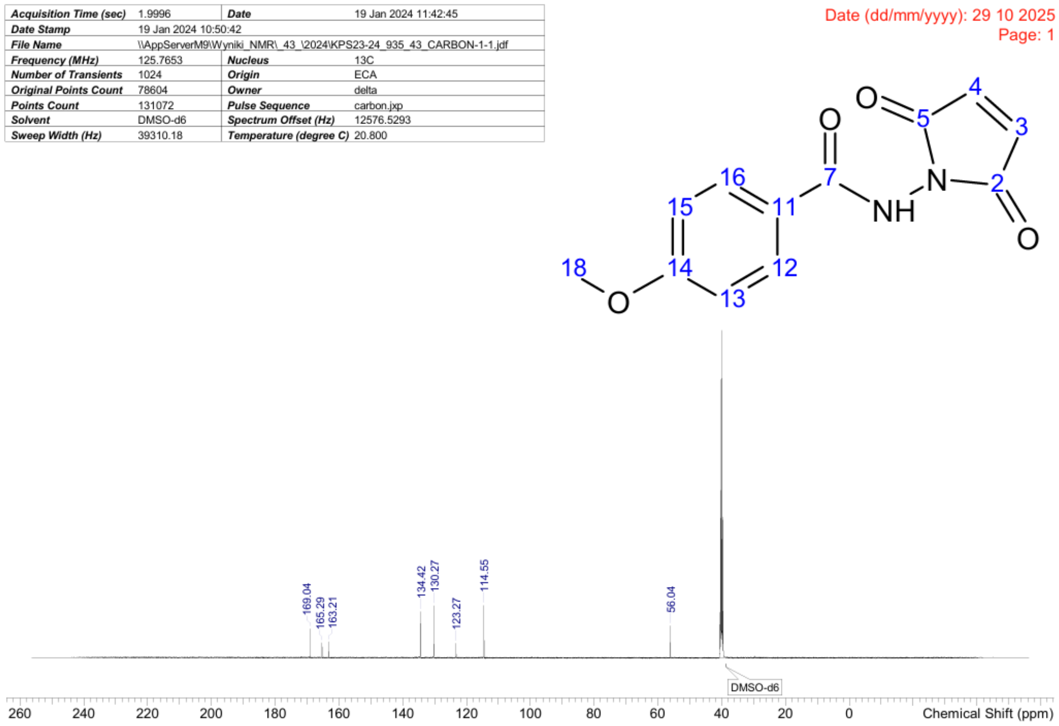

## **2.20.3.** LCMS

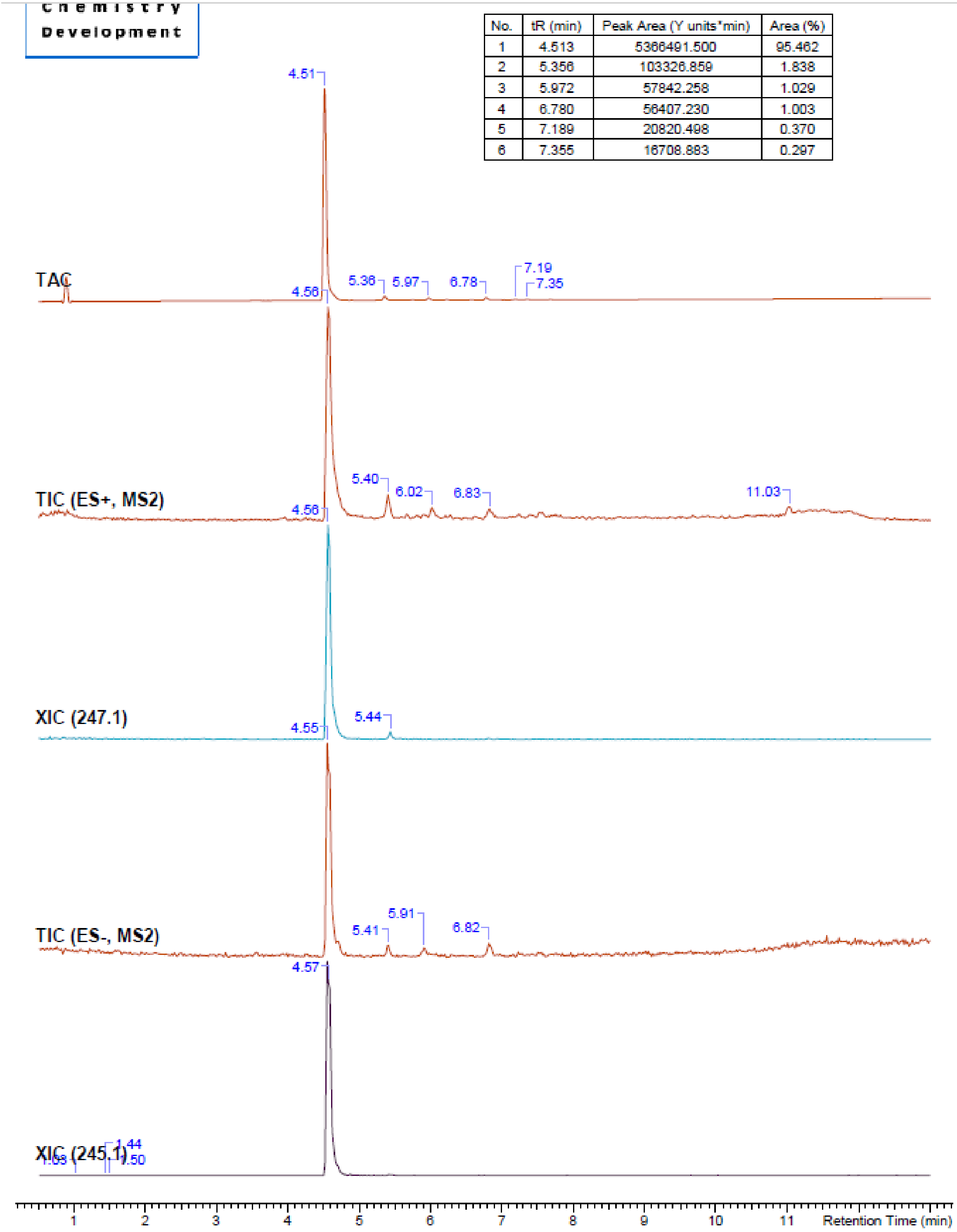


| No. | tR (min) | Peak Area (Y units*min) | Area (%) |
|---|---|---|---|
| 1 | 4.513 | 5366491.500 | 95.462 |
| 2 | 5.356 | 103326.859 | 1.838 |
| 3 | 5.972 | 57842.258 | 1.029 |
| 4 | 6.780 | 56407.230 | 1.003 |
| 5 | 7.189 | 20820.498 | 0.370 |
| 6 | 7.355 | 16708.883 | 0.297 |

| tR (min) | Mass (Ao) | [M+H]+ | [M-H]- | Area (%) |
|---|---|---|---|---|
| 4.513 | 246.064 | 247.071 | 245.057 | TIC (ES-, MS2) = 86.41<br>XIC = 88.24<br>TIC (ES+, MS2) = 82.61<br>XIC = 65.51<br>TAC = 95.46 |

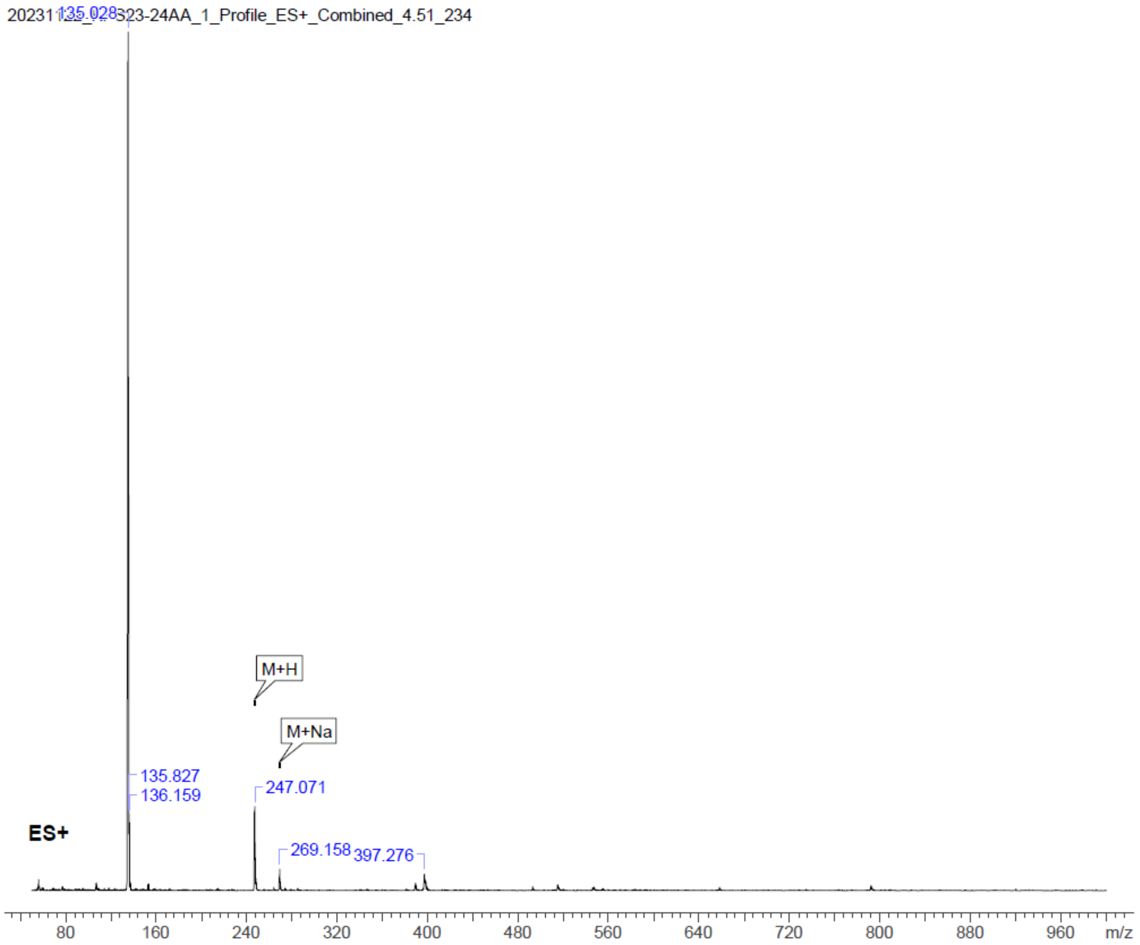

20231135.028323-24AA_1_Profile_ES+_Combined_4.51_234
ES+
135.827
136.159
M+H
247.071
M+Na
269.158
397.276
80
160
240
320
400
480
560
640
720
800
880
960
m/z

## 2.21. N-(2,5-dioxo-2,5-dihydro-1H-pyrrol-1-yl)thiophene-2-carboxamide 3h

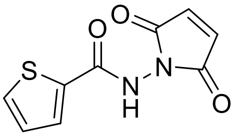

### 2.21.1. $^{1}$H NMR

05.12.2025 02:07:09

| Acquisition Time (sec) | 1.9984 | Date | 04 Dec 2025 10:21:44 | | | Date Stamp | 04 Dec 2025 10:21:06 |
|---|---|---|---|---|---|---|---|
| File Name | C:\Users\Admin\OneDrive - Uniwersytet Jagielloński\nmr\2025\Tiof-mal_935_03_PROTON-1-1.jdf | | | | | Frequency (MHz) | 500.16 |
| Nucleus | 1H | Number of Transients | 8 | Origin | ECA | Original Points Count | 18757 |
| Owner | delta | Points Count | 131072 | Pulse Sequence | single_pulse.jxp | Solvent | DMSO-d6 |
| Spectrum Offset (Hz) | 3251.0396 | Spectrum Type | STANDARD | Sweep Width (Hz) | 9385.89 | Temperature (degree C) | 20.800 |

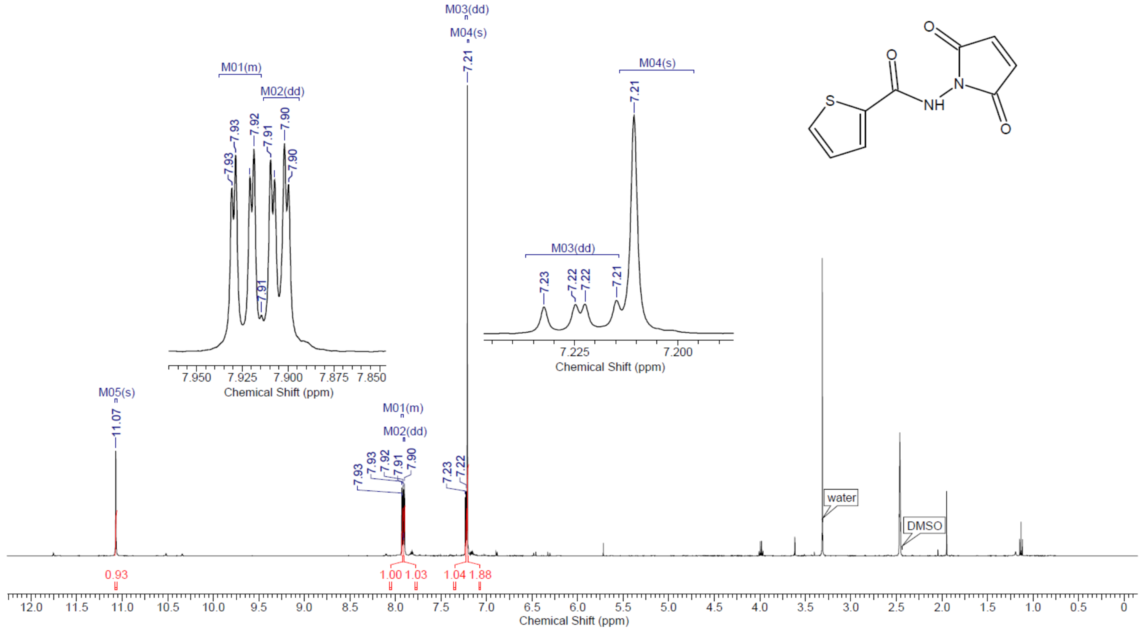

$^{13}$C NMR

06.12.2025 12:54:56

| Acquisition Time (sec) | 1.9996 | Date | 04 Dec 2025 11:14:18 | | | Date Stamp | 04 Dec 2025 10:22:15 |
|---|---|---|---|---|---|---|---|
| File Name | C:\Users\Admin\OneDrive - Uniwersytet Jagielloński\nmr\2025\Tiof-mal_935_03_CARBON-2-1.jdf | | | | | Frequency (MHz) | 125.77 |
| Nucleus | 13C | Number of Transients | 1024 | Origin | ECA | Original Points Count | 78604 |
| Owner | delta | Points Count | 131072 | Pulse Sequence | carbon.jxp | Solvent | DMSO-d6 |
| Spectrum Offset (Hz) | 12576.5293 | Sweep Width (Hz) | 39310.18 | Temperature (degree C) | 20.800 | | |

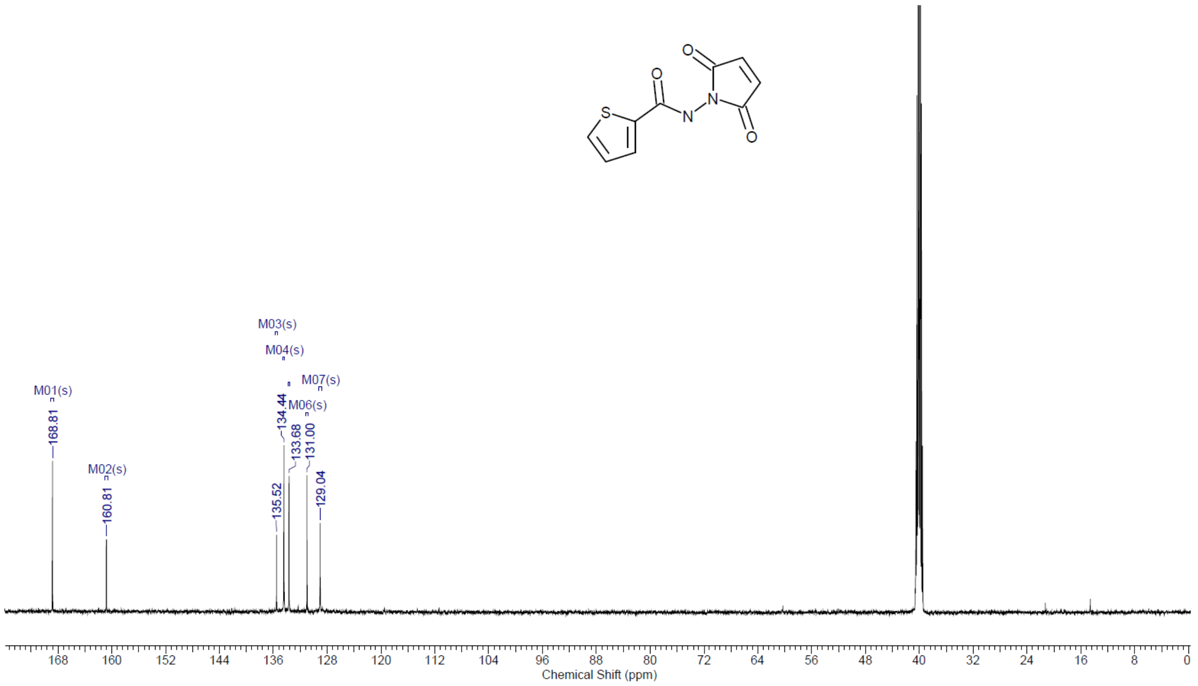

### 2.21.2. UPLC-MS

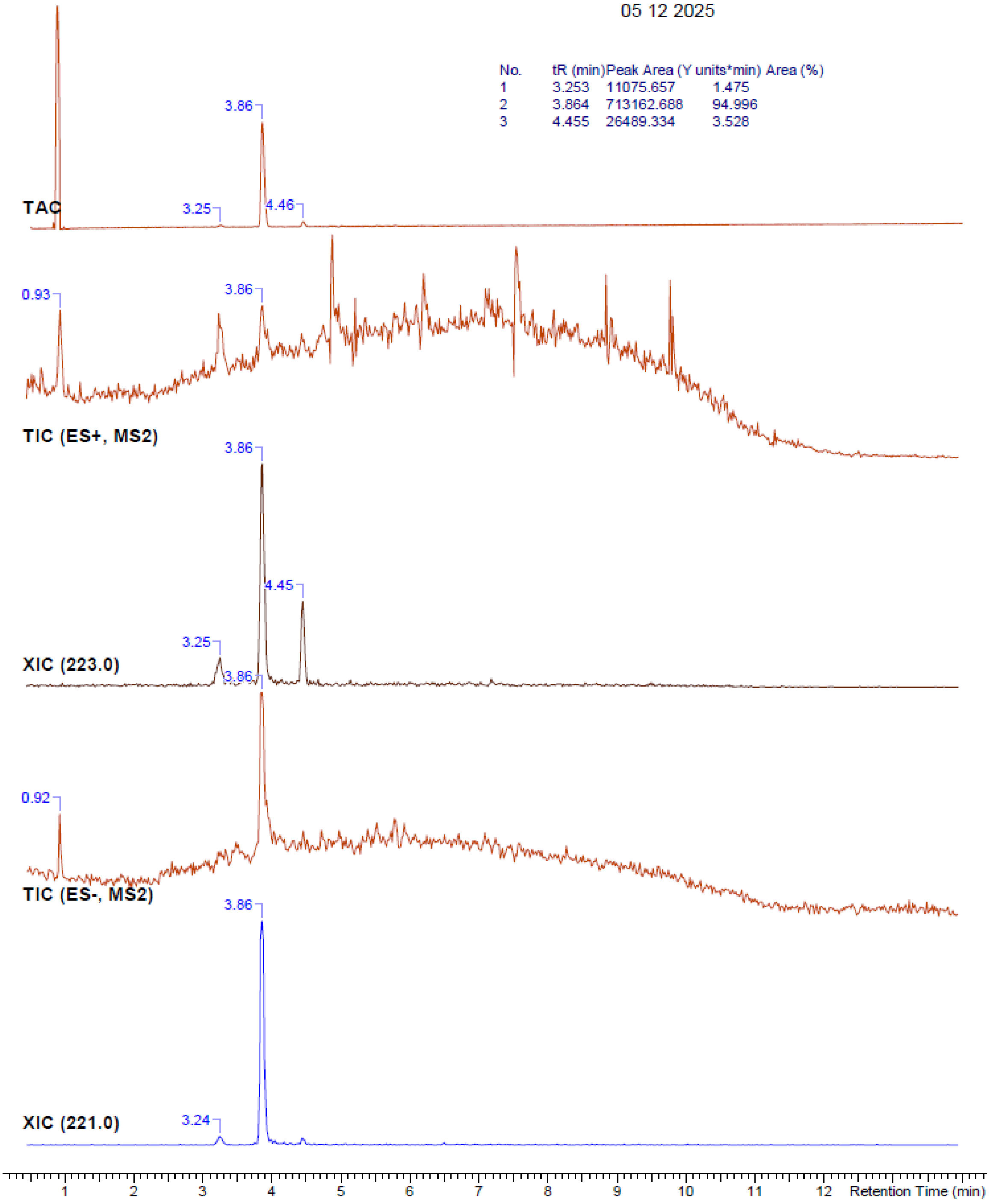


| Comp | tR(min) | Mass(Ao) | [M+H]+ | [M-H]- | M+· | M-· | MF | Structure | Area (%) |
|---|---|---|---|---|---|---|---|---|---|
| 1 | 3.860 | 222.010 | 223.017 | 221.003 | 222.009 | 222.010 | $C_9H_6N_2C$ | 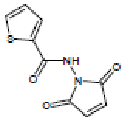 | TIC (ES-, MS2) = 87.18<br>Single XIC = 95.39<br>TIC (ES+, MS2) = 56.37<br>Single XIC = 66.15<br>TAC = 95.00 |

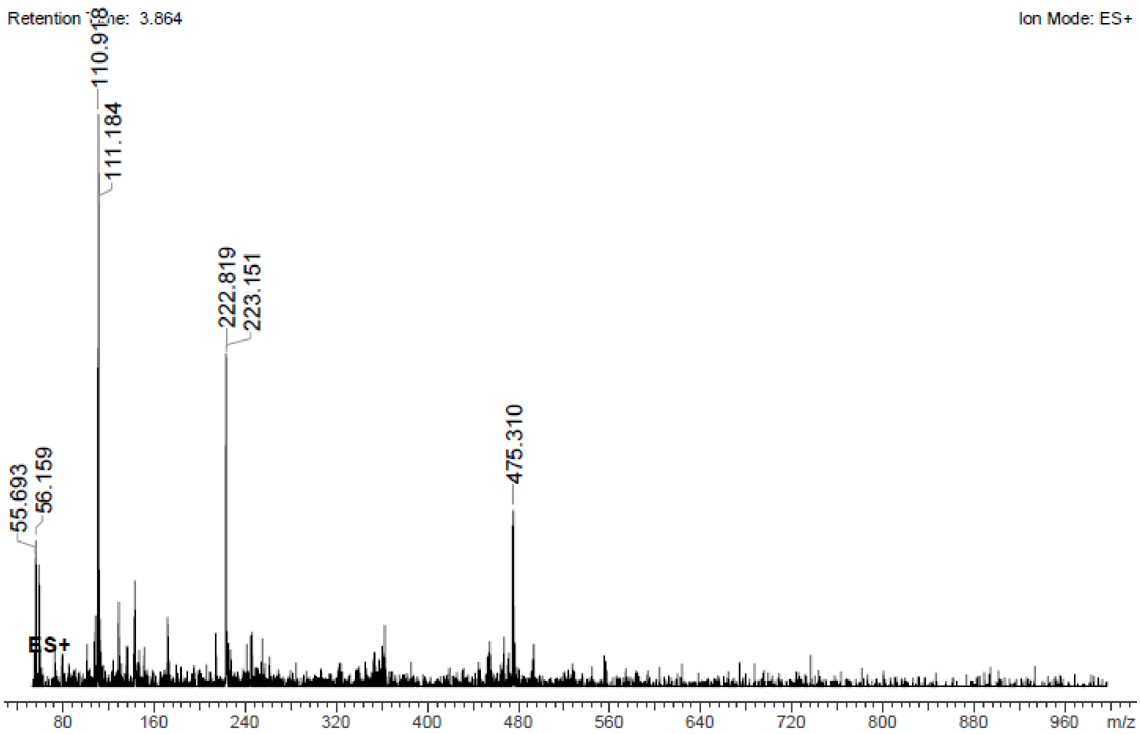
Retention Time: 3.864
Ion Mode: ES+
110.918
111.184
222.819
223.151
475.310
55.693
56.159
ES+
80
160
240
320
400
480
560
640
720
800
880
960
m/z

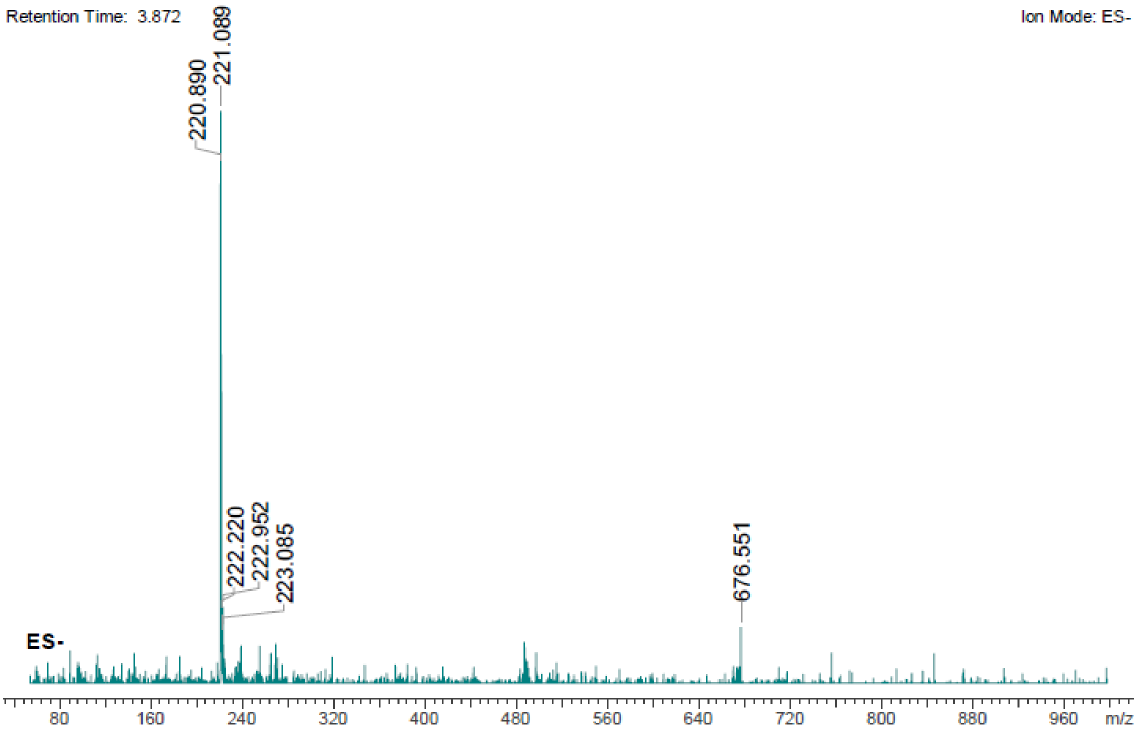
Retention Time: 3.872
Ion Mode: ES-
220.890
221.089
222.220
222.952
223.085
676.551
ES-
80
160
240
320
400
480
560
640
720
800
880
960
m/z

## 2.22. N'-(3-oxo-3,4,4a,5,5a,6-hexahydro-1H-4,6-ethenocyclopropa[f]isobenzofuran-1-ylidene)-4-(trifluoromethyl)benzohydrazide (4)

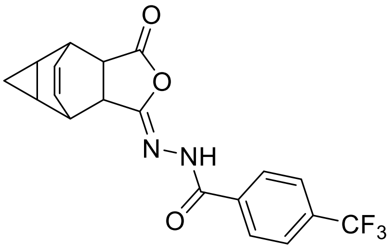


### 2.22.1. $^{1}$H NMR

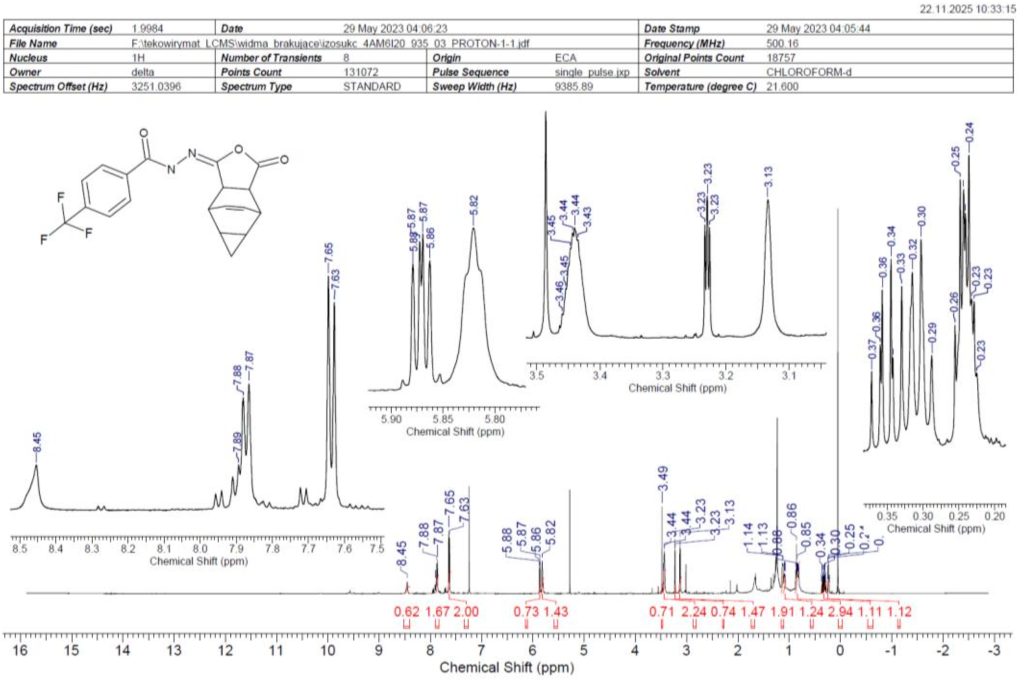


### 2.22.2. $^{13}$C NMR

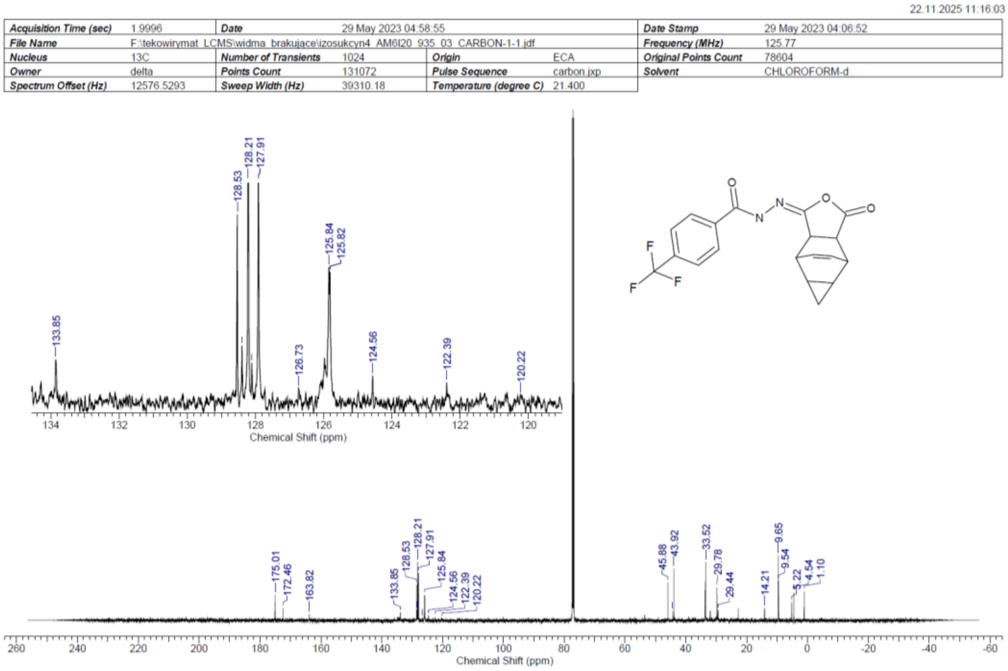

### **2.22.3.** LCMS

21 11 2025

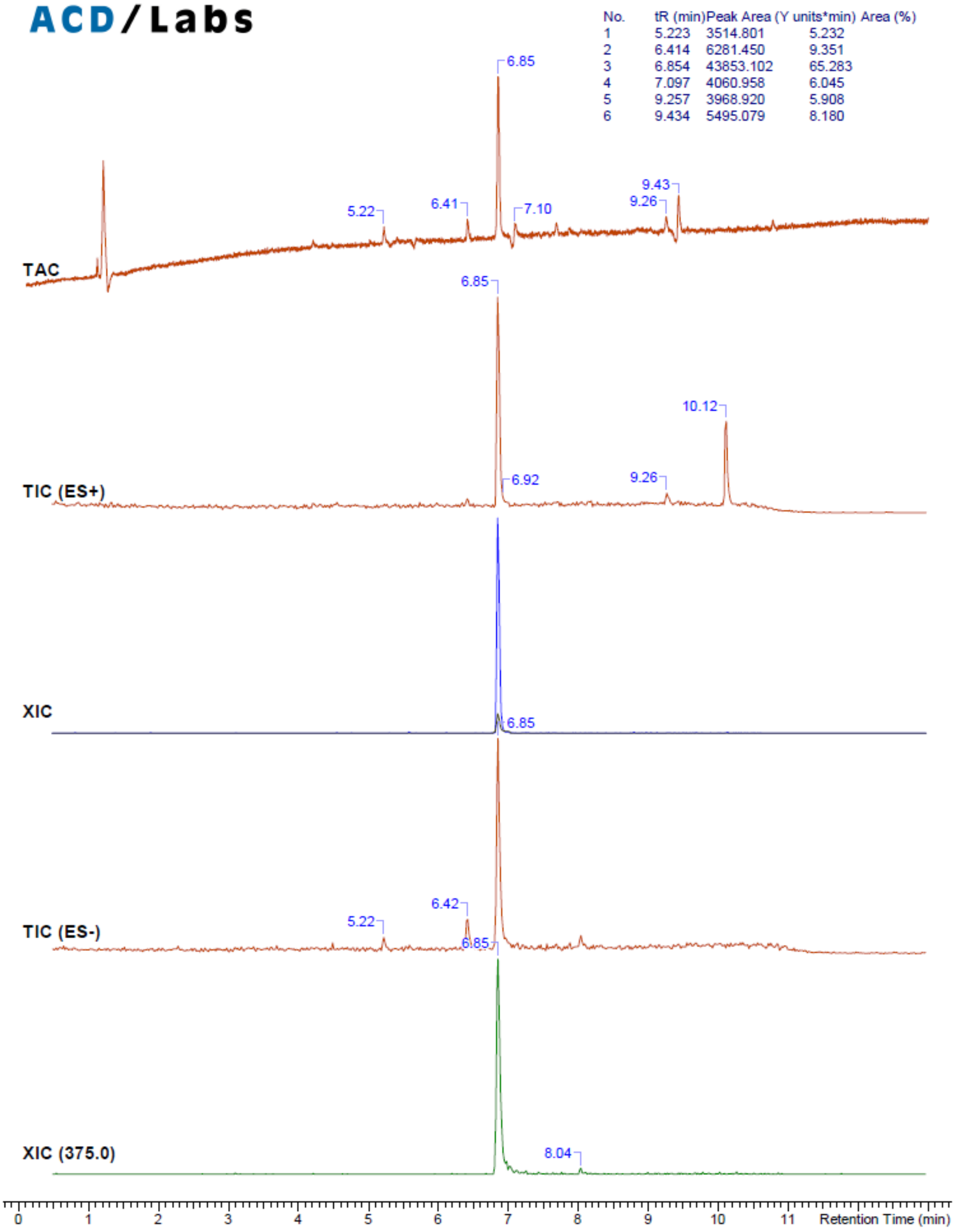


| Compo | tR(min) | Mass(Ao) | [M+H]+ | [M-H]- | M+· | M-· | MF | Structure | Area (%) |
|---|---|---|---|---|---|---|---|---|---|
| 1 | 6.846 | 376.103 | 377.111 | 375.096 | 376.103 | 376.104 | $C_{19}H_{15}F_3N_2O_3$ | 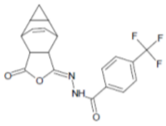 | TIC (ES-) = 82.16<br>Single XIC = 98.69<br>TIC (ES+) = 41.10<br>Single XIC = 99.66<br>TAC = 65.28 |

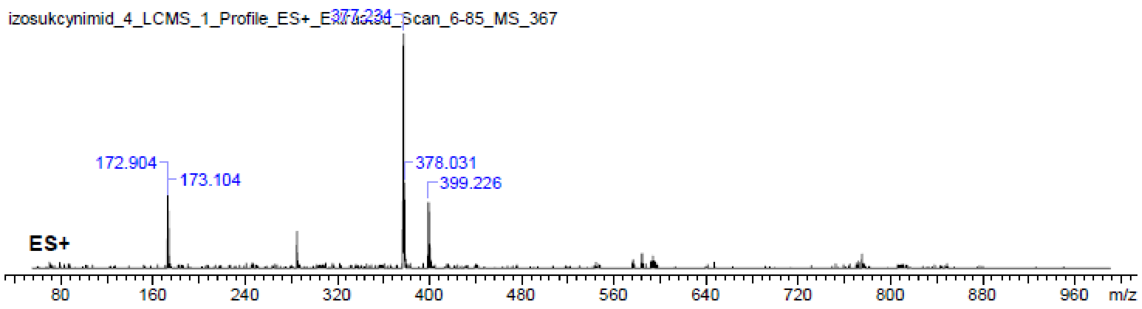
izosukcynimid_4_LCMS_1_Profile_ES+_Extracted_Scan_6-85_MS_367
377.234
172.904
173.104
378.031
399.226
ES+
80
160
240
320
400
480
560
640
720
800
880
960
m/z

## 2.23. *N*-(1,3-Dioxo-3,3a,4,4a,5,5a,6,6a-octahydro-4,6-ethenocyclopropa[f]isoindol-2(*1H*)-yl)-4-(trifluoromethyl)benzamide (Tecovirimat, 5)

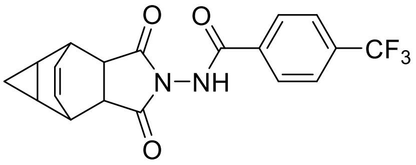

### 2.23.1. $^{1}$H NMR

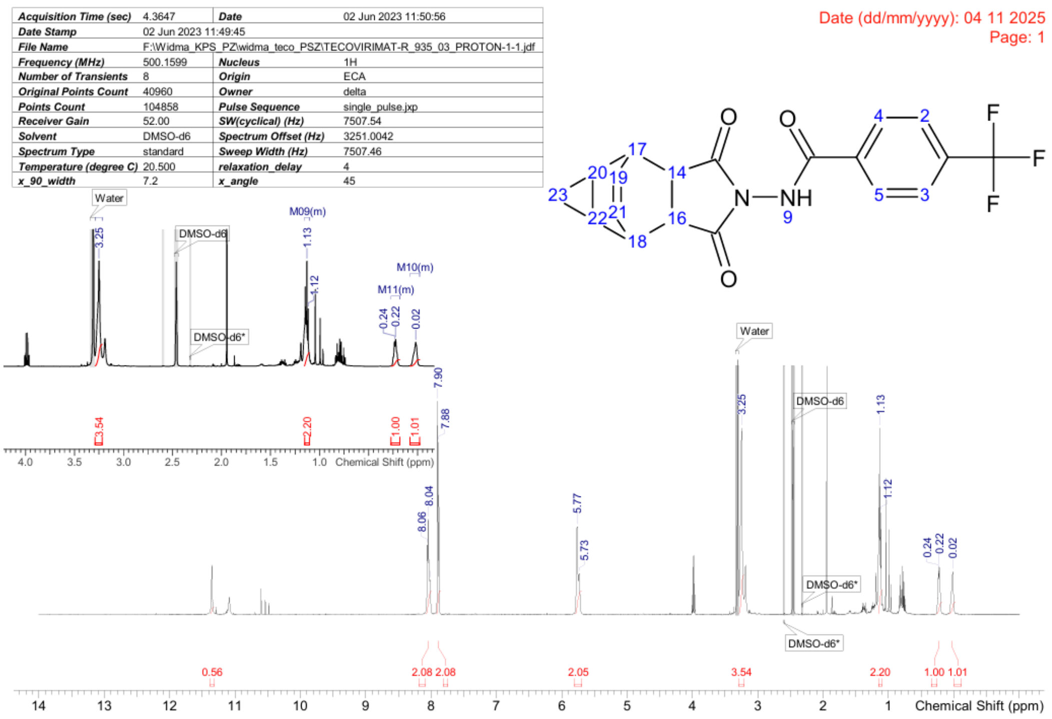


### 2.23.2. $^{13}$C NMR

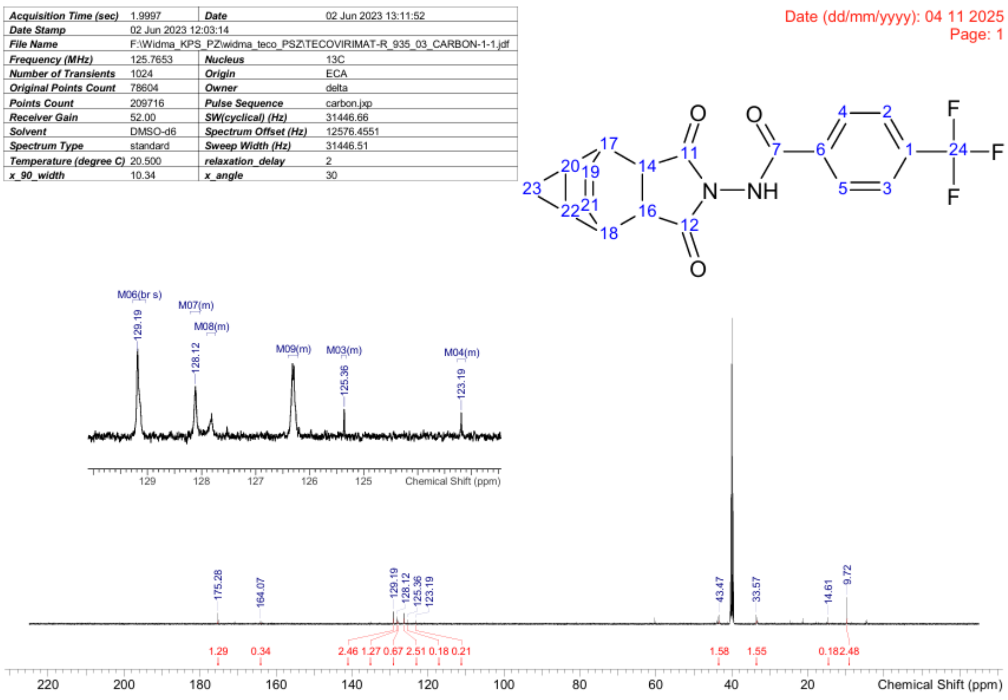

## 2.23.3. $^{19}$F NMR

| Acquisition Time (sec) | 2.9861 | Date | 02 Jun 2023 11:53:41 |
| --- | --- | --- | --- |
| Date Stamp | 02 Jun 2023 11:52:39 | | |
| File Name | F:\Widma_KPS_PZ\widma_teco_PSZ\TECOVIRIMAT-R_935_03_Fluorine-1-1.jdf | | |
| Frequency (MHz) | 470.6205 | Nucleus | 19F |
| Number of Transients | 8 | Origin | ECA |
| Original Points Count | 352967 | Owner | delta |
| Points Count | 838862 | Pulse Sequence | single_pulse.jxp |
| Receiver Gain | 62.00 | SW(cyclical) (Hz) | 94562.79 |
| Solvent | DMSO-d6 | Spectrum Offset (Hz) | -47062.1016 |
| Spectrum Type | standard | Sweep Width (Hz) | 94562.68 |
| Temperature (degree C) | 20.500 | relaxation_delay | 4 |
| x_90_width | 7.79 | x_angle | 45 |

Date (dd/mm/yyyy): 03 11 2025
Page: 1

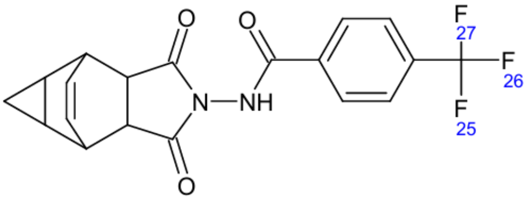


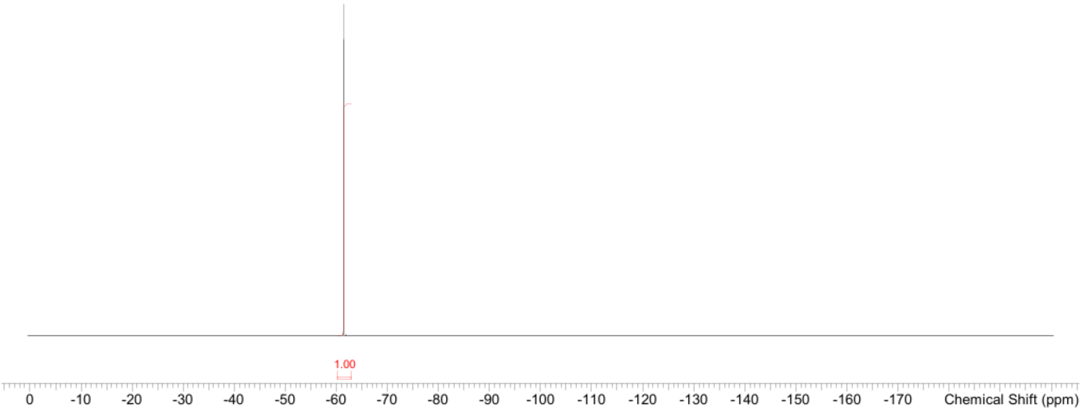

**2.23.4.** LCMS

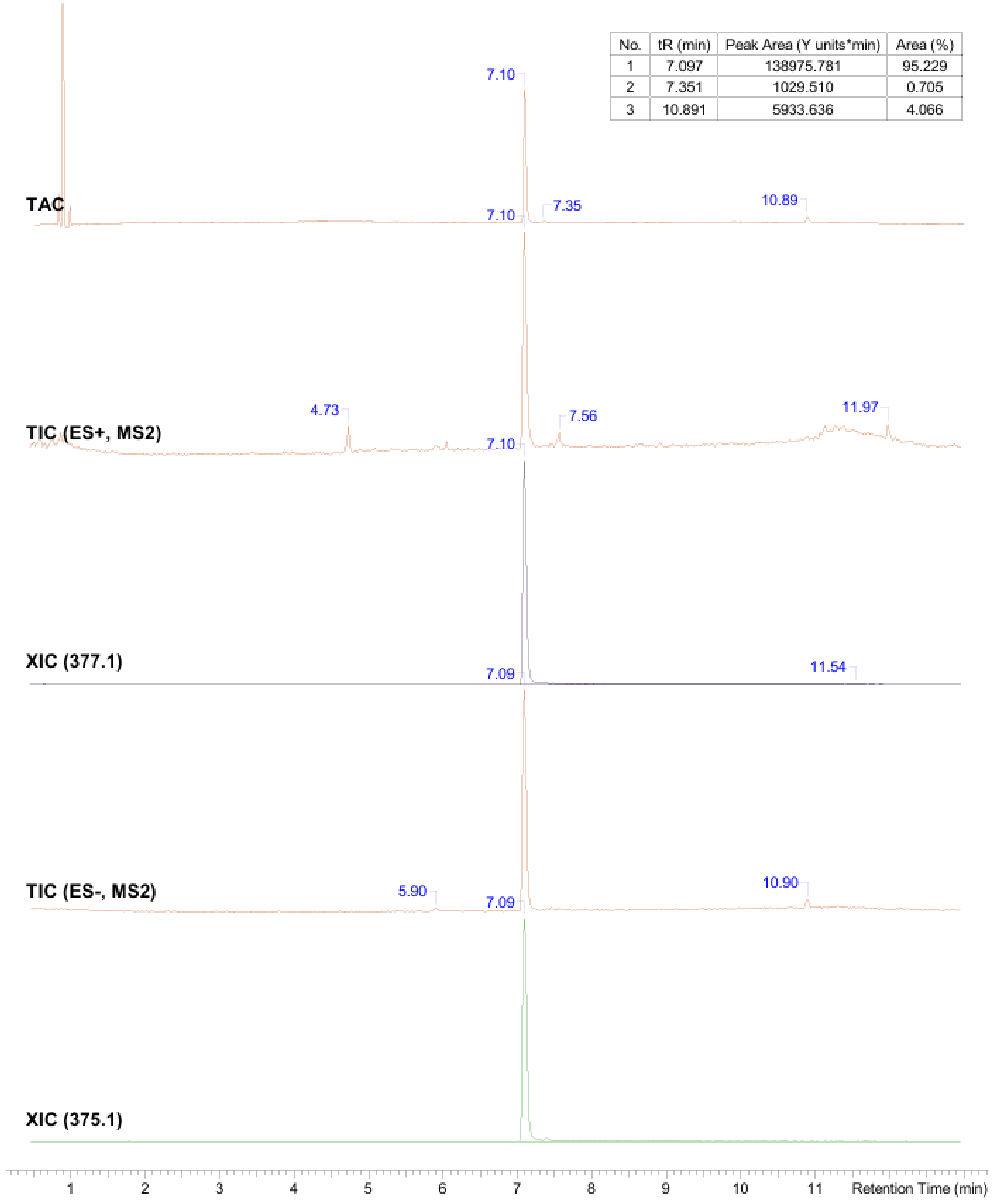


| No. | tR (min) | Peak Area (Y units*min) | Area (%) |
|---|---|---|---|
| 1 | 7.097 | 138975.781 | 95.229 |
| 2 | 7.351 | 1029.510 | 0.705 |
| 3 | 10.891 | 5933.636 | 4.066 |

| Compo | tR(min) | Mass(Ao) | [M+H]+ | [M-H]- | M+· | M-· | MF | Structure | Area (%) |
|---|---|---|---|---|---|---|---|---|---|
| 1 | 7.097 | 376.103 | 377.111 | 375.096 | 376.103 | 376.104 | $C_{19}H_{15}F_3N_2O_3$ | 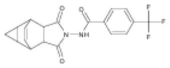 | TIC (ES-, MS2) = 95.69<br>Single XIC = 100.00<br>TIC (ES+, MS2) = 87.09<br>Single XIC = 99.86<br>TAC = 95.23 |

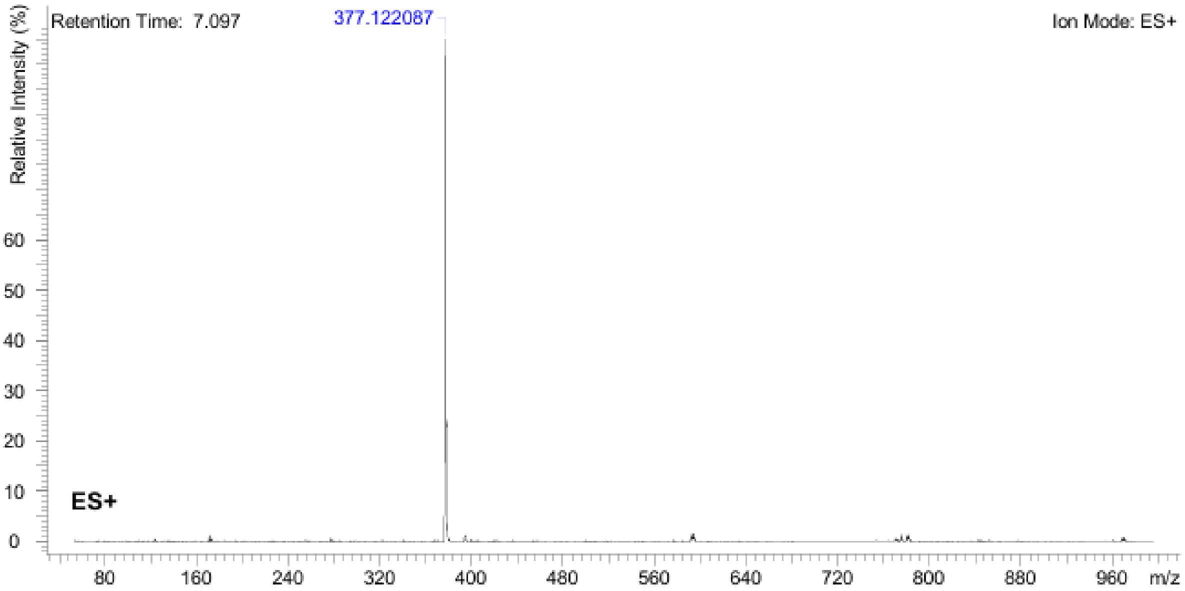
Retention Time: 7.097
377.122087
Ion Mode: ES+
Relative Intensity (%)
60
50
40
30
20
10
0
ES+
80
160
240
320
400
480
560
640
720
800
880
960
m/z

## 3. Computational results

The computational results for isomaleimides **2a-f, 2h** and isosuccinimide **4** listed below include cartesian coordinates and heat of formation values for the lowest-ΔHf conformations, charge distribution and graphical representations of Molecular Electrostatic Potential (MEP) as well as LUMO and HOMO orbitals with respective energies given in eV.

### 3.8. *N'*-(5-Oxofuran-2(5H)-ylidene)-4-(trifluoromethyl)benzohydrazide (2a)

**Table S1:** Heat of formation, atomic coordinates and Net charges for compound **2a**

| $\Delta H_f$= 195.81666 kcal/mol | | | | | |
|---|---|---|---|---|---|
| Atom No. | Element | X | Y | Z | Charge |
| 1 | C | -2.794885 | 3.172267 | -0.563112 | -0.103436 |
| 2 | C | -2.385314 | 3.923163 | -1.663580 | -0.120605 |
| 3 | C | -3.129861 | 3.874683 | -2.840226 | -0.094041 |
| 4 | C | -4.268431 | 3.070335 | -2.901056 | -0.117186 |
| 5 | C | -4.686355 | 2.332116 | -1.794332 | -0.096921 |
| 6 | C | -3.943694 | 2.384254 | -0.615951 | -0.104778 |
| 7 | C | -2.036781 | 3.224658 | 0.712077 | 0.571875 |
| 8 | N | -0.709562 | 2.821553 | 0.624449 | -0.395417 |
| 9 | O | -2.517299 | 3.567497 | 1.774465 | -0.560471 |
| 10 | F | -5.812211 | 1.940966 | -4.348482 | -0.207149 |
| 11 | N | 0.204203 | 2.904457 | 1.628264 | -0.220851 |
| 12 | C | -0.078893 | 2.709416 | 2.874185 | 0.296128 |
| 13 | C | 0.667532 | 2.646329 | 5.027432 | 0.615774 |
| 14 | O | 1.054296 | 2.882417 | 3.682339 | -0.392251 |
| 15 | C | -1.211862 | 2.333277 | 3.754364 | -0.073356 |
| 16 | C | -0.769055 | 2.295861 | 5.022102 | -0.248698 |
| 17 | O | 1.505079 | 2.757675 | 5.869056 | -0.435558 |
| 18 | C | -5.062016 | 3.029493 | -4.174216 | 0.552244 |
| 19 | F | -4.332102 | 3.084940 | -5.289749 | -0.209010 |
| 20 | F | -5.923572 | 4.041821 | -4.297248 | -0.208564 |
| 21 | H | -1.493643 | 4.554952 | -1.610519 | 0.186590 |
| 22 | H | -2.808946 | 4.473646 | -3.698273 | 0.187278 |
| 23 | H | -5.589118 | 1.714647 | -1.824244 | 0.186669 |
| 24 | H | -4.264936 | 1.811428 | 0.259162 | 0.185279 |
| 25 | H | -0.244999 | 2.640945 | -0.280223 | 0.374824 |
| 26 | H | -2.203164 | 2.119376 | 3.373690 | 0.213373 |
| 27 | H | -1.297606 | 2.056938 | 5.933788 | 0.218259 |

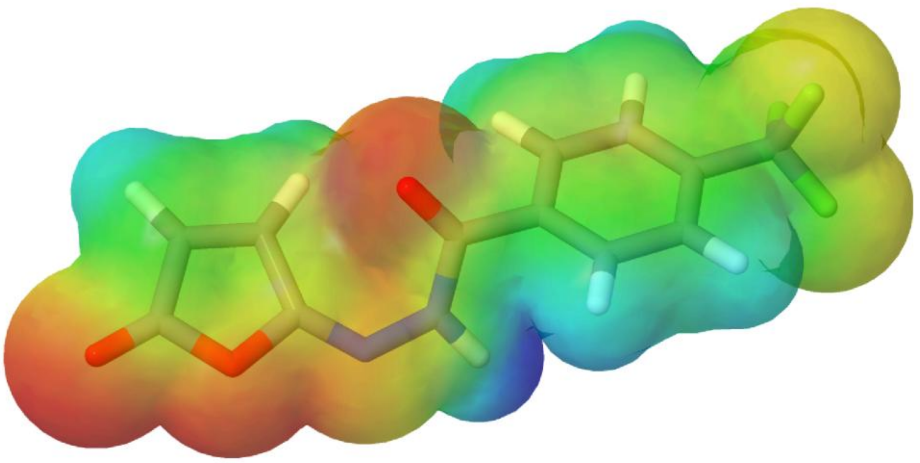

**Figure S1:** Molecular Electrostatic Potential surface for compound **2a**

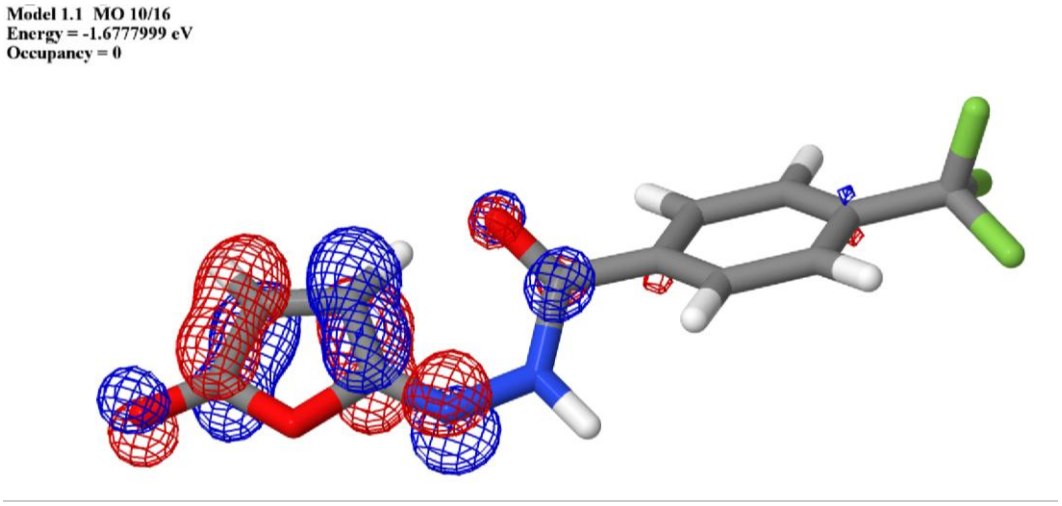


**Figure S2:** Graphical representation of LUMO orbital for compound **2a**

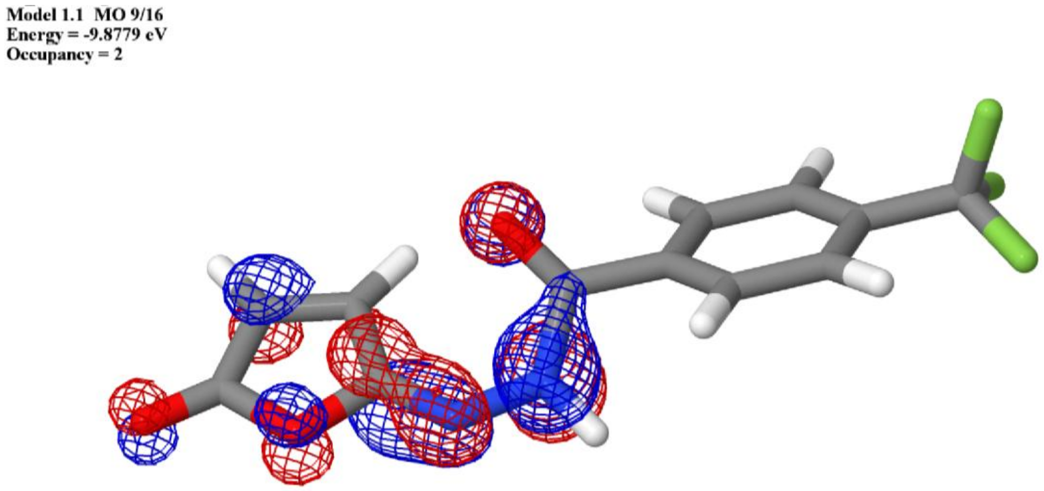


**Figure S3:** Graphical representation of HOMO orbital for compound **2a**

## *Tert*-butyl 2-(5-oxofuran-2(*5H*)-ylidene)hydrazine-1-carboxylate (2b)

**Table S2:** Heat of formation, atomic coordinates and Net charges for compound **2b**

| ΔH$_f$= -132.16417 kcal/mol | | | | | |
|---|---|---|---|---|---|
| Atom No. | Element | X | Y | Z | Net Charge |
| 1 | O | 2.742612 | 0.122851 | -1.614114 | -0.485113 |
| 2 | C | 3.913625 | 0.899692 | -2.041306 | 0.395310 |
| 3 | C | 2.665697 | -1.181438 | -1.887277 | 0.741281 |
| 4 | N | 1.459548 | -1.739011 | -1.417344 | -0.419945 |
| 5 | O | 3.497242 | -1.883693 | -2.423133 | -0.592498 |
| 6 | N | 0.381691 | -0.984376 | -1.036904 | -0.227382 |
| 7 | C | 0.498070 | -0.052069 | -0.149209 | 0.303401 |
| 8 | C | -0.462010 | 1.598249 | 1.087817 | 0.613281 |
| 9 | O | -0.715893 | 0.585400 | 0.123560 | -0.381658 |
| 10 | C | 1.546830 | 0.566779 | 0.699936 | -0.083448 |
| 11 | C | 0.977631 | 1.540144 | 1.428633 | -0.243231 |
| 12 | O | -1.382348 | 2.270581 | 1.437021 | -0.433696 |
| 13 | C | 5.122899 | 0.396687 | -1.273339 | -0.518344 |
| 14 | C | 4.060890 | 0.784931 | -3.547710 | -0.518497 |
| 15 | C | 3.476158 | 2.296838 | -1.606859 | -0.493627 |
| 16 | H | 1.142057 | -2.617730 | -1.872952 | 0.373309 |
| 17 | H | 2.572363 | 0.220244 | 0.694918 | 0.208414 |
| 18 | H | 1.412323 | 2.202747 | 2.162898 | 0.216716 |
| 19 | H | 4.944221 | 0.380343 | -0.190661 | 0.165712 |
| 20 | H | 5.997334 | 1.037458 | -1.450392 | 0.173016 |
| 21 | H | 5.405294 | -0.622951 | -1.572627 | 0.177092 |
| 22 | H | 4.415489 | -0.208613 | -3.856346 | 0.177379 |
| 23 | H | 3.108500 | 0.966482 | -4.063539 | 0.170908 |
| 24 | H | 4.783824 | 1.517256 | -3.932097 | 0.173321 |
| 25 | H | 4.243458 | 3.038918 | -1.863863 | 0.174104 |
| 26 | H | 2.544826 | 2.602774 | -2.102367 | 0.170904 |
| 27 | H | 3.307402 | 2.359432 | -0.524909 | 0.163291 |

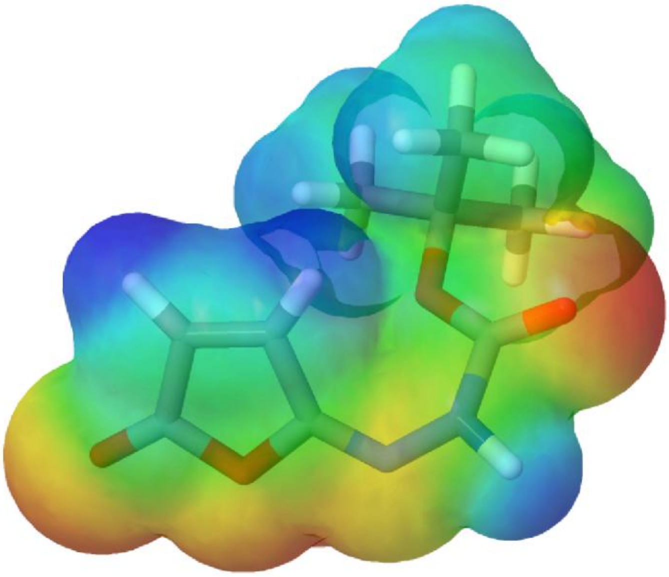

**Figure S4:** Molecular electrostatic potential surface for compound **2b**

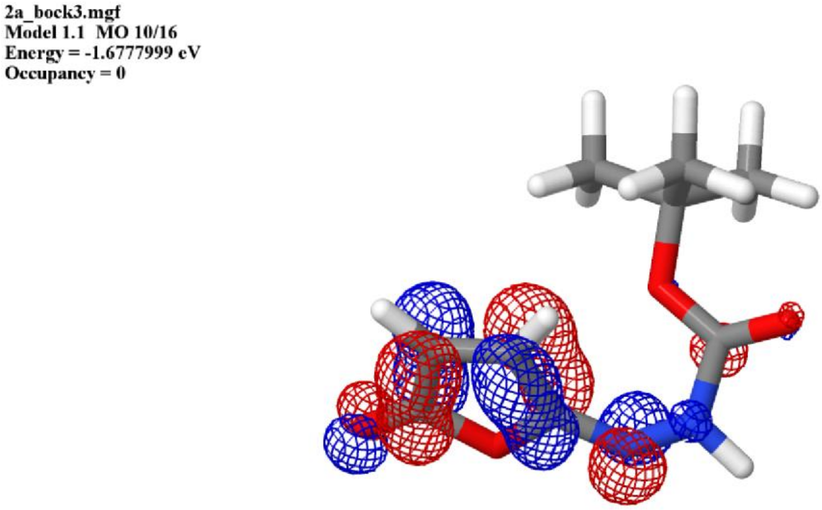


**Figure S5:** Graphical representation of LUMO orbital for compound **2b**

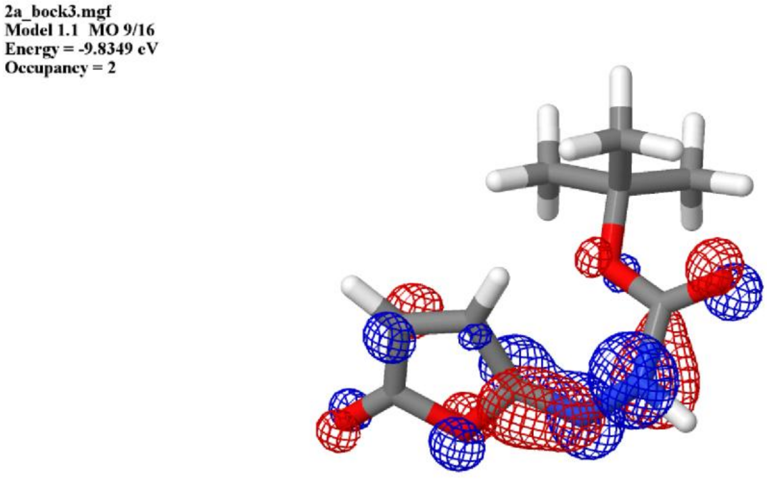


**Figure S6:** Graphical representation of HOMO orbital for compound **2b**

## *N'*-(5-Oxofuran-2(*5H*)-ylidene)benzohydrazide (2c)

**Table S3:** Heat of formation, atomic coordinates and Net charges for compound **2c**

| ΔHf= -30.79529 kcal/mol | | | | | |
|---|---|---|---|---|---|
| Atom No. | Element | X | Y | Z | Charge |
| 1 | C | -3.310300169 | 1.988749772 | -1.270934116 | -0.158576 |
| 2 | C | -2.567828802 | 2.488296597 | -2.354330800 | -0.072560 |
| 3 | C | -3.020474927 | 3.599728154 | -3.056250856 | -0.191219 |
| 4 | C | -4.201605065 | 4.235984237 | -2.671855214 | -0.092312 |
| 5 | C | -4.934714881 | 3.746419904 | -1.594370550 | -0.186713 |
| 6 | C | -4.498237304 | 2.618760939 | -0.902543246 | -0.073179 |
| 7 | C | -2.782374657 | 0.780710958 | -0.612377349 | 0.639709 |
| 8 | N | -3.391356606 | 0.067762765 | 0.434710891 | -0.421094 |
| 9 | O | -1.764717768 | 0.225908866 | -1.007156648 | -0.594337 |
| 10 | N | -4.373273138 | 0.589883446 | 1.217055032 | -0.184113 |
| 11 | C | -4.990500624 | -0.211693496 | 2.031364634 | 0.237159 |
| 12 | C | -6.380721606 | -0.602974586 | 3.781806320 | 0.609023 |
| 13 | O | -5.904585270 | 0.401344514 | 2.888991203 | -0.364700 |
| 14 | C | -4.959477727 | -1.661356140 | 2.334554122 | -0.102946 |
| 15 | C | -5.779997279 | -1.892241949 | 3.372389592 | -0.229614 |
| 16 | O | -7.130735286 | -0.261193296 | 4.643455042 | -0.440169 |
| 17 | H | -1.633149164 | 2.001177273 | -2.651139682 | 0.177157 |
| 18 | H | -2.458385497 | 3.976043957 | -3.911032826 | 0.168189 |
| 19 | H | -4.551456731 | 5.115957483 | -3.214800708 | 0.161602 |
| 20 | H | -5.857240426 | 4.239671336 | -1.288990585 | 0.169723 |
| 21 | H | -5.104051152 | 2.238968161 | -0.075561302 | 0.186044 |
| 22 | H | -2.913079134 | -0.795347692 | 0.740391584 | 0.341023 |
| 23 | H | -4.376272106 | -2.362438041 | 1.753920795 | 0.206228 |
| 24 | H | -6.024913612 | -2.819185241 | 3.872344935 | 0.215673 |

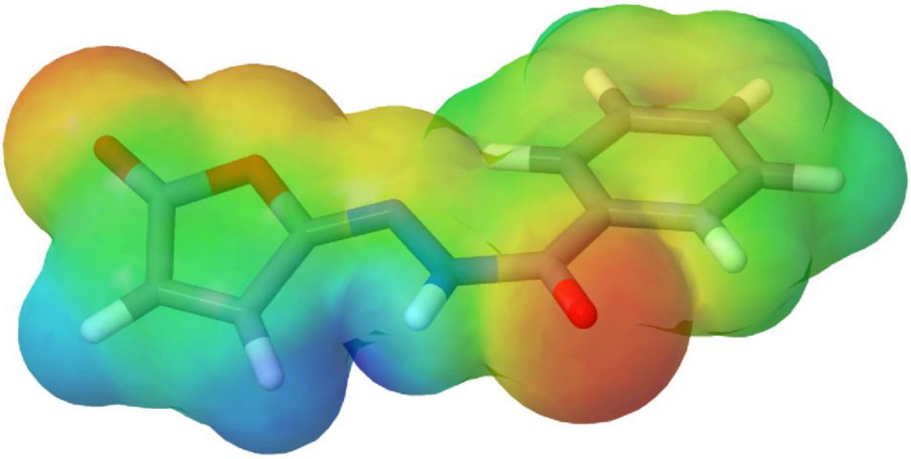

**Figure S7:** Molecular electrostatic potential surface for compound **2c**

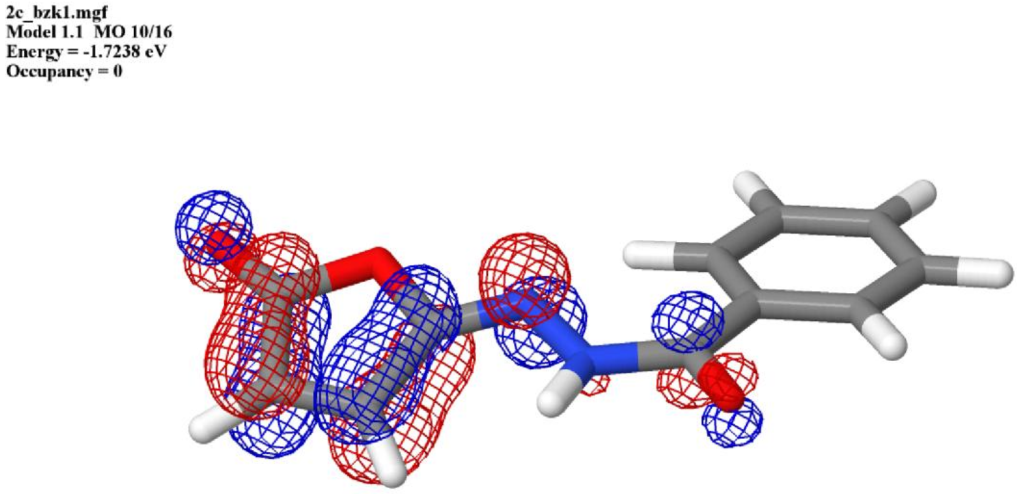


**Figure S8:** Graphical representation of LUMO orbital for compound **2c**

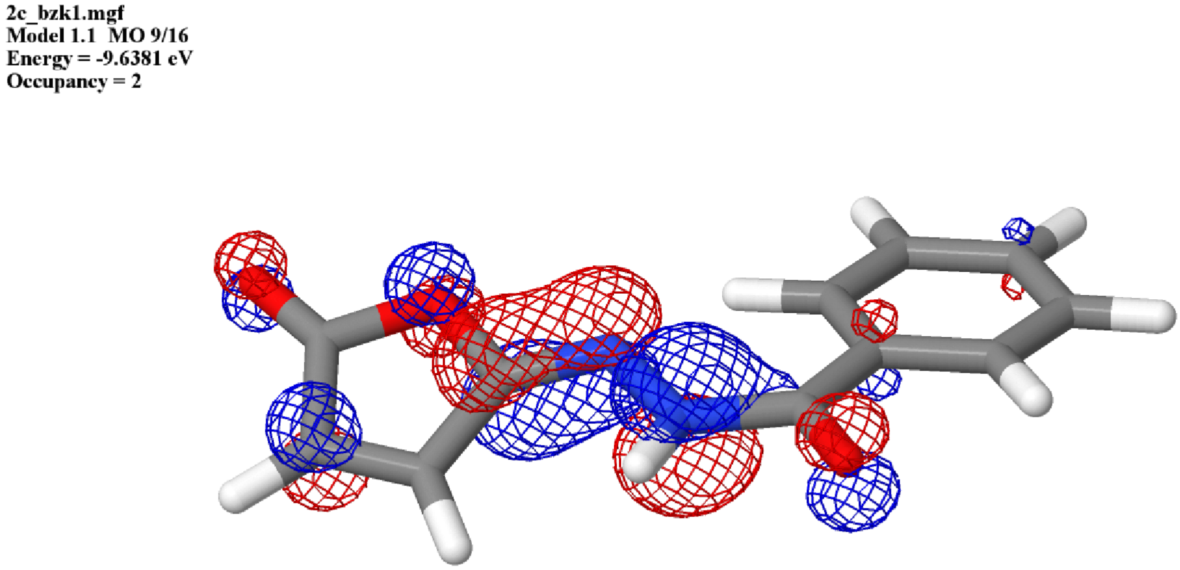


**Figure S9:** Graphical representation of HOMO orbital for compound **2c**

## 4-(Dimethylamino)-N'-(5-oxofuran-2(*5H*)-ylidene)benzohydrazide (2d)

**Table S4:** Heat of formation, atomic coordinates and net charges for compound **2d**

| ΔHf = -41.95193 kcal/mol | | | | | |
|---|---|---|---|---|---|
| Atom No. | Element | X | Y | Z | Charge |
| 1 | C | -2.908996771 | 2.966759508 | 0.628357206 | -0.296697 |
| 2 | C | -2.394197058 | 4.163955151 | 0.111242429 | 0.012251 |
| 3 | C | -2.608737519 | 5.363084042 | 0.762202807 | -0.341241 |
| 4 | C | -3.349917066 | 5.402793804 | 1.971892572 | 0.291036 |
| 5 | C | -3.819211445 | 4.175950726 | 2.510904344 | -0.335304 |
| 6 | C | -3.608441175 | 2.986682198 | 1.840961065 | -0.008013 |
| 7 | C | -2.652362812 | 1.736682358 | -0.122105243 | 0.618385 |
| 8 | N | -3.642282456 | 0.743212255 | -0.042464058 | -0.410460 |
| 9 | O | -1.658303782 | 1.529659317 | -0.794627719 | -0.592676 |
| 10 | N | -3.700503321 | 6.606351290 | 2.548074245 | -0.381793 |
| 11 | N | -3.662581777 | -0.397012431 | -0.774762073 | -0.211612 |
| 12 | C | -2.608858731 | -1.062100254 | -1.114108275 | 0.274847 |
| 13 | C | -1.764652380 | -2.871100472 | -2.215258301 | 0.620152 |
| 14 | O | -2.958622887 | -2.191804290 | -1.878550869 | -0.395961 |
| 15 | C | -1.136481527 | -1.064006790 | -0.956386209 | -0.058659 |
| 16 | C | -0.640434264 | -2.130948398 | -1.606665924 | -0.267784 |
| 17 | O | -1.859249434 | -3.858746650 | -2.880194173 | -0.455458 |
| 18 | C | -2.988976355 | 7.826286773 | 2.141940307 | -0.260837 |
| 19 | C | -4.171080868 | 6.622711848 | 3.939630309 | -0.261345 |
| 20 | H | -1.814571399 | 4.143871096 | -0.819428358 | 0.173884 |
| 21 | H | -2.200226132 | 6.277505785 | 0.337702396 | 0.178438 |
| 22 | H | -4.351213008 | 4.161902962 | 3.459801408 | 0.177025 |
| 23 | H | -3.981930959 | 2.054854547 | 2.278862879 | 0.168618 |
| 24 | H | -4.584615134 | 0.953461171 | 0.323374168 | 0.367555 |
| 25 | H | -0.608566079 | -0.311522123 | -0.382055216 | 0.214778 |
| 26 | H | 0.381070962 | -2.463943459 | -1.710730789 | 0.215144 |
| 27 | H | -1.900854431 | 7.766338430 | 2.321117657 | 0.153258 |
| 28 | H | -3.373271633 | 8.703610615 | 2.692954647 | 0.165791 |
| 29 | H | -3.161845031 | 8.026187101 | 1.067623794 | 0.164386 |
| 30 | H | -3.409427059 | 6.257756488 | 4.650376483 | 0.152250 |
| 31 | H | -4.453869252 | 7.647467198 | 4.242136539 | 0.167014 |
| 32 | H | -5.078952254 | 6.000959857 | 4.045227047 | 0.163024 |

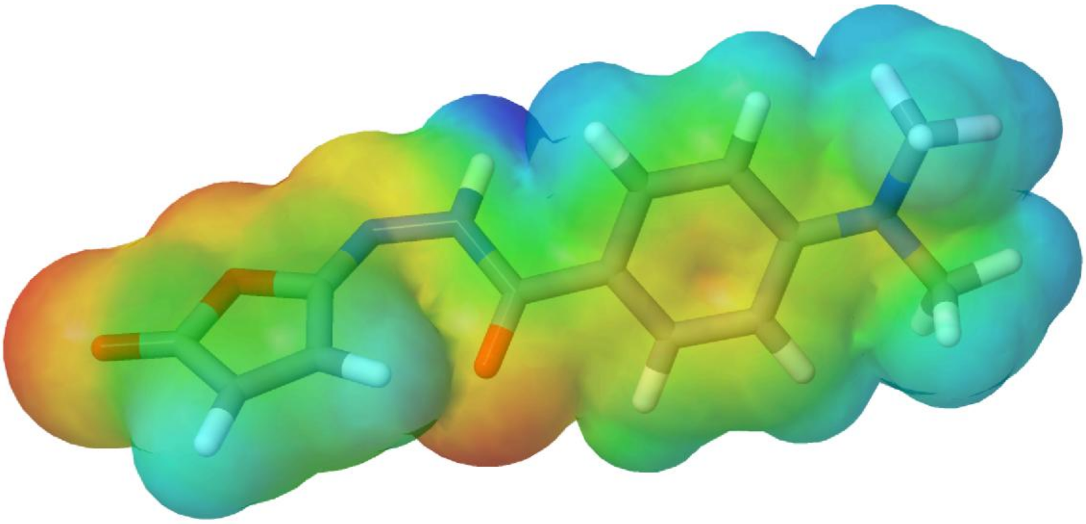

**Figure S10:** Graphical representation molecular electrostatic potential for compound **2d**

LUMO

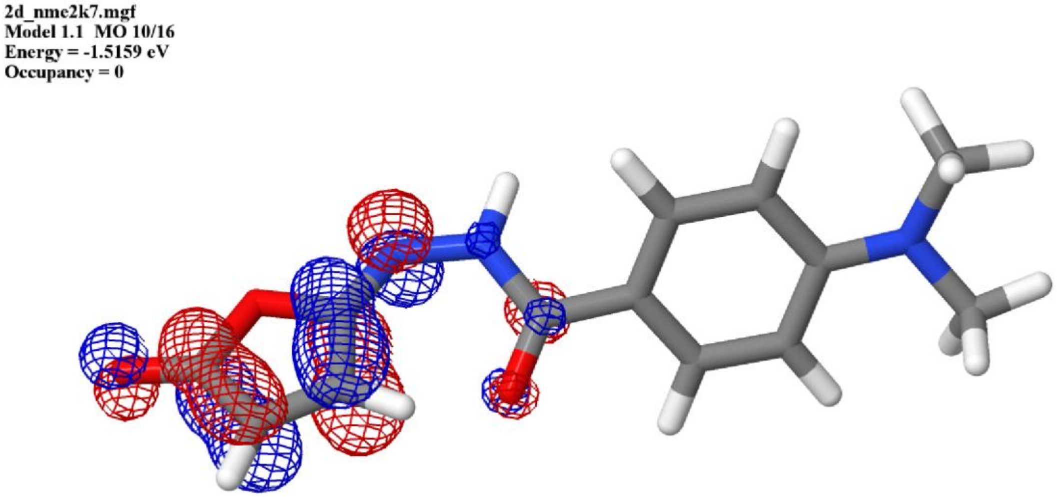


**Figure S11:** Graphical representation LUMO orbital for compound **2d**

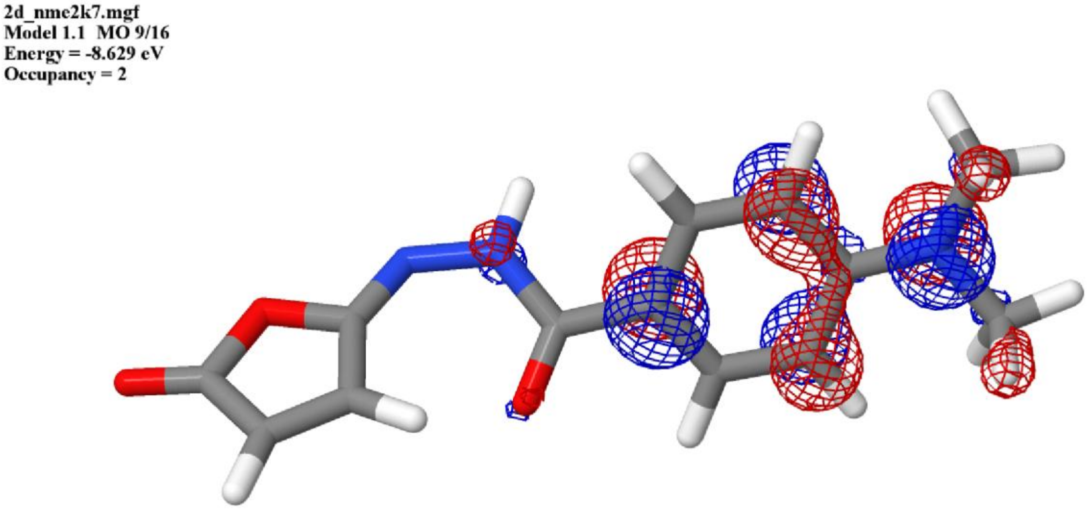


**Figure S12:** Graphical representation HOMO orbital for compound **2d**

### 4-Methoxy-*N*'-(5-oxofuran-2(5*H*)-ylidene)benzohydrazide (2e)

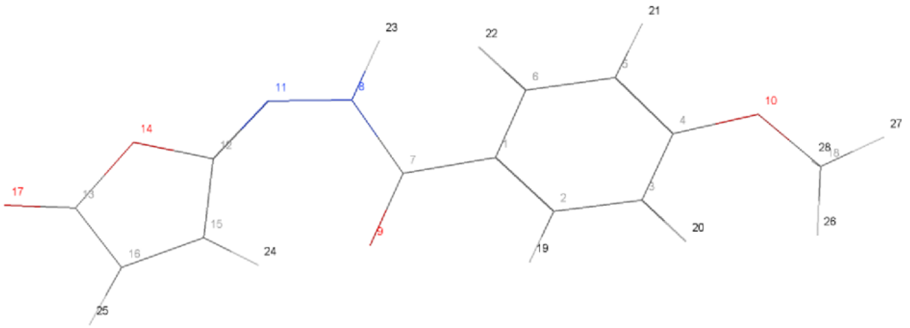

**Table S5:** Heat of formation, atomic coordinates and Net charges for compound **2e**

| ΔHf = -79.61865 kcal/mol | | | | | |
|---|---|---|---|---|---|
| Atom No. | Element | X | Y | Z | Charge |
| 1 | C | -2.506009457 | 2.619469738 | -1.513766588 | -0.252353 |
| 2 | C | -3.657687287 | 2.115131807 | -0.902753804 | -0.000541 |
| 3 | C | -4.771150357 | 2.928198124 | -0.729205868 | -0.323797 |
| 4 | C | -4.715838697 | 4.247491928 | -1.187263260 | 0.304150 |
| 5 | C | -3.566664155 | 4.764359789 | -1.815433463 | -0.286565 |
| 6 | C | -2.465308012 | 3.942096510 | -1.974510680 | -0.027169 |
| 7 | C | -1.366310729 | 1.705592206 | -1.689423862 | 0.605044 |
| 8 | N | -0.101855128 | 2.259946786 | -1.454758789 | -0.404911 |
| 9 | O | -1.463012113 | 0.540810144 | -2.026754921 | -0.579122 |
| 10 | O | -5.714840538 | 5.152089890 | -1.091109463 | -0.345969 |
| 11 | N | 1.061787962 | 1.561344363 | -1.429972887 | -0.216686 |
| 12 | C | 1.321052101 | 0.562591241 | -2.206980372 | 0.282986 |
| 13 | C | 2.812335268 | -1.048854674 | -2.820509638 | 0.618537 |
| 14 | O | 2.598908433 | 0.040128898 | -1.940424659 | -0.394690 |
| 15 | C | 0.734164846 | -0.223512406 | -3.317619899 | -0.064430 |
| 16 | C | 1.614778212 | -1.172833859 | -3.677467194 | -0.260720 |
| 17 | O | 3.847936425 | -1.635894644 | -2.731402910 | -0.448251 |
| 18 | C | -6.933386281 | 4.736811288 | -0.480569417 | -0.201509 |
| 19 | H | -3.682325320 | 1.075376549 | -0.557989472 | 0.177711 |
| 20 | H | -5.663325392 | 2.533688472 | -0.247010046 | 0.186831 |
| 21 | H | -3.559361398 | 5.795162495 | -2.167338903 | 0.187453 |
| 22 | H | -1.569125805 | 4.327915244 | -2.471286909 | 0.176794 |
| 23 | H | 0.005957998 | 3.159513116 | -0.957677481 | 0.370386 |
| 24 | H | -0.240889988 | -0.005757423 | -3.737608080 | 0.214568 |
| 25 | H | 1.543359478 | -1.927239426 | -4.446915299 | 0.216486 |
| 26 | H | -7.398205504 | 3.923602866 | -1.048062657 | 0.149910 |
| 27 | H | -7.546772008 | 5.647865327 | -0.540490497 | 0.166359 |
| 28 | H | -6.771330216 | 4.453801083 | 0.564954217 | 0.149500 |

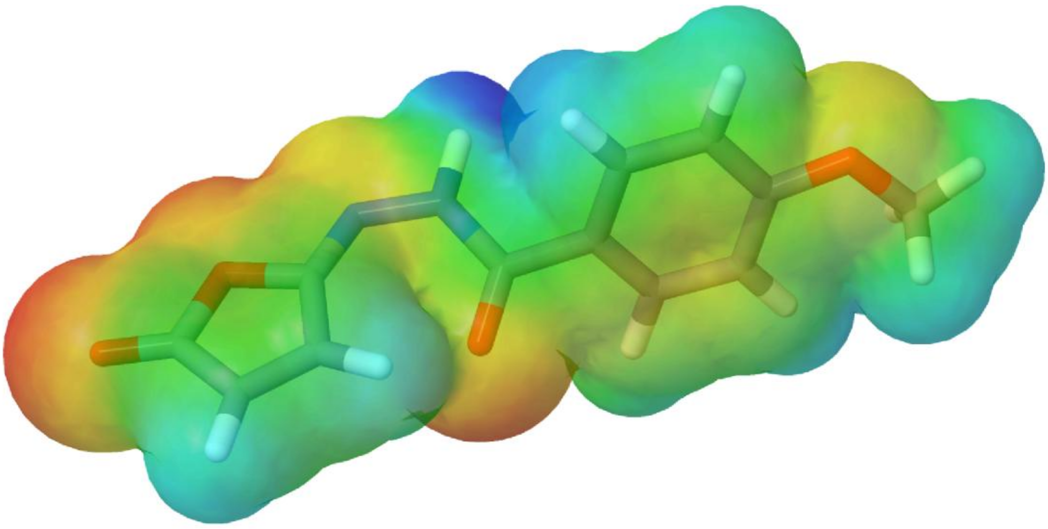

**Figure S13:** Graphical representation molecular electrostatic potential for compound **2e**

LUMO

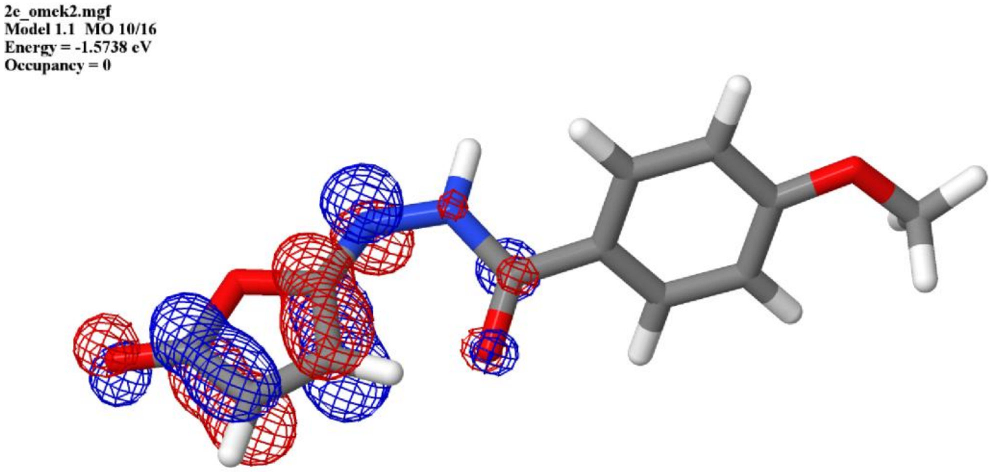


**Figure S14:** Graphical representation LUMO orbital for compound **2e**

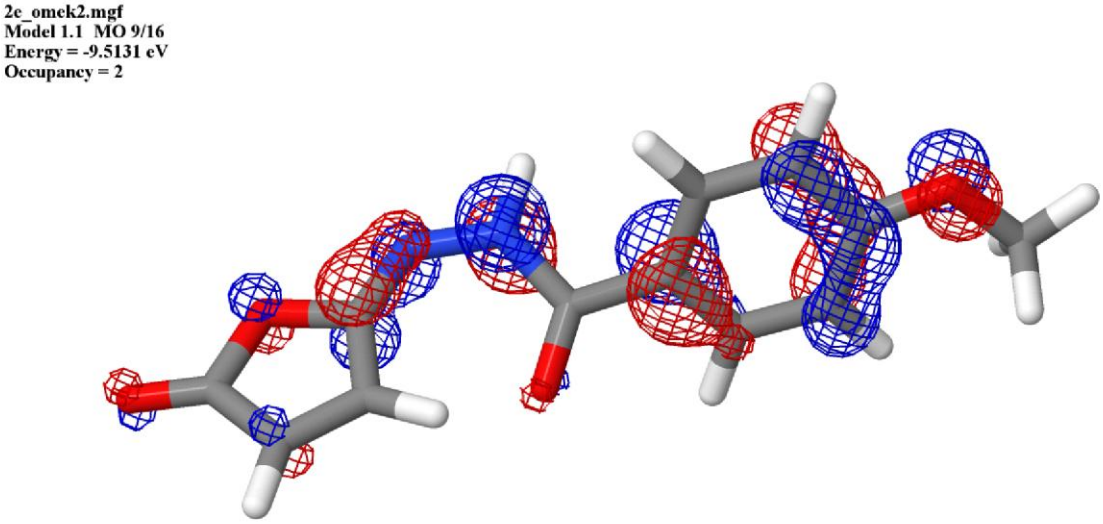


**Figure S15:** Graphical representation HOMO orbital for compound **2e**

## 5-(2-Phenylhydrazineylidene)furan-2(*5H*)-one (2f)

**Table S6:** Heat of formation, atomic coordinates and Net charges for compound **2f**

| ΔHf = 10.56278 kcal/mol | | | | | |
|---|---|---|---|---|---|
| Atom No. | Element | X | Y | Z | Charge |
| 1 | N | 1.936337379 | -0.292358032 | 0.216521163 | -0.354656 |
| 2 | N | 1.134468845 | -0.771609912 | 1.236336034 | -0.263508 |
| 3 | C | 1.654659060 | -1.193024473 | 2.343313264 | 0.295418 |
| 4 | C | 1.467978746 | -1.873779574 | 4.501832827 | 0.605076 |
| 5 | O | 0.734908449 | -1.477800958 | 3.346403340 | -0.373273 |
| 6 | C | 3.020295137 | -1.463403442 | 2.865441272 | -0.135747 |
| 7 | C | 2.906599743 | -1.884093398 | 4.133913437 | -0.198914 |
| 8 | O | 0.853525284 | -2.104766930 | 5.494957866 | -0.418401 |
| 9 | C | 2.142576254 | -1.190074144 | -0.881385011 | 0.152482 |
| 10 | C | 1.363144956 | -2.330884145 | -1.096187704 | -0.223075 |
| 11 | C | 1.577046340 | -3.092532930 | -2.242675839 | -0.105316 |
| 12 | C | 2.551336004 | -2.722805319 | -3.167024381 | -0.201708 |
| 13 | C | 3.336062194 | -1.592424038 | -2.938095678 | -0.103153 |
| 14 | C | 3.142668432 | -0.824169060 | -1.796967853 | -0.243846 |
| 15 | H | 2.813538046 | 0.161142642 | 0.536780545 | 0.296340 |
| 16 | H | 3.906202704 | -1.347168257 | 2.257799588 | 0.204837 |
| 17 | H | 3.664761985 | -2.200516263 | 4.837030627 | 0.218172 |
| 18 | H | 0.601878258 | -2.631640615 | -0.376172450 | 0.181610 |
| 19 | H | 0.983748953 | -3.993558271 | -2.410853165 | 0.165265 |
| 20 | H | 2.710376469 | -3.320795182 | -4.063153311 | 0.164798 |
| 21 | H | 4.116724080 | -1.315749006 | -3.649458282 | 0.163081 |
| 22 | H | 3.767375494 | 0.050108651 | -1.614675656 | 0.174517 |

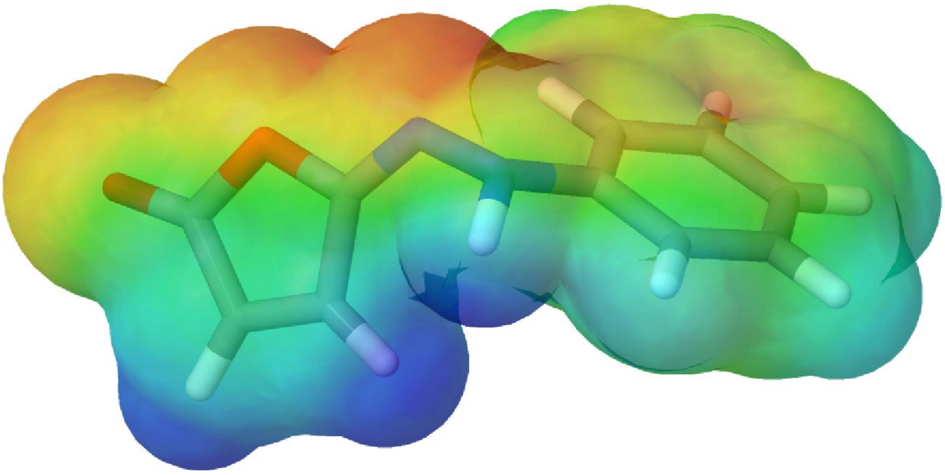

**Figure S16:** Graphical representation of molecular electrostatic potential for compound **2f**

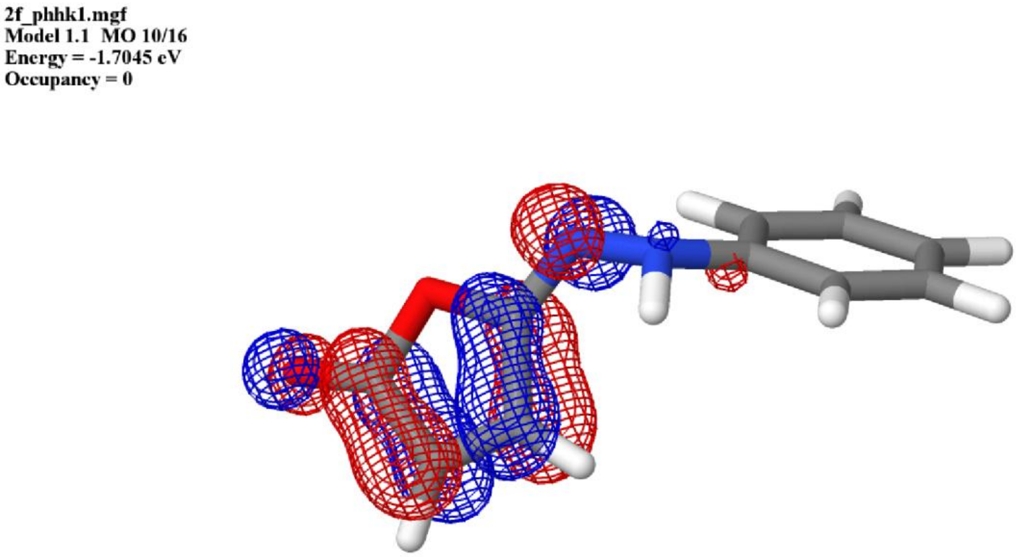


**Figure S17:** Graphical representation of LUMO orbital for compound **2f**

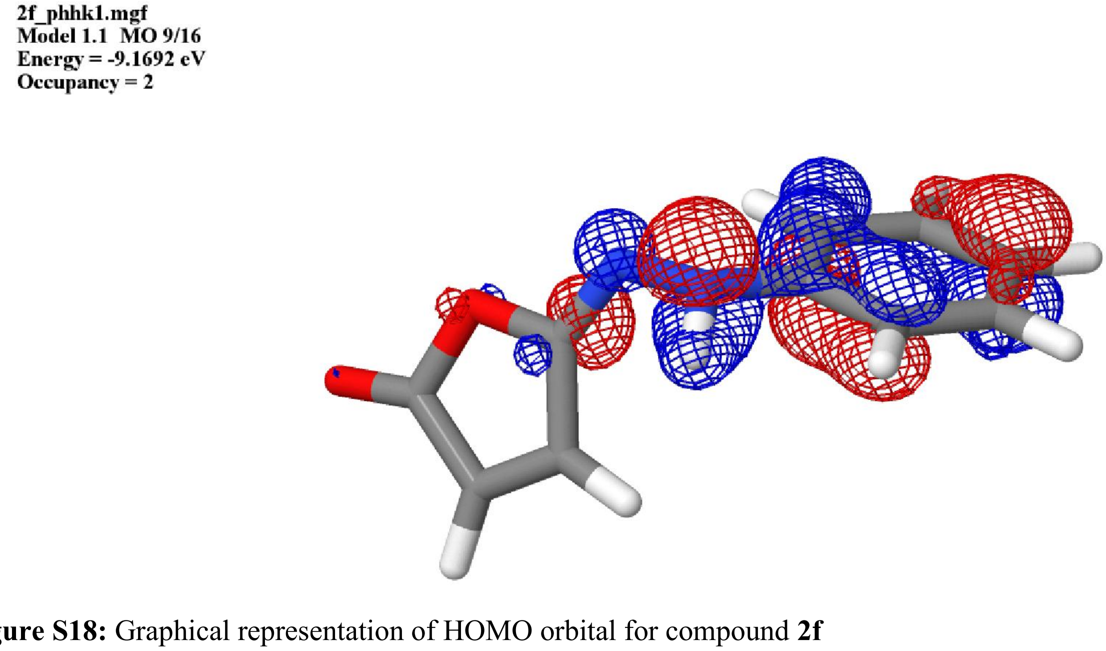


**Figure S18:** Graphical representation of HOMO orbital for compound **2f**

**N'-[5-oxo-2,5-dihydrofuran-2-ylidene]thiophene-2-carbohydrazide (2h)**

**Table S7:** Heat of formation, atomic coordinates and net charges for compound **2h**

| $\Delta H_f$= -28.06851 kcal/mol | | | | | |
|---|---|---|---|---|---|
| Atom No | Element | x | y | z | Charge |
| 1 | O | 0,50333 | -2,9166 | -0,78211 | -0,37475 |
| 2 | C | -0,12238 | -3,97455 | -1,50627 | 0,602352 |
| 3 | C | -1,30993 | -3,40239 | -2,18703 | -0,21296 |
| 4 | C | -1,39186 | -2,1007 | -1,87407 | -0,1184 |
| 5 | C | -0,26246 | -1,76995 | -0,96558 | 0,316244 |
| 6 | O | 0,380073 | -5,05164 | -1,44911 | -0,40975 |
| 7 | N | 0,178959 | -0,74362 | -0,3114 | -0,23698 |
| 8 | N | -0,49694 | 0,451409 | -0,38245 | -0,42544 |
| 9 | C | -1,78925 | 0,671153 | 0,098 | 0,624138 |
| 10 | C | -2,62996 | -0,43885 | 0,518493 | -0,37197 |
| 11 | O | -2,15291 | 1,83807 | 0,105924 | -0,58069 |
| 12 | S | -4,28771 | -0,56677 | 0,003176 | 0,313625 |
| 13 | C | -2,32078 | -1,44268 | 1,402242 | -0,07312 |
| 14 | C | -3,411 | -2,33716 | 1,632285 | -0,20791 |
| 15 | C | -4,53866 | -1,99971 | 0,935138 | -0,21495 |
| 16 | H | -1,94182 | -4,008 | -2,82177 | 0,218232 |
| 17 | H | -2,10002 | -1,35617 | -2,20938 | 0,208704 |
| 18 | H | 0,158378 | 1,248482 | -0,4617 | 0,373274 |
| 19 | H | -1,36188 | -1,56412 | 1,897383 | 0,187015 |
| 20 | H | -3,32519 | -3,18896 | 2,295774 | 0,186359 |
| 21 | H | -5,48427 | -2,52439 | 0,9353 | 0,196959 |

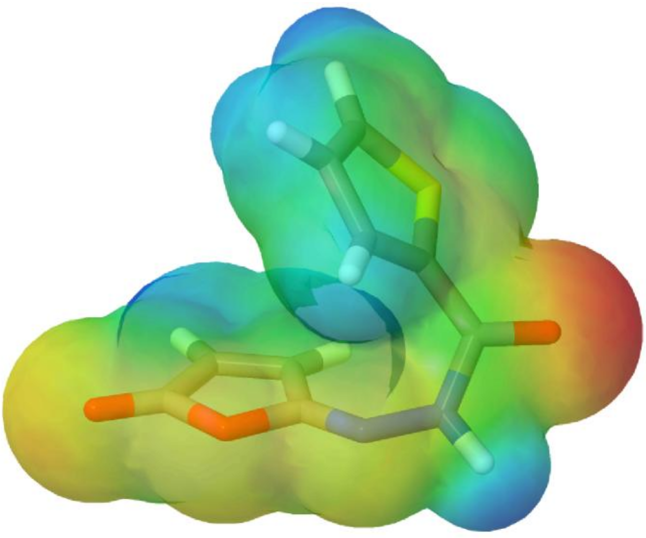

**Figure S19:** Graphical representation of molecular electrostatic potential for compound **2h**

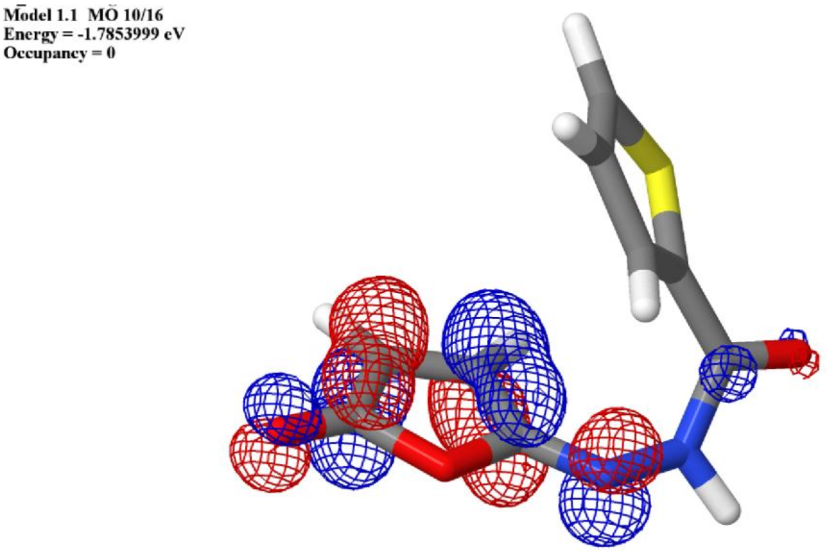


**Figure S20:** Graphical representation of LUMO orbital for compound **2h**

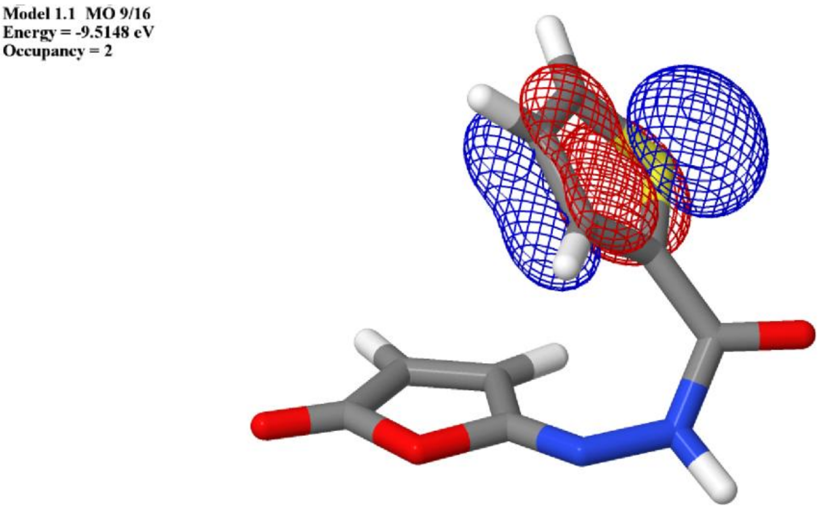


**Figure S21:** Graphical representation of HOMO orbital for compound **2h**

**N'-(3-oxo-3,4,4a,5,5a,6-hexahydro-1H-4,6-ethenocyclopropa[f]isobenzofuran-1-ylidene)-4-(trifluoromethyl)benzohydrazide (4)**

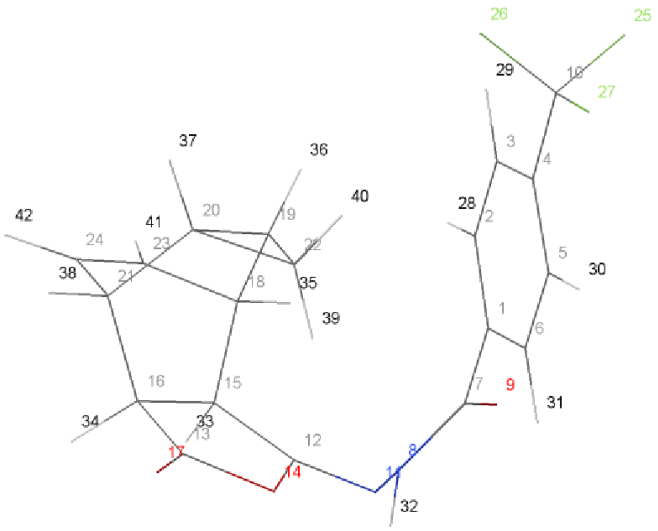

**Table S8:** Heat of formation, atomic coordinates and net charges for compound **4**

| ΔHf = -180.02504 kcal/mol | | | | | |
|---|---|---|---|---|---|
| Atom No. | Element | X | Y | Z | Charge |
| 1 | C | 0.736164350 | -1.571609311 | 0.877008494 | -0.093137 |
| 2 | C | -0.142536648 | -2.266674886 | 1.709714742 | -0.117370 |
| 3 | C | -0.242312948 | -3.650471087 | 1.599273770 | -0.106344 |
| 4 | C | 0.543095645 | -4.322960547 | 0.660609430 | -0.118645 |
| 5 | C | 1.435875874 | -3.632420823 | -0.155744079 | -0.097263 |
| 6 | C | 1.533774489 | -2.245358712 | -0.044378615 | -0.106421 |
| 7 | C | 0.815965565 | -0.098655244 | 1.037655670 | 0.599307 |
| 8 | N | 0.526064563 | 0.747345780 | -0.022380162 | -0.423611 |
| 9 | O | 1.018853705 | 0.431085415 | 2.120637990 | -0.596176 |
| 10 | C | 0.397274339 | -5.812941701 | 0.563918456 | 0.556291 |
| 11 | N | 0.627187376 | 0.295391959 | -1.335129721 | -0.325546 |
| 12 | C | -0.479152443 | 0.044181977 | -1.967005534 | 0.398981 |
| 13 | C | -1.496312603 | -0.589951314 | -3.925222598 | 0.616800 |
| 14 | O | -0.262844129 | -0.320762174 | -3.278494843 | -0.394075 |
| 15 | C | -1.948203372 | 0.117709107 | -1.661974452 | -0.189014 |
| 16 | C | -2.630055875 | -0.270584065 | -3.004036446 | -0.225758 |
| 17 | O | -1.428983799 | -0.980685238 | -5.048145270 | -0.427994 |
| 18 | C | -2.472115225 | -0.797896819 | -0.526139990 | -0.087123 |
| 19 | C | -2.331507098 | -2.294829078 | -0.807829378 | -0.175688 |
| 20 | C | -3.007240079 | -2.663313966 | -2.131550539 | -0.180110 |
| 21 | C | -3.633878793 | -1.427641392 | -2.779364514 | -0.080387 |
| 22 | C | -1.526899009 | -2.879582265 | -1.940178486 | -0.332405 |
| 23 | C | -3.978272275 | -0.567839081 | -0.549959962 | -0.163032 |
| 24 | C | -4.573991682 | -0.889755014 | -1.707495739 | -0.162922 |
| 25 | F | 0.729755658 | -6.459791521 | 1.683831114 | -0.214598 |
| 26 | F | -0.849758589 | -6.218159999 | 0.311656439 | -0.212728 |
| 27 | F | 1.122011603 | -6.415116127 | -0.378482821 | -0.207761 |
| 28 | H | -0.746920331 | -1.730038448 | 2.446417094 | 0.183218 |
| 29 | H | -0.933280929 | -4.192193033 | 2.251858363 | 0.184839 |

| | | | | | |
|---|---|---|---|---|---|
| 30 | H | 2.066434781 | -4.147575084 | -0.886231238 | 0.187646 |
| 31 | H | 2.232603683 | -1.694500734 | -0.683121765 | 0.190950 |
| 32 | H | 0.713135760 | 1.758532512 | 0.064723545 | 0.361024 |
| 33 | H | -2.200866587 | 1.178931886 | -1.385384870 | 0.207056 |
| 34 | H | -3.195521803 | 0.606159001 | -3.426888678 | 0.215916 |
| 35 | H | -2.004336444 | -0.523269372 | 0.440684497 | 0.160203 |
| 36 | H | -2.474230904 | -2.903152800 | 0.089151481 | 0.170167 |
| 37 | H | -3.644631959 | -3.547181669 | -2.217138917 | 0.172156 |
| 38 | H | -4.151393153 | -1.683306033 | -3.727702505 | 0.170221 |
| 39 | H | -0.762180742 | -2.334711876 | -2.469059111 | 0.165474 |
| 40 | H | -1.176142115 | -3.911157109 | -1.839039206 | 0.172863 |
| 41 | H | -4.455837476 | -0.177501698 | 0.332953754 | 0.163043 |
| 42 | H | -5.623626893 | -0.806764128 | -1.934222699 | 0.161953 |

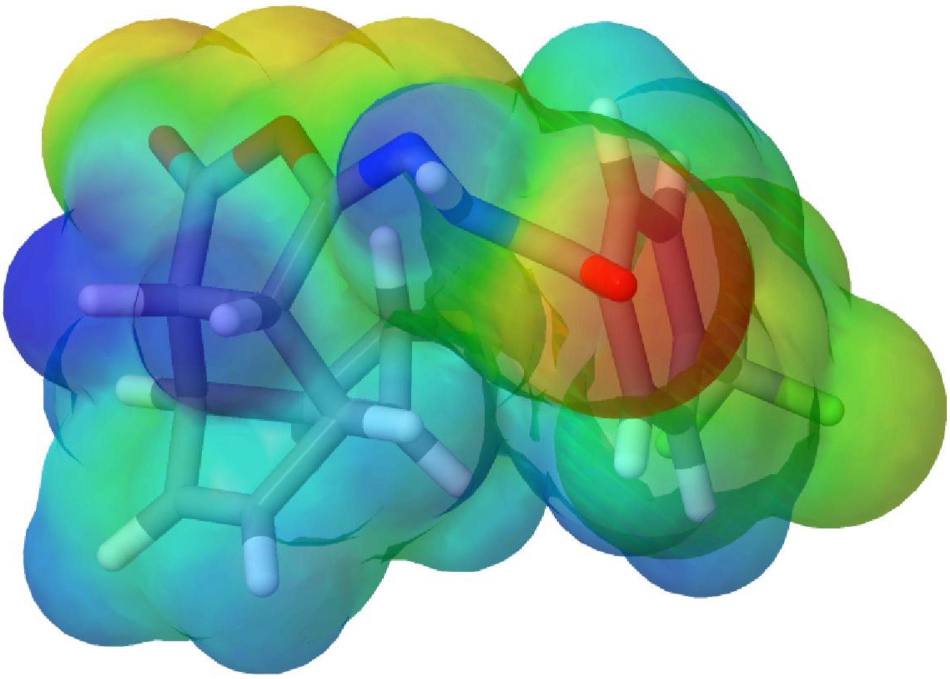

**Figure S22:** Graphical representation of molecular electrostatic potential for compound **4**

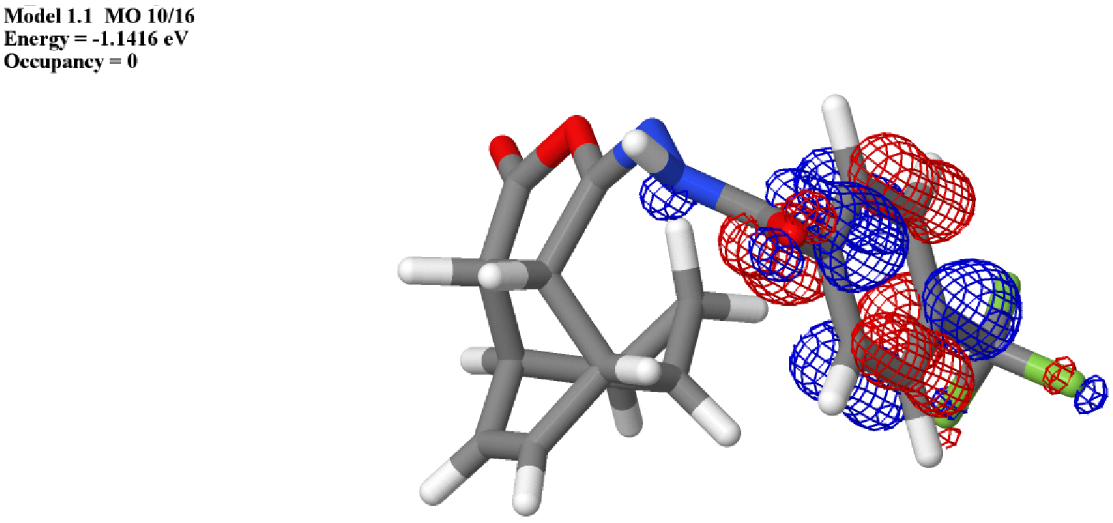


**Figure S23:** Graphical representation of LUMO orbital for compound **4**

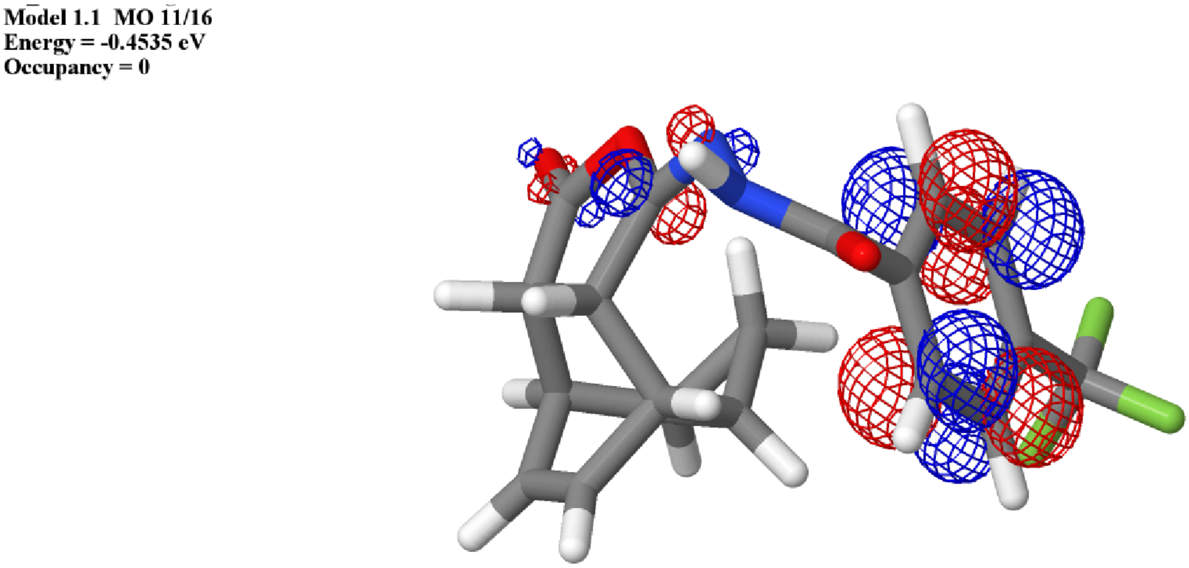


**Figure S24:** Graphical representation of second unoccupied molecular orbital (LUMO+1) for compound **4**

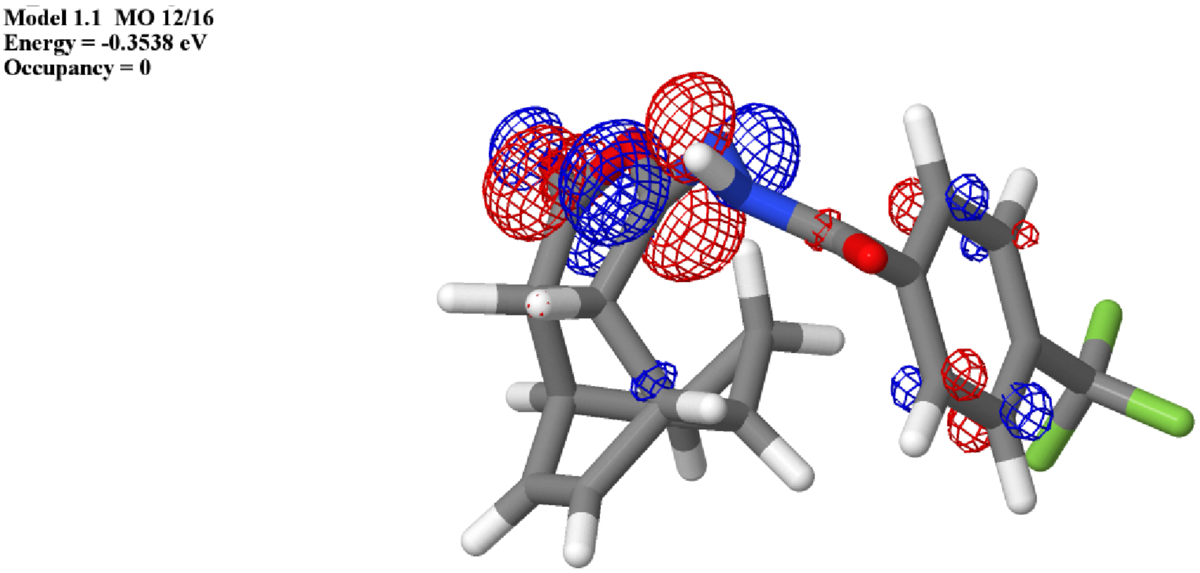


**Figure S25:** Graphical representation of third unoccupied molecular orbital (LUMO+2) for compound **4**

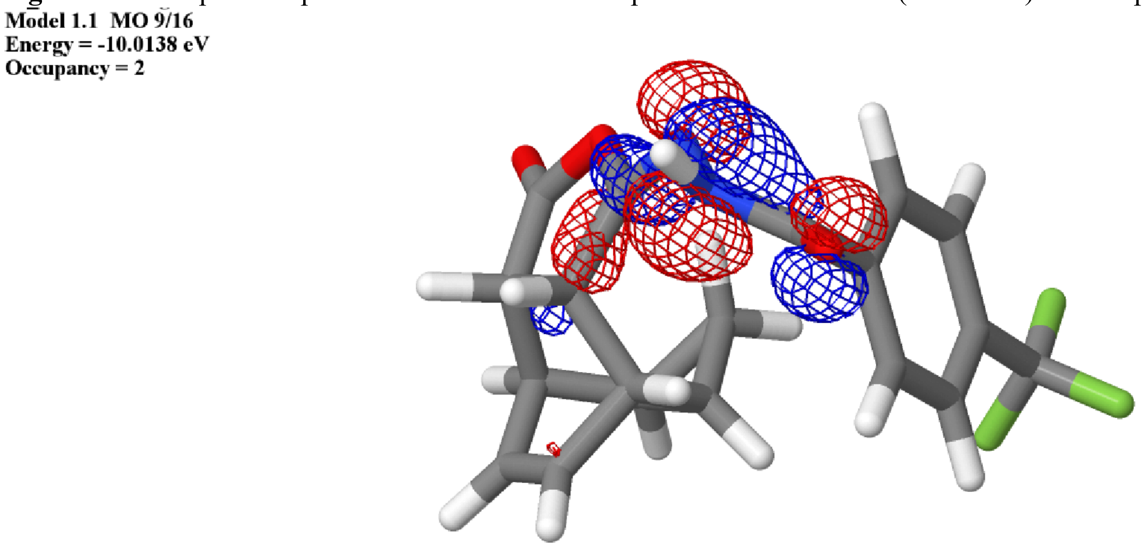


**Figure S26:** Graphical representation of HOMO orbital for compound **4**
